# Supplementary material for: Whole body CT attenuation and volume charts from routine clinical scans via LLM report filtering
Source: NPJ Digit Med. 2026 Jul 3;9:505. doi: 10.1038/s41746-026-02938-2 (PMC13332067; doi:10.1038/s41746-026-02938-2)
Supplement: Supplementary file 1 — Supplementary Information [file 41746_2026_2938_MOESM1_ESM.pdf]

Supplementary Material

Whole-body CT attenuation and volume charts from routine clinical scans via evidence-grounded LLM report filtering

Christian Wachinger, Bernhard Renger, Christopher Späth, Jan Kirschke, Marcus Makowski

Technical University of Munich

Supplementary Note 1: Anatomical structures

Grouping of the 106 anatomical structures from TotalSegmentator.

Organs (23): spleen, kidney right, kidney left, gallbladder, liver, stomach, pancreas, adrenal gland right, adrenal gland left, lung upper lobe left, lung lower lobe left, lung upper lobe right, lung middle lobe right, lung lower lobe right, esophagus, trachea, thyroid gland, small bowel, duodenum, colon, urinary bladder, heart, brain

Bones (58): *Spine*: vertebrae C1, vertebrae C2, vertebrae C3, vertebrae C4, vertebrae C5, vertebrae C6, vertebrae C7, vertebrae T1, vertebrae T2, vertebrae T3, vertebrae T4, vertebrae T5, vertebrae T6, vertebrae T7, vertebrae T8, vertebrae T9, vertebrae T10, vertebrae T11, vertebrae T12 vertebrae L1, vertebrae L2, vertebrae L3, vertebrae L4, vertebrae L5, vertebrae S1, sacrum

Ribs: rib left 1, rib left 2, rib left 3, rib left 4, rib left 5, rib left 6, rib left 7, rib left 8, rib left 9, rib left 10, rib left 11, rib left 12, rib right 1, rib right 2, rib right 3, rib right 4, rib right 5, rib right 6, rib right 7, rib right 8, rib right 9, rib right 10, rib right 11, rib right 12, sternum, costal cartilages

Other: scapula left, scapula right, clavicula left, clavicula right, hip left, hip right

Muscles (8): gluteus maximus left, gluteus maximus right, gluteus medius left, gluteus medius right, gluteus minimus left, gluteus minimus right, iliopsoas left, iliopsoas right

Vessels (17): aorta, pulmonary vein, brachiocephalic trunk, subclavian artery right, subclavian artery left, common carotid artery right, common carotid artery left, brachiocephalic vein left, brachiocephalic vein right, atrial appendage left, superior vena cava, inferior vena cava, portal vein and splenic vein, iliac artery left, iliac artery right, iliac vena left, iliac vena right

Supplementary Note 2: Outlier removal

We use a two-step approach to removing outliers in the segmentation results based on organ volumes. (1) we consider the distribution of volumes of individual structures and (2) we analyze the correlations between organs. In the first step, manually reviewed segmentation data from the TS dataset was processed to remove outliers using robust statistical methods. For each organ, the data underwent a log-transformation (using log1p) to stabilize variance, followed by the computation of robust summary statistics: median and median absolute deviation (MAD). Values were then standardized into z-scores, and those exceeding a predetermined threshold (set here at 0.005) were flagged as outliers. The distribution parameters (median, MAD) derived from this cleaned manual dataset were subsequently used for detecting outliers in the automated volume measurements with a threshold of 0.0001. At this stage, only the most severe outliers are removed (above 4 times the median volume size or below 1/4th the median volume size). This approach ensures that the outlier detection in the automated segmentation is guided by the more trustworthy manual annotations.

In the second step, we exploit the strong correlations that exist among volume measurements of anatomical structures, and we leverage these relationships to identify outliers. First, we extract the underlying correlation structure from the manual data to identify latent factors, captured in the singular vector matrix V, that represent the dominant patterns of inter-organ covariation. The softImpute algorithm is used to perform a low-rank matrix approximation by iteratively computing a singular value decomposition (SVD) and applying soft-thresholding to the singular values, effectively denoising the data and retaining only its most salient features. Next, we project the automated data onto this latent subspace defined by V; deviations from this projection (i.e., residuals) indicate values that do not conform to the expected inter-organ relationships and are flagged as potential outliers.

Supplementary Note 3: LLM-based pathology filtering

Two-stage filtering pipeline

In this supplement we provide the exact prompts used in the two-stage LLM filtering pipeline (Supplementary Figures 1–2) and summarize implementation details required for reproducibility. Filtering relied on open-weight instruction-tuned LLMs obtained from Hugging Face (Qwen2.5, Qwen3, Llama 3.3, OpenBioLLM-70B, and MedGemma-27B). For efficiency, we used quantized/optimized checkpoint variants (e.g., AWQ and FP8-KV formats where available) under otherwise identical prompts and deterministic decoding settings. All models were run with guided decoding against the same JSON schema to ensure consistent, machine-actionable outputs across model families.

Stage 1 performs schema-constrained extraction of potentially abnormal structures from free-text radiology reports. Reports are processed in their native language (English or German) and models are instructed to (i) restrict outputs to a fixed set of 39 canonical anatomical targets (KANON; Supplementary Fig. 1), (ii) flag only structures explicitly described as abnormal, and (iii) ground each abnormality by copying the supporting sentence verbatim from the report. Decoding was deterministic (`temperature`=0, `top_p`=1) and executed with vLLM. To ensure machine-actionable outputs and reduce formatting errors, we used guided decoding with an explicit JSON schema (array of objects), requiring the fields `Structure_ID` (integer), `Structure_KANON` (string), `Structure_Report` (string), `Description` (string; verbatim evidence sentence), and `State` (enum: `normal-abnormal`), and disallowing additional keys. Guided decoding was implemented via the `xgrammar` backend. Although models were instructed to output only abnormal structures, occasional `State="normal"` entries were produced and were removed during post-processing; multiple mentions of the same structure were consolidated by `Structure_ID`.

Stage 2 adjudicates non-unanimous findings via evidence-only cross-verification. A structure was considered *unanimous abnormal* in stage 1 if it was flagged abnormal by all five models (5/5) and was excluded without further adjudication. A structure was considered *disputed* if it was flagged abnormal by at least one but not all models (i.e., ≥ 1 and < 5 votes). For each disputed structure, we aggregated and deduplicated all evidence sentences produced in stage 1 across models, removed model identities, and presented only the structure identifier, canonical structure name, and the evidence sentences to the verifier. The original report text was intentionally withheld at this stage to constrain decisions to explicitly cited evidence and reduce over-interpretation. Verification applied a strict two-part criterion: at least one evidence sentence had to (i) unambiguously match the target anatomy and (ii) clearly state a pathological or non-physiologic finding; purely negative/normal statements were insufficient. In the operational pipeline, final abnormality labels were taken from the verification-stage output of MedGemma-27B (text-only), supplemented by the unan-

Supplementary Figure 1: Initial LLM analysis prompt used to extract abnormal structures from radiology reports.

```
Analyze the radiology report based only on the text.
Goal: List ONLY structures from the KANON LIST as ABNORMAL that are explicitly described as pathological.

Output: strict JSON schema (the only allowed output)
[
  {
    "Structure_ID": <int>,
    "Structure_KANON": "<Name as in KANON>",
    "Structure_Report": "<Name as written in the report>",
    "Description": "<exact sentence from the report>",
    "State": "normal" | "abnormal"
  }
]

Rules:
1. Only structures from the KANON LIST.
2. Use names exactly as in KANON or as in the report.
3. Description must be copied exactly from the report.
4. State of the structure:
  "abnormal" if pathological/suspicious/needs clarification;
  "normal" if not mentioned or described as unremarkable/within normal limits/no evidence/no sign.
5. Output only ABNORMAL structures.
6. If there are no abnormal structures: output [].

KANON LIST (ID: exact text)
1:"liver"; 2:"gallbladder"; 3:"spleen"; 4:"pancreas"; 5:"kidneys"; 6:"kidney cysts";
7:"adrenal glands"; 8:"stomach"; 9:"duodenum"; 10:"small intestine"; 11:"colon";
12:"lungs"; 13:"trachea"; 14:"esophagus"; 15:"heart"; 16:"aorta"; 17:"pulmonary veins";
18:"brachiocephalic trunk"; 19:"subclavian artery"; 20:"common carotid artery";
21:"brachiocephalic vein"; 22:"vena cava"; 23:"portal vein and splenic vein";
24:"common iliac artery"; 25:"common iliac vein"; 26:"thyroid gland"; 27:"vertebrae";
28:"sacrum"; 29:"ribs"; 30:"sternum"; 31:"costal cartilages"; 32:"brain";
33:"clavicle"; 34:"scapula"; 35:"hip"; 36:"gluteus muscles"; 37:"iliopsoas muscle";
38:"atrial appendage"; 39:"urinary bladder"

Radiology Report: <report>
```

imous abnormalities from stage 1. All structures labeled abnormal by this framework were excluded from downstream image analysis and normative modeling.

Region-stratified validation of report filtering

To assess whether LLM-based pathology filtering varied across anatomical regions, we performed an additional region-stratified evaluation using the manual structure-level annotations. The 39 canonical report-filtering targets were grouped into seven broader anatomical regions:

- **Abdominal solid organs:** liver, gallbladder, spleen, pancreas.
- **Cardiovascular:** heart, aorta, pulmonary veins, brachiocephalic trunk, subclavian artery, common carotid artery, brachiocephalic vein, vena cava, portal/splenic veins, common iliac vessels, atrial appendage.
- **Gastrointestinal:** stomach, duodenum, small intestine, colon, esophagus.
- **Head/neck:** thyroid gland, brain.

Supplementary Figure 2: LLM-based verification prompt used to keep only evidence-supported abnormal structures.

```
ROLE
You are a strict verifier for radiology findings. Decide, for each candidate structure, whether it should be KEPT as ABNORMAL
      based ONLY on the provided evidence sentences ("Descriptions").

INPUT
You receive a JSON array of objects. Each object has:
{
  "Structure_ID": <int>,
  "Structure_KANON": "<exact KANON structure name in English>",
  "Descriptions": ["<verbatim sentence/phrase from the report>", "..."]
}

TASK (evaluate EACH object independently)
KEEP the Structure_ID only if AT LEAST ONE description satisfies BOTH tests:

1) ANATOMY MATCH
   The description clearly refers to the SAME anatomy as Structure_KANON.
   - Allow: exact synonyms; standard parent/child relations; regional adjectives
   - Disallow: different organs/structures

2) ABNORMALITY
   The description clearly states a pathological or non-physiologic finding
   - NOT abnormal: purely normal/negative statements ("unremarkable", "normal", "within normal limits" "age-appropriate", "no
      evidence").

DECISION LOGIC
- If ANY single description satisfies BOTH (1) and (2), KEEP the Structure_ID.
- If descriptions conflict, a single qualifying positive is sufficient to KEEP.
- If no description qualifies, do NOT keep the Structure_ID.

OUTPUT (STRICT)
Return ONLY a JSON array of integers with the Structure_ID values to KEEP as abnormal.
If none qualify, return [].
No extra keys, text, or comments.

Disputed Report: <disputed report>
```

- **Musculoskeletal:** vertebrae, sacrum, ribs, sternum, costal cartilages, clavicle, scapula, hip, gluteus muscles, iliopsoas muscle.
- **Thorax/airways:** lungs, trachea.
- **Urinary/adrenal:** kidneys, kidney cysts, adrenal glands, urinary bladder.

For each region, we computed Jaccard overlap, precision, and recall against the manual annotations. To remain consistent with the main validation analysis, results are shown for selected representative strategies: MedGemma Stage 1 and Stage 2, Llama Stage 1 and Stage 2, and majority voting before and after verification.

Supplementary Figure 3 shows the region-stratified results, where bars represent dataset-size–weighted performance across the annotated datasets. Performance varied across anatomical regions and methods. MedGemma Stage 2 improved substantially over MedGemma Stage 1 across all regions, with Jaccard overlap increasing from 0.241–0.528 to 0.536–0.818 and recall increasing from 0.496–0.845 to 0.845–0.943. The strongest agreement was observed for thorax/airways and abdominal solid organs, where MedGemma Stage 2 reached Jaccard values of 0.818 and 0.758, respectively. Lower Jaccard overlap was observed for cardiovascular and musculoskeletal structures, consistent with more variable anatomical specificity in reports and

broader canonical target definitions.

Recall is the most relevant metric for pathology-reduced cohort construction, because false negatives allow abnormal structures to remain in the reference cohort. MedGemma Stage 2 maintained high recall across all regions and exceeded Llama Stage 1 in recall for every anatomical group. For example, this recall advantage was pronounced in cardiovascular structures (0.943 vs. 0.756), urinary/adrenal structures (0.848 vs. 0.660), and thorax/airways (0.938 vs. 0.815). Although Llama Stage 1 achieved high global projected recall in the main analysis, the region-stratified target-level analysis showed less uniform sensitivity across anatomy. Consistent with this, across all annotated target-level decisions, MedGemma Stage 2 showed higher micro-averaged performance than Llama Stage 1, with recall increasing from 0.798 to 0.918, precision from 0.702 to 0.735, and F1 from 0.747 to 0.816. These results support the selection of MedGemma Stage 2 as the final filtering strategy, while also showing that report-filtering performance depends on anatomical region and should be re-evaluated when deploying the pipeline in new reporting settings.

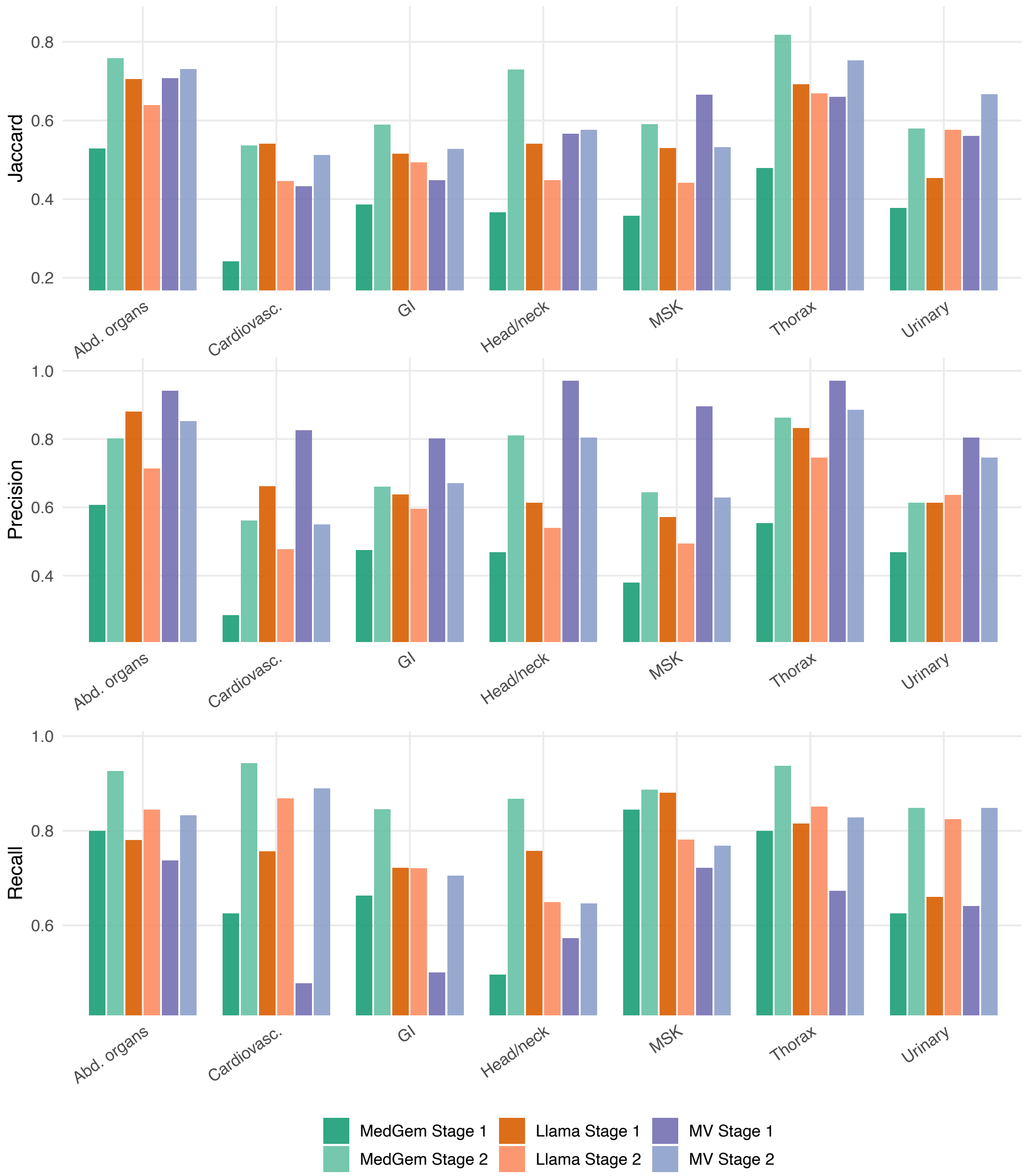

Supplementary Figure 3: Region-stratified validation of LLM-based report filtering. Performance was evaluated across seven anatomical regions after grouping the 39 canonical report-filtering targets into broader anatomical categories. Bars show dataset-size–weighted performance for six selected filtering strategies: MedGemma Stage 1, MedGemma Stage 2, Llama Stage 1, Llama Stage 2, majority vote Stage 1, and majority vote Stage 2. The three panels show Jaccard overlap (top), precision (middle), and recall (bottom) against manual structure-level annotations. MedGemma Stage 2 showed consistently high recall across anatomical regions, supporting its use as the final high-sensitivity filtering strategy for pathology-reduced cohort construction, while precision and Jaccard varied more strongly across regions.

Supplementary Note 4: GAMLSS modeling

Fractional Polynomials

Fractional polynomials of degree m for a covariate x are defined as:

$$FP(x) = \beta_0 + \beta_1 T_{p_1}(x) + \beta_2 T_{p_2}(x) + \cdots + \beta_m T_{p_m}(x),$$

where we consider fractional polynomials up to degree 3. The transformation $T_p(x)$ is defined as:

- $T_p(x) = x^p$, if $p \neq 0$
- $T_p(x) = \log(x)$, if $p = 0$,

where the common set of candidate powers is: $p \in \{-2, -1, -0.5, 0, 0.5, 1, 2, 3\}$.

If the best-fitting model requires the same power to appear more than once (for example, $p_1 = p_2$), the second occurrence is modified to include a logarithmic factor. In this case, the two terms become:

- $\beta_1 \, x^p$
- $\beta_2 \, x^p \log(x)$.

This modification ensures that the model remains identifiable and flexible even when the same power is selected more than once.

ST1 model family for CT attenuation

Supplementary Figure 4 demonstrates the necessity of modeling CT attenuation with the ST1 family. The distribution of lung attenuation is markedly right-skewed ($\nu = 14.3$), whereas distributions for iliopsoas and esophagus are left-skewed. Lung, aorta, and esophagus exhibit very heavy tails with $\tau < 2$. These distributional characteristics cannot be adequately captured by the Gaussian distribution, which would yield misleading centile estimates. In contrast, the ST1 family accounts for both skewness and kurtosis, providing substantially improved fits.

GAMLSS model diagnostics

In addition to the bootstrap resampling results on model stability, we assessed goodness-of-fit for the CT attenuation models using detrended transformed Owen's plots (DTOPs) and standard Q–Q plots of the quantile residuals. DTOPs are based on a nonparametric procedure that derives uncertainty bands directly

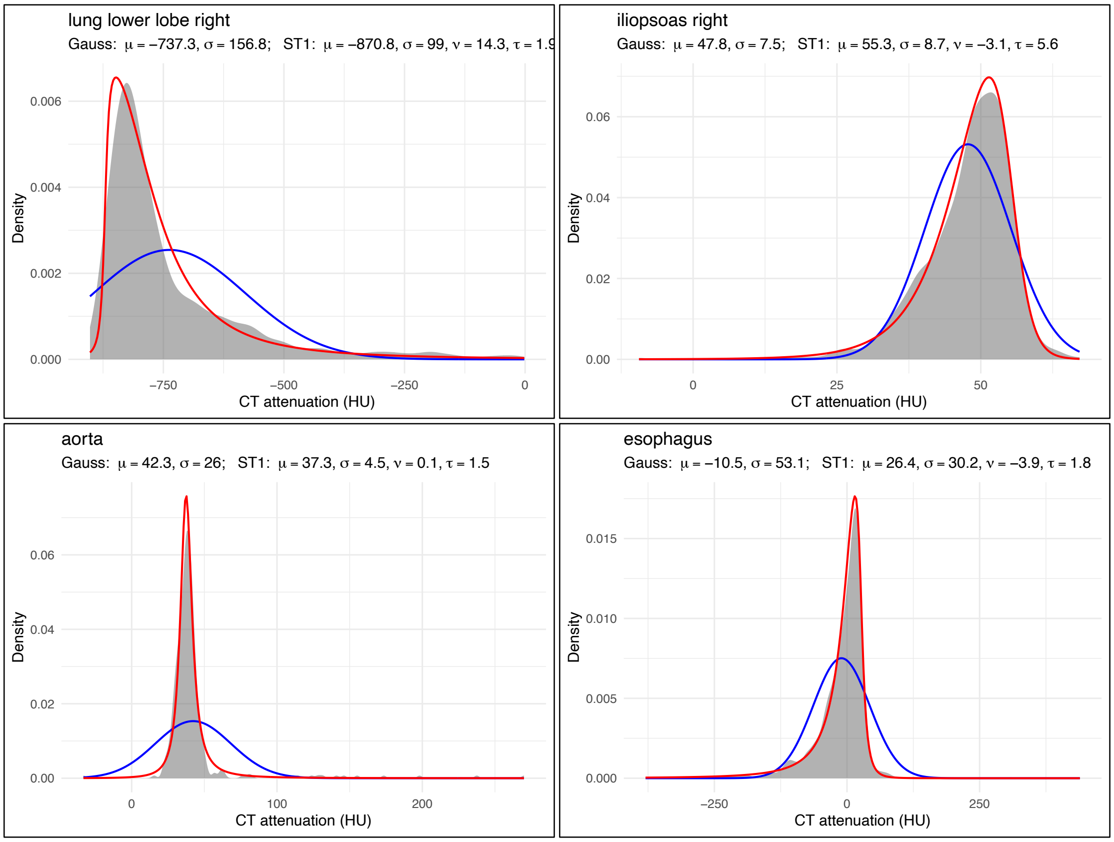

Supplementary Figure 4: Distribution of CT attenuation for the lung lower right lobe, the right iliopsoas, the aorta, and the esophagus. Kernel density estimates (gray) are overlaid with the best-fitting Gaussian (blue) and ST1 (red) distributions. Estimated distribution parameters are shown at the top of each panel. The lung exhibits positive (right) skewness ($\nu = 14.3$), whereas the iliopsoas ($\nu = -3.2$) and esophagus ($\nu = -3.9$) show negative (left) skewness. Values of $\tau < 2$ indicate very heavy tails and are observed for lung ($\tau = 1.9$), aorta ($\tau = 1.5$), and esophagus ($\tau = 1.8$).

from the empirical residual distribution. For the selected organs shown in Supplementary Figure 5, the 95% confidence bands of the ordered centile residuals consistently include zero, which is consistent with approximately normally distributed residuals. The Q–Q plots provide complementary evidence, showing close agreement between empirical residual quantiles and those expected under normality. Taken together, these diagnostics indicate that the distributional assumptions were reasonable and that the fitted attenuation models provide an adequate description of the data.

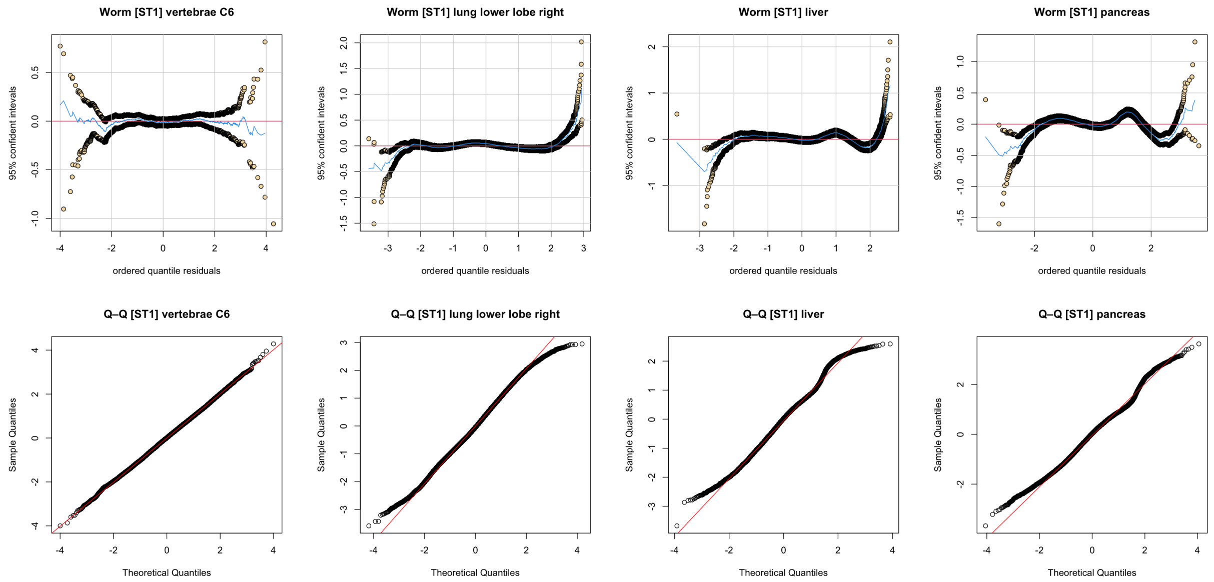

Supplementary Figure 5: Top: Detrended transformed Owen's plots (DTOPs) of the ordered centile residuals for selected organs. The shaded 95% confidence intervals consistently crossing the zero line indicate that the residuals are approximately normally distributed, supporting the adequacy of the fitted skew-t (ST1) distributions. Bottom: Normal Q–Q plots of the quantile residuals for selected organs. The residual points closely follow the red reference line, indicating that the bulk of the distribution conforms to normality and that the model provides an adequate fit across all quantiles.

Supplementary Note 5: Cohort statistics

Supplementary Table 1 summarizes key demographic and acquisition characteristics of the final normative modeling cohort stratified by data source, including age, tube voltage (kVp), sex distribution, scanner manufacturer, and the proportion of contrast-enhanced versus non-contrast examinations. Subjects with missing data were excluded. The cohort comprised 276,132 scans from 177,014 participants, totaling 9,179,609 structure segmentations. Across studies, the cohort spans a wide adult age range and includes data from major CT vendors, reflecting the heterogeneity expected in multi-center retrospective collections. The modality composition differs by dataset design: CT-Rate consists exclusively of non-contrast chest CT, INSPECT comprises contrast-enhanced CTPA studies, and Merlin predominantly contains contrast-enhanced abdominal CT, whereas the PACS (TUM) and TotalSegmentator collections include both contrast and non-contrast examinations.

To characterize the longitudinal subset, we quantified three complementary properties (Supplementary Figure 6). First, we assessed the distribution of the number of scans per individual, which is right-skewed with most repeatedly imaged individuals contributing a small number of examinations and a progressively smaller fraction contributing many follow-up scans (Supplementary Figure 6a). Second, we computed per-individual follow-up spans as the difference between the last and first scan date, showing that most follow-up periods are short, while a subset of individuals exhibit multi-year follow-up (Supplementary Figure 6b). Third, we examined inter-scan intervals as the elapsed time between consecutive scans within individuals, which similarly follows a heavy-tailed distribution, reflecting a mixture of short-term re-imaging (e.g., within the same clinical episode) and longer-term follow-up examinations (Supplementary Figure 6c). For visualization, histograms are displayed with a logarithmic y-axis to capture the wide dynamic range of counts.

Supplementary Table 1: Final cohort characteristics by study. Values are mean (SD) for continuous variables (Age in years; tube voltage, kVp) and percentages for categorical variables (sex distribution, scanner manufacturer, and contrast status). Manufacturer percentages indicate the proportion of scans acquired on CT systems from Philips, Siemens, GE, or Toshiba within each study. Contrast status is shown as the proportion of non-contrast (No-Cont) and contrast-enhanced (With-Cont) examinations.

Study	Age	Tube Volt.	Female	Male	Philips	Siemens	GE	Toshiba	No-Cont	With-Cont
TUM	61.4 (17.4)	118.9 (6.3)	43.3 %	56.7 %	73.8 %	26.0 %	0.1 %	0.0 %	39.1 %	60.9 %
TS	62.8 (14.7)	112.6 (13.4)	41.1 %	58.9 %	12.0 %	83.8 %	4.2 %	0.0 %	30.1 %	69.9 %
CTrate	48.1 (17.1)	104.1 (8.5)	43.4 %	56.6 %	67.2 %	32.8 %	0.0 %	0.0 %	100.0 %	0.0 %
INSPECT	59.1 (17.0)	106.4 (12.6)	56.1 %	43.9 %	0.0 %	65.2 %	15.9 %	18.8 %	0.0 %	100.0 %
Merlin	54.8 (18.4)	112.1 (11.2)	56.5 %	43.5 %	0.0 %	39.0 %	61.0 %	0.0 %	0.1 %	99.9 %

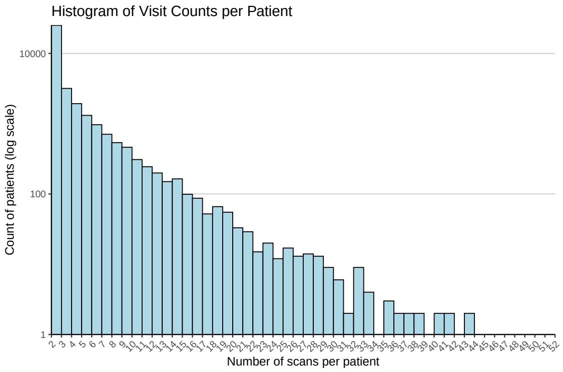

(a) Distribution of scan counts per patient (log-scaled y-axis).

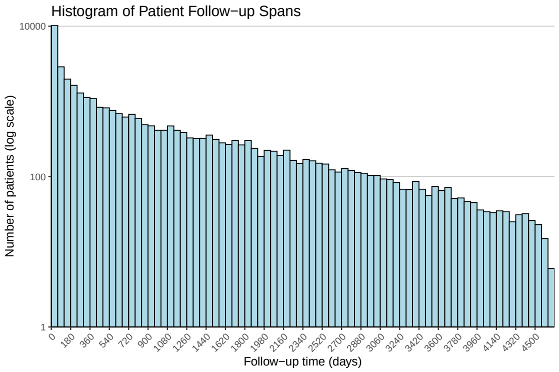

(b) Distribution of patient follow-up spans (last minus first scan date; log-scaled y-axis).

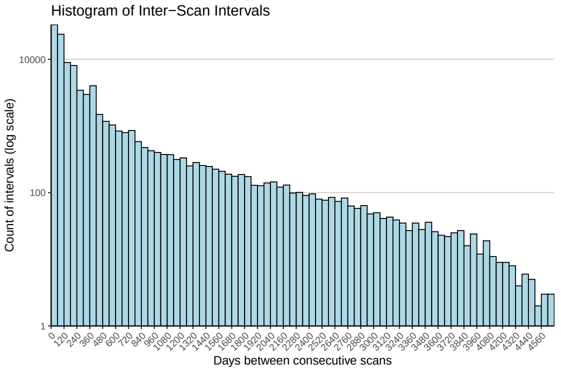

(c) Distribution of inter-scan intervals between consecutive scans (log-scaled y-axis).

Supplementary Figure 6: Longitudinal sample characteristics. (a) Number of scans per patient among individuals with repeat imaging. (b) Total follow-up time per patient (difference between last and first scan date). (c) Time between consecutive scans within patients (inter-scan intervals). All histograms use a logarithmic y-axis to visualize the heavy-tailed distributions.

Supplementary Note 6: Comparison of longitudinal HU changes to prior reports

In this section, we contextualize the longitudinal attenuation changes estimated by our models by comparing them to prior CT reports in the literature. For parenchymal organs, our non-contrast reference curves reproduce the widely reported age-associated attenuation decline that is commonly attributed to progressive fat infiltration [1, 2]. In the longitudinal unenhanced liver study by Hahn et al. [2], liver attenuation was measured on CT colonography screening examinations in 1022 asymptomatic adults (556 women, 466 men; mean age at index CT, 56.7 years) with a mean surveillance interval of 5.5 ± 0.8 years. Mean liver attenuation decreased from 60.3 HU at baseline to 58.4 HU at follow-up, i.e., $\Delta = -1.9$ HU over 5.5 years, corresponding to an average change of ≈ -0.35 HU/year. Evaluating our longitudinal non-contrast liver model at the same mean age (56.7 years; Age.bl scaled by 10), the implied within-subject slope is steeper (female: $-2.1696 + 5.67 \cdot 0.2372 = -0.82$ HU/year; male: $-0.82 + 0.0548 = -0.77$ HU/year), consistent with stronger drift in our clinically indicated PACS follow-up cohort than in an asymptomatic screening population.

In skeletal muscle, attenuation declines with age due to increasing intramuscular fat (myosteatosis), with higher values in men in many cohorts [3, 4]. Graffy et al. [4] analyzed a longitudinal adult screening cohort acquired without intravenous contrast (CT colonography; mean age 57.1 years; mean follow-up ≈ 5.0 years) and reported mean attenuation changes of -1.47 HU/year in men and -1.43 HU/year in women for their abdominal skeletal muscle compartment at the L3 level. Mapping this to our TotalSegmentator-derived targets, the closest anatomical analogue is the iliopsoas (left/right), which captures a major component of the L3 abdominal muscle region but is narrower than an aggregate "abdominal skeletal muscle" definition that typically includes additional paraspinal and abdominal wall muscles. At the study's mean age, our non-contrast iliopsoas slopes are smaller in magnitude (iliopsoas left: female -0.54 HU/year, male -0.78 HU/year; iliopsoas right: female -0.48 HU/year, male -0.77 HU/year), and our contrast-enhanced iliopsoas slopes are further attenuated (iliopsoas left: female -0.39 HU/year, male -0.54 HU/year; iliopsoas right: female -0.38 HU/year, male -0.47 HU/year). These differences are compatible with the narrower target definition and with protocol and selection differences between screening cohorts and indication-driven follow-up imaging. Consistent with Boutin et al. [5], we also observe contrast-dependent shifts in muscle attenuation that motivate separate modeling by contrast state.

For bone, trabecular vertebral attenuation decreases with age and serves as an opportunistic correlate of bone mineral density [6]. Jang et al. [6] reported that mean L1 trabecular attenuation decreased approximately linearly with age at a rate of 2.5 HU/year in a large cohort that mixed women and men as

well as contrast-enhanced and non-contrast examinations. Using age 60 years to parameterize our longitudinal L1 models, the implied within-subject slopes span a wide range across sex and contrast state (non-contrast: female −4.06 HU/year, male −2.08 HU/year; contrast-enhanced: female −2.49 HU/year, male −0.87 HU/year). Averaging across these four strata to mirror a mixed cohort yields a mean slope of −2.38 HU/year, closely matching the −2.5 HU/year rate reported by Jang et al. and illustrating how protocol and covariate mixing can produce aggregate attenuation-change rates that align with prior population estimates.

Regression Coefficients for 8 Selected Structures

Supplementary Tables 2–3 summarize the regression coefficients for the eight selected structures for (i) the GAMLSS volume models (GG distribution) and (ii) the GAMLSS attenuation models (ST1 distribution). For volumes (Supplementary Table 2), effects are reported as $\exp(\hat{\beta})$ for both μ and σ, such that values > 1 indicate increases relative to the reference level. For attenuation (Supplementary Table 3), μ coefficients are additive intensity effects and σ coefficients are reported as $\exp(\hat{\beta})$. Boldface indicates Bonferroni-adjusted significance across all models; full coefficient tables for all structures are provided in Supplementary Tables 4–5. Missing entries indicate that age terms were not selected by the FP BIC, or that scanner manufacturers were not present.

Supplementary Table 2: GAMLSS volume (GG) coefficient estimates. Columns are grouped by distribution parameters (μ and σ). For μ, estimates represent multiplicative effects on the location parameter: Age terms (Age-1, Age-2; fractional-polynomial basis coefficients), Sex (male vs female), kVp (per 10 kVp increase), Contrast (contrast-enhanced vs non-contrast), and manufacturer indicators (GE, Siemens, Toshiba vs the reference manufacturer). For σ, estimates analogously summarize effects on the scale parameter (Age-1, Age-2; Sex). Estimates are shown as $\exp(\hat{\beta})$; values > 1 indicate an increase and values < 1 a decrease in the respective parameter relative to the reference. Boldface marks Bonferroni-adjusted $p < 0.05$. Regression coefficients for all structures are reported in Supplementary Table 4.

	μ								σ		
Structure	Age-1	Age-2	Sex	kVp×10	Contr	GE	SIE	TOS	Age-1	Age-2	Sex
aorta	**1.04**	**1.00**	**1.34**	1.00	**0.98**	1.06	**0.91**		**0.97**	**1.00**	1.01
gluteus minimus r	**1.65**	**0.60**	**1.34**	**1.01**	1.00	**0.98**	1.00		**0.98**	**1.01**	0.98
heart	**0.79**	**1.00**	**1.30**	**1.01**	**1.01**	**0.94**	**0.97**	**0.95**	**1.00**		**1.04**
kidney right	**0.47**	**1.00**	**1.26**	**1.02**	**1.07**	0.99	1.00				**0.95**
liver	**1.57**	**0.99**	**1.18**	**1.01**	**1.02**	**0.96**	**0.97**	0.95	**1.00**		**0.96**
lung upper lobe r	**1.02**	**1.00**	**1.35**	**1.01**	**1.08**	**1.15**	1.00	1.01	**0.91**	**1.00**	1.03
spleen	**0.80**	**1.00**	**1.32**	**1.03**	**1.07**	**0.93**	**0.95**	**0.91**	**1.01**	**1.00**	1.02
vertebrae L3	**1.14**		**1.29**	1.00	1.01	**1.02**	**1.02**		**1.61**		**0.96**

Supplementary Table 3: GAMLSS attenuation (ST1) coefficient estimates, stratified by contrast status (Cont). Bold entries indicate Bonferroni-adjusted $p < 0.05$. For μ (identity link), coefficients are additive effects on intensity; the kVp coefficient is scaled to represent the effect per 10 kVp. For σ (log link), coefficients are reported as $\exp(\beta)$ and represent multiplicative effects on the scale parameter. Age terms (Age-1 to Age-3) are fractional-polynomial basis coefficients from FP(Age). Manufacturer effects (GE/SIEMENS/TOSHIBA) are relative to the reference manufacturer level (Philips). Regression coefficients for all structures are reported in Supplementary Table 5.

		μ								σ		
Structure	Cont	Age-1	Age-2	Age-3	Sex	kVp×10	GE	SIE	TOS	Age-1	Age-2	Sex
aorta	×	**0**	**0**		0	**0**		**3**		**1.00**		**1.20**
aorta	✓	**-14**	**6**		**-18**	**-26**	-13	**-16**		**0.99**	**1.00**	**0.83**
gluteus minimus right	×	**-21**	**7**	**-3**	**3**	**-1**	**11**	**3**		**9.10**	**1.03**	**0.76**
gluteus minimus right	✓	**9**	**0**		**2**	**-3**	**12**	0		**1.01**	**1.00**	**0.84**
heart	×	**0**	**0**		**2**	0		**0**		**239.76**	**0.03**	**1.08**
heart	✓	**-1**	**1**		**-15**	**-24**	**-11**	**-15**	**-29**	**0.99**	**1.00**	**0.84**
kidney right	×	**-25**	**85**		**-1**	0	5	1		**1.01**	**0.99**	**1.42**
kidney right	✓	**-15**			**-16**	**-13**	**-13**	**-23**		**1.00**		**0.92**
liver	×	-10	**55**		**-2**	**1**	5	**3**		**1.03**	**1.00**	1.04
liver	✓	**35**			**-9**	**-5**		**-6**	4	**1.00**		**0.92**
lung upper lobe right	×	**54**			**-7**	**-3**		**-5**				0.98
lung upper lobe right	✓	**0**	**0**		**-14**	**-4**	**-23**	**11**	-7	**0.99**	**1.00**	1.00
spleen	×	**0**	**0**		**1**	0	**6**	**2**		**1.01**	**1.00**	**1.14**
spleen	✓	**17**			**-8**	**-7**	**-10**	**-20**	**-8**	**1.00**		**0.90**
vertebrae L3	×	**-2**	**1**		**-15**	-1	-19	**-54**		**1.01**		**0.90**
vertebrae L3	✓	**202**	**-149**	**16**	**-9**	**-23**	**-34**	**-48**		**1.00**		0.98

Reference charts for CT volume

Supplementary Figure 7: Extension of Figure 3 from the main paper with plots for all anatomical structures.

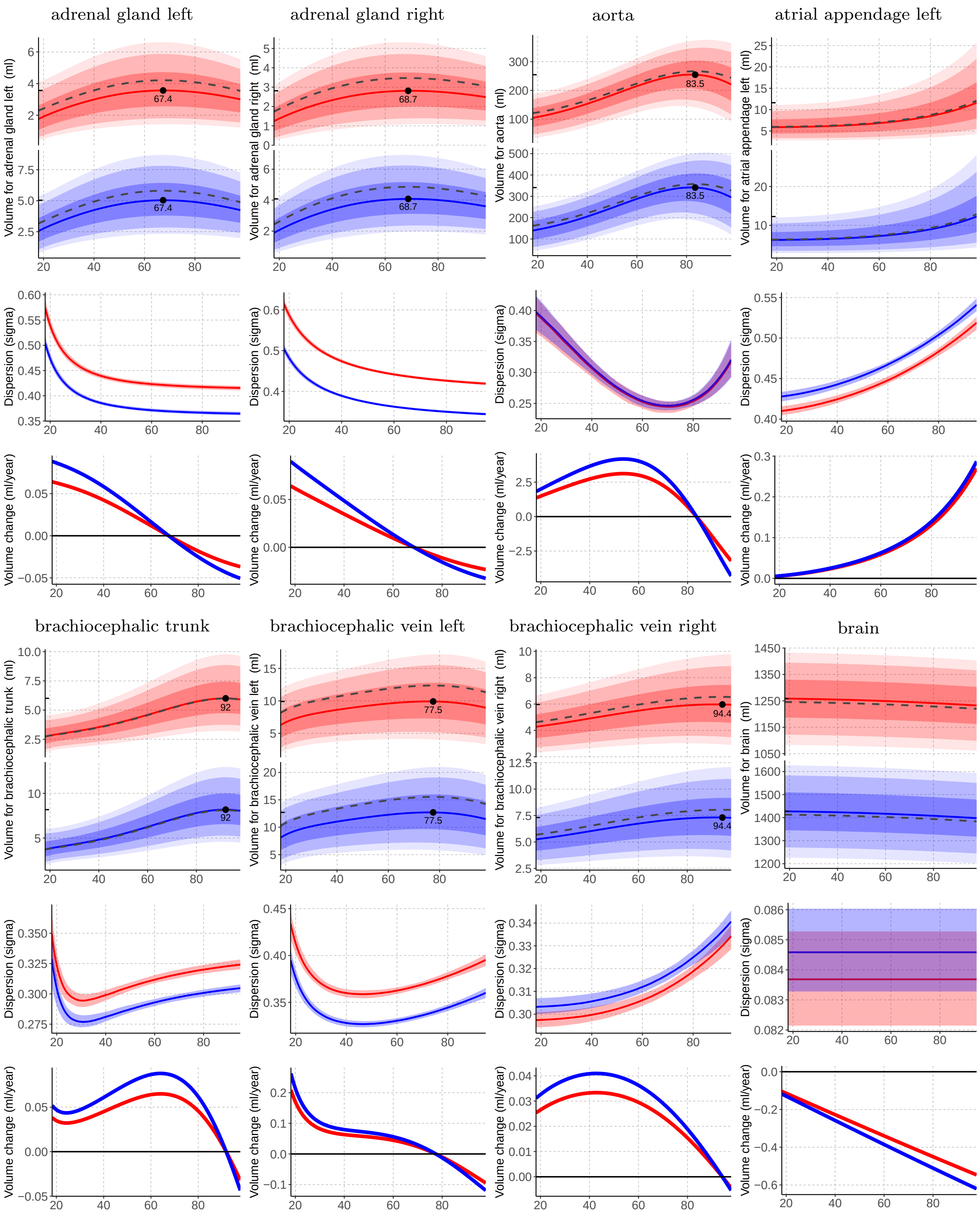

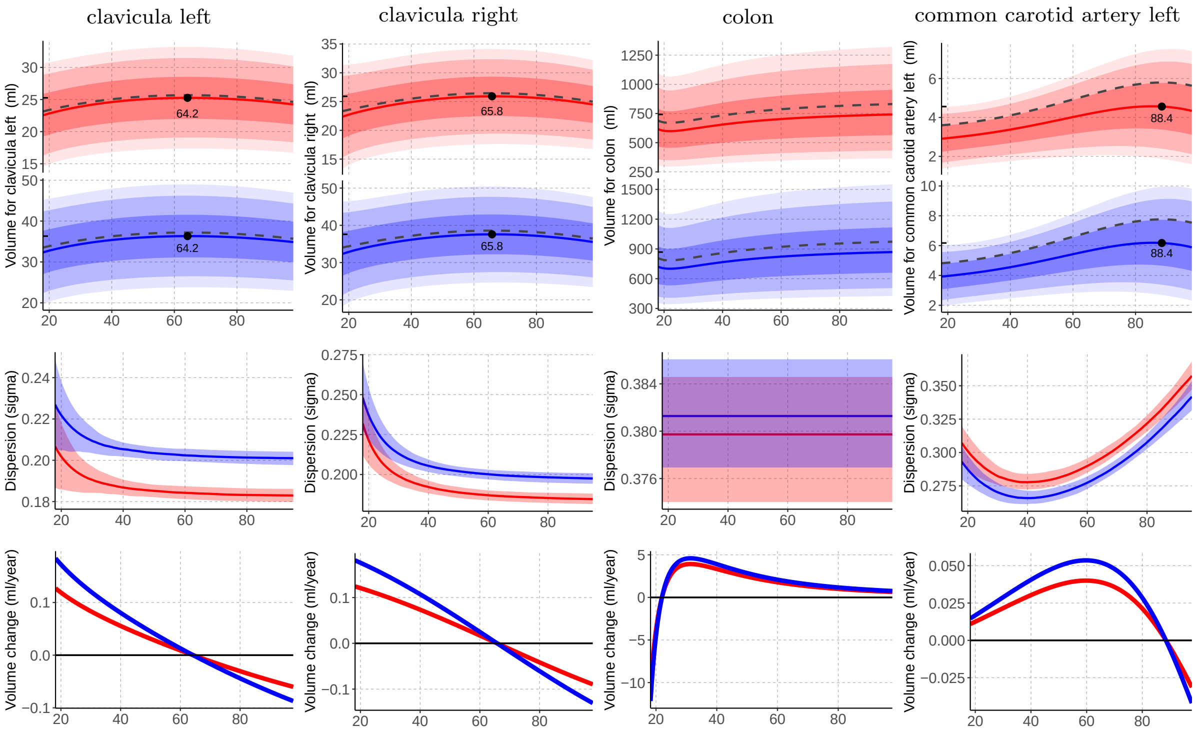
clavicula left
clavicula right
colon
common carotid artery left
Volume for clavicula left (ml)
Volume for clavicula right (ml)
Volume for colon (ml)
Volume for common carotid artery left (ml)
Dispersion (sigma)
Volume change (ml/year)
64.2
65.8
88.4

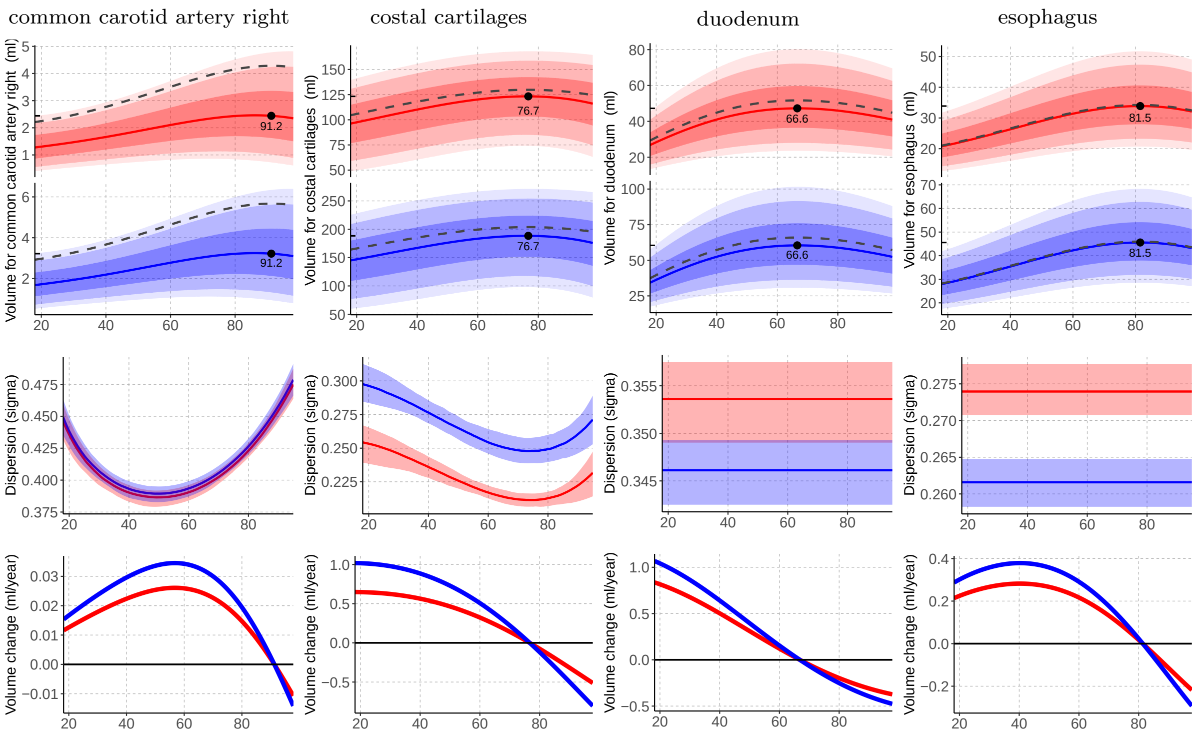
common carotid artery right
costal cartilages
duodenum
esophagus
Volume for common carotid artery right (ml)
Volume for costal cartilages (ml)
Volume for duodenum (ml)
Volume for esophagus (ml)
Dispersion (sigma)
Volume change (ml/year)
91.2
76.7
66.6
81.5

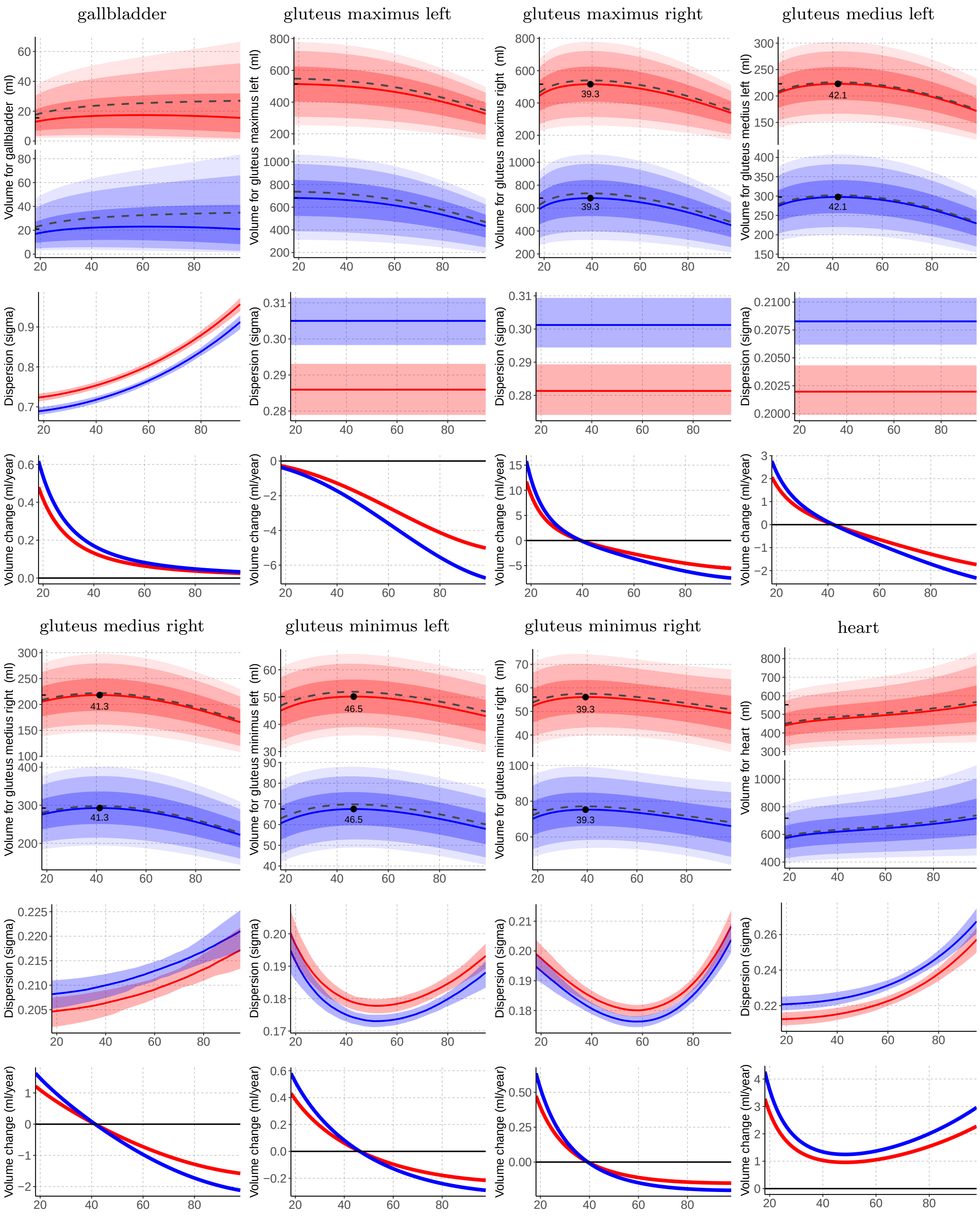
gallbladder
gluteus maximus left
gluteus maximus right
gluteus medius left
Volume for gallbladder (ml)
Volume for gluteus maximus left (ml)
Volume for gluteus maximus right (ml)
Volume for gluteus medius left (ml)
39.3
42.1
Dispersion (sigma)
Volume change (ml/year)
gluteus medius right
gluteus minimus left
gluteus minimus right
heart
Volume for gluteus medius right (ml)
Volume for gluteus minimus left (ml)
Volume for gluteus minimus right (ml)
Volume for heart (ml)
41.3
46.5
39.3
Dispersion (sigma)
Volume change (ml/year)

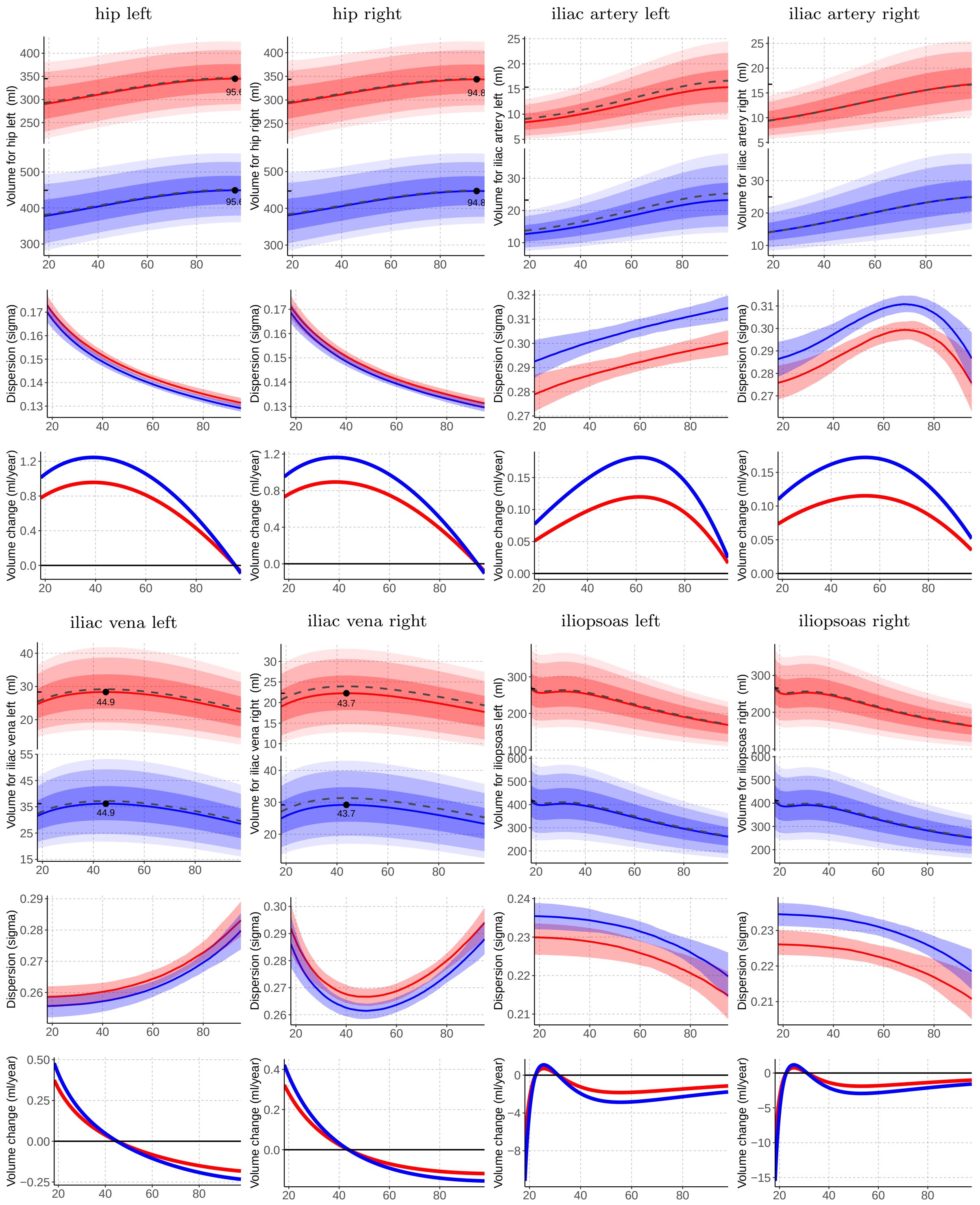
hip left
hip right
iliac artery left
iliac artery right
Volume for hip left (ml)
Volume for hip right (ml)
Volume for iliac artery left (ml)
Volume for iliac artery right (ml)
95.6
94.8
Dispersion (sigma)
Volume change (ml/year)
iliac vena left
iliac vena right
iliopsoas left
iliopsoas right
Volume for iliac vena left (ml)
Volume for iliac vena right (ml)
Volume for iliopsoas left (ml)
Volume for iliopsoas right (ml)
44.9
43.7
Dispersion (sigma)
Volume change (ml/year)
20
40
60
80

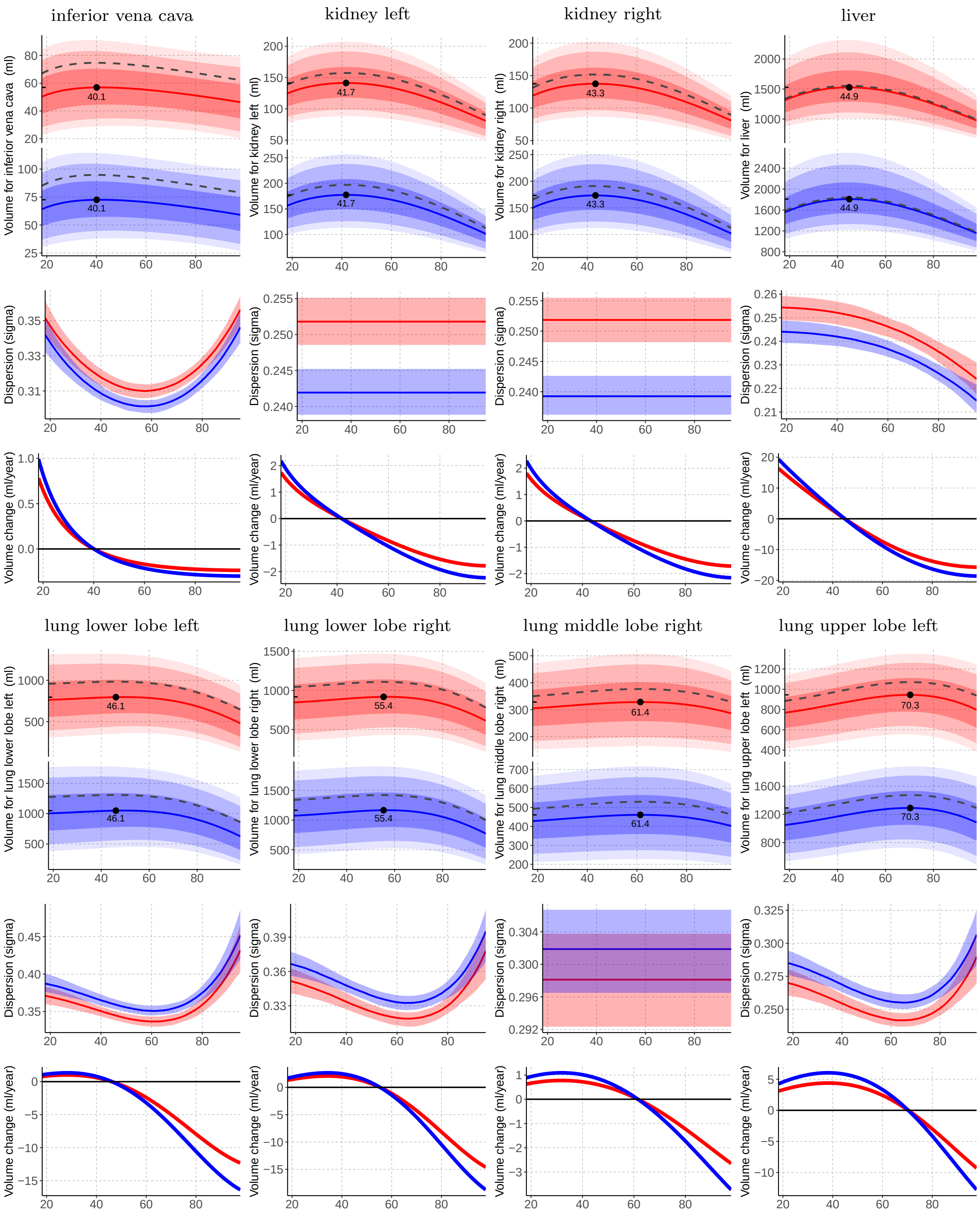

inferior vena cava
kidney left
kidney right
liver
Volume for inferior vena cava (ml)
Volume for kidney left (ml)
Volume for kidney right (ml)
Volume for liver (ml)
40.1
41.7
43.3
44.9
Dispersion (sigma)
Volume change (ml/year)
lung lower lobe left
lung lower lobe right
lung middle lobe right
lung upper lobe left
Volume for lung lower lobe left (ml)
Volume for lung lower lobe right (ml)
Volume for lung middle lobe right (ml)
Volume for lung upper lobe left (ml)
46.1
55.4
61.4
70.3
Dispersion (sigma)
Volume change (ml/year)

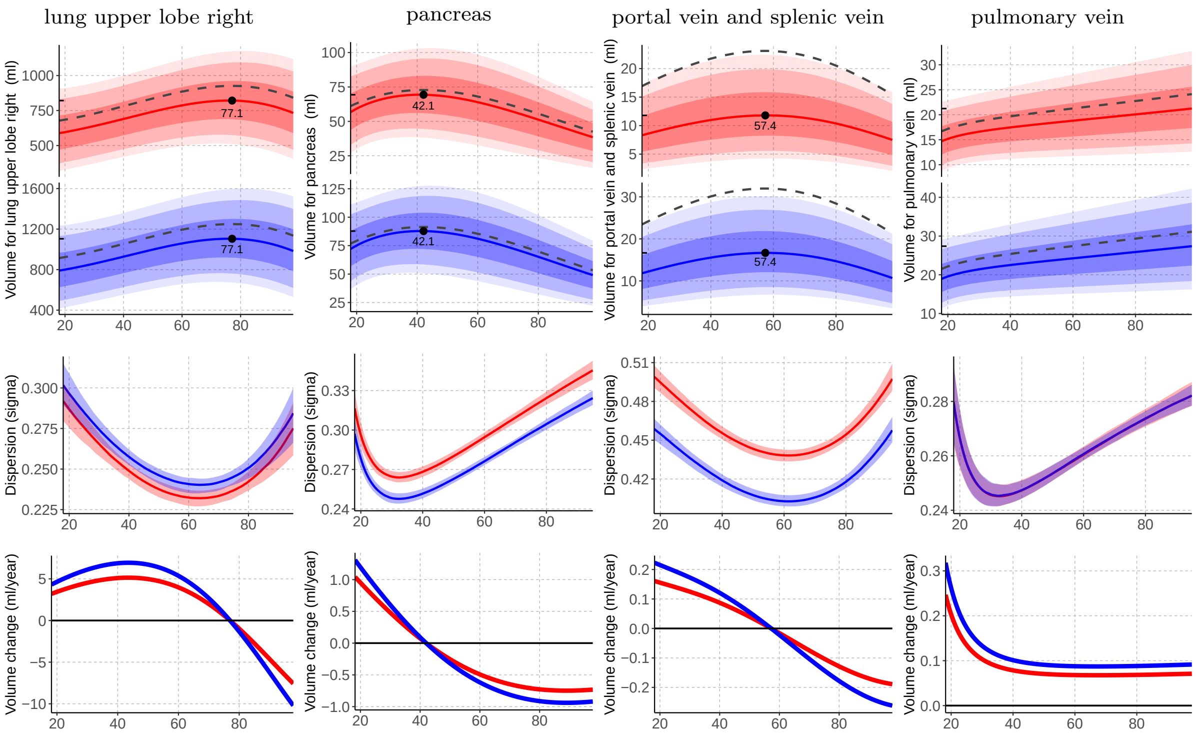
lung upper lobe right
pancreas
portal vein and splenic vein
pulmonary vein
Volume for lung upper lobe right (ml)
Volume for pancreas (ml)
Volume for portal vein and splenic vein (ml)
Volume for pulmonary vein (ml)
77.1
42.1
57.4
Dispersion (sigma)
Volume change (ml/year)

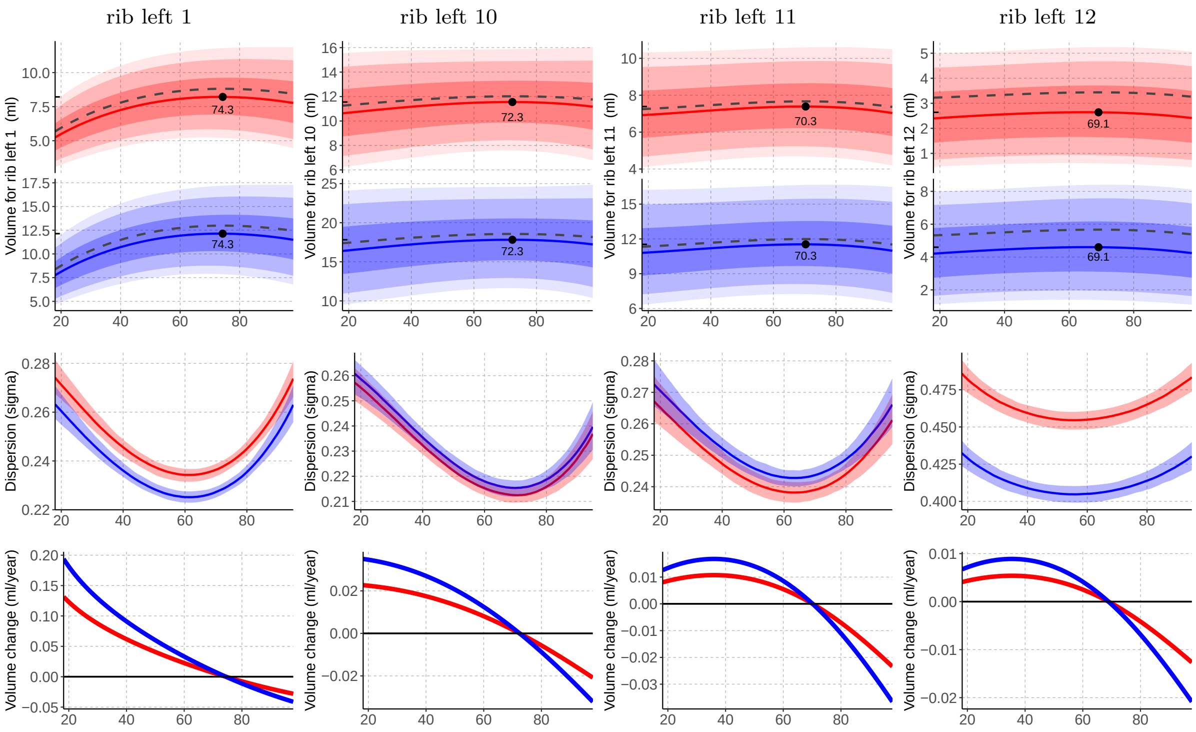
rib left 1
rib left 10
rib left 11
rib left 12
Volume for rib left 1 (ml)
Volume for rib left 10 (ml)
Volume for rib left 11 (ml)
Volume for rib left 12 (ml)
74.3
72.3
70.3
69.1
Dispersion (sigma)
Volume change (ml/year)

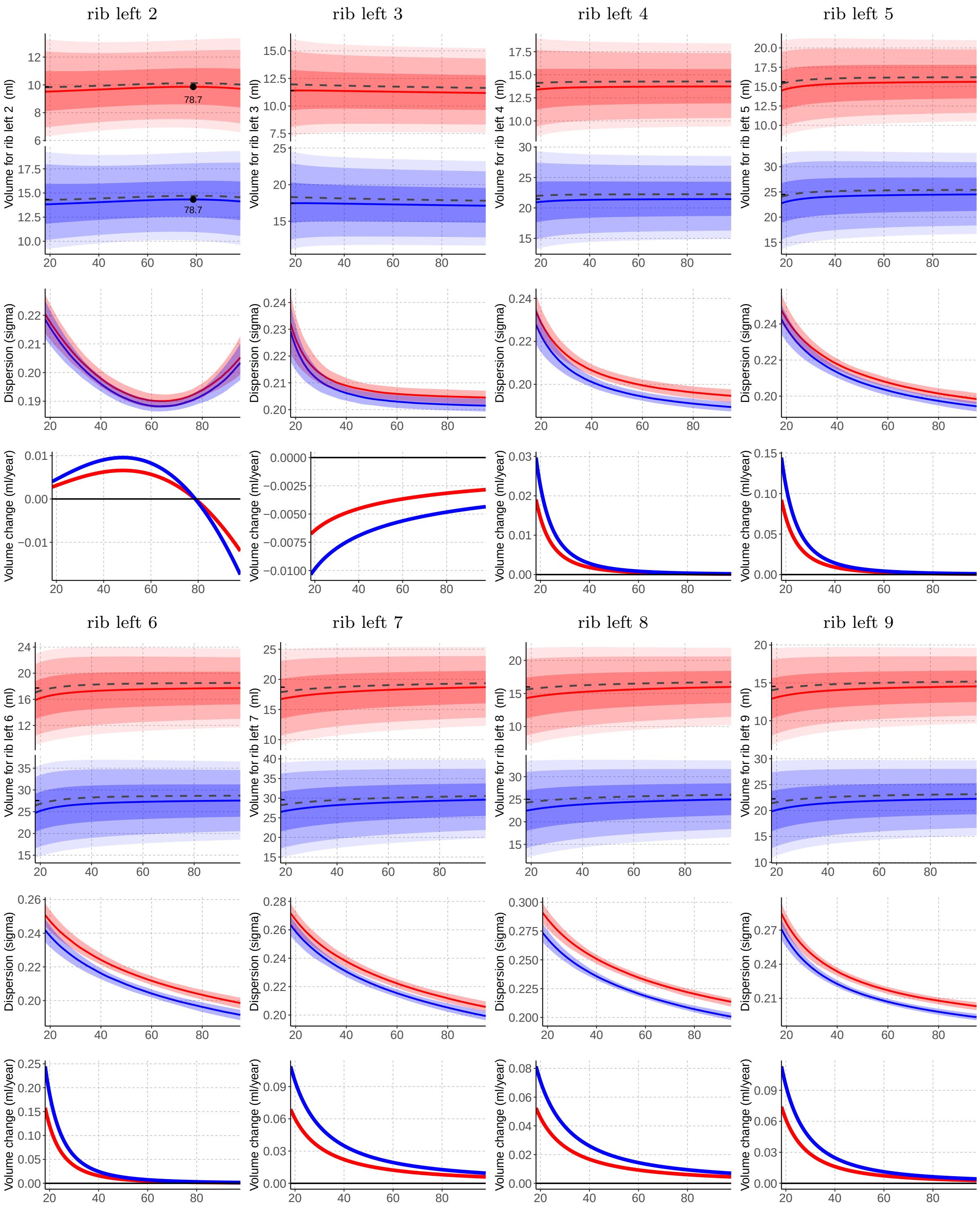
rib left 2
rib left 3
rib left 4
rib left 5
Volume for rib left 2 (ml)
Volume for rib left 3 (ml)
Volume for rib left 4 (ml)
Volume for rib left 5 (ml)
78.7
78.7
Dispersion (sigma)
Volume change (ml/year)
rib left 6
rib left 7
rib left 8
rib left 9
Volume for rib left 6 (ml)
Volume for rib left 7 (ml)
Volume for rib left 8 (ml)
Volume for rib left 9 (ml)
Dispersion (sigma)
Volume change (ml/year)

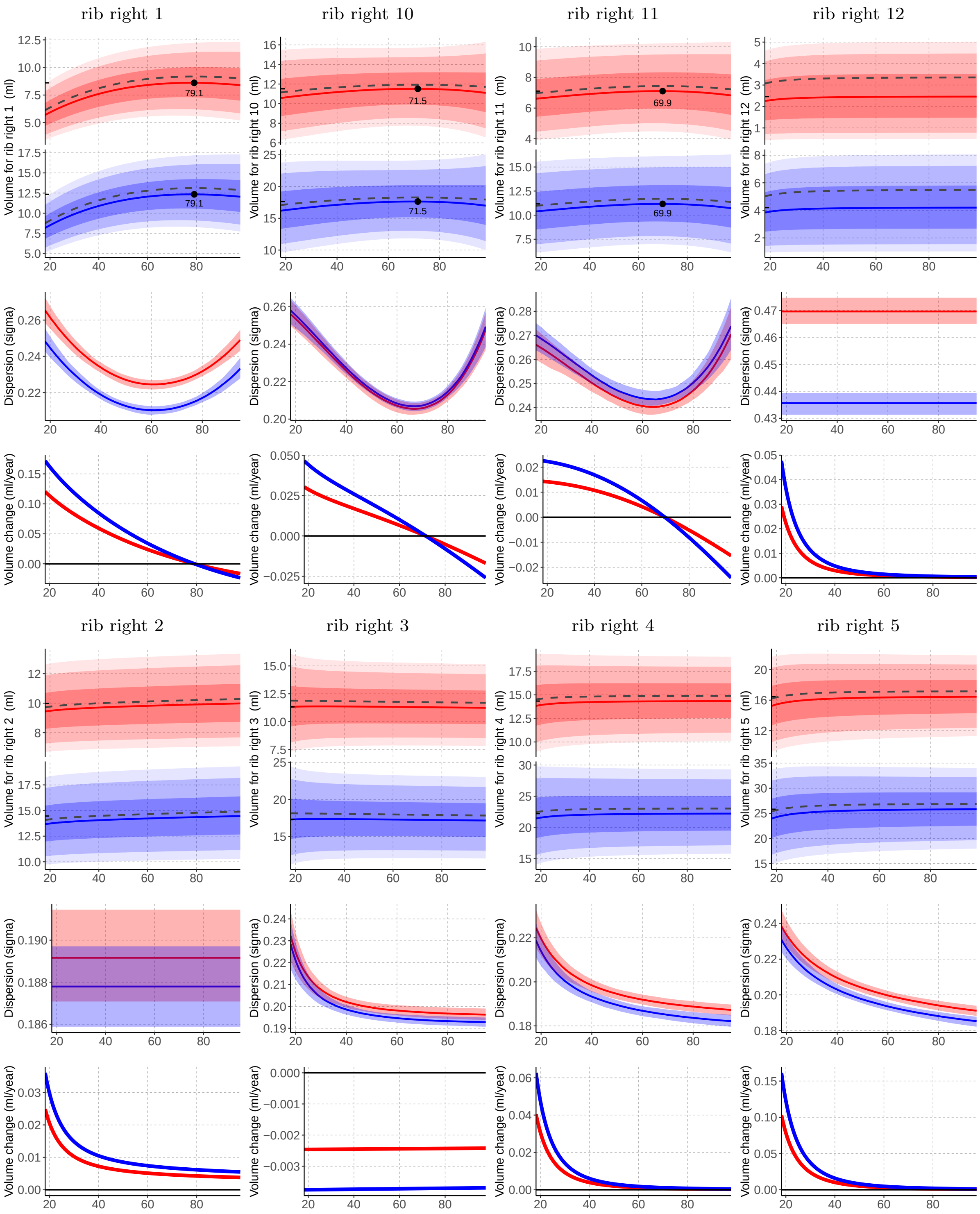
rib right 1
rib right 10
rib right 11
rib right 12
Volume for rib right 1 (ml)
Volume for rib right 10 (ml)
Volume for rib right 11 (ml)
Volume for rib right 12 (ml)
79.1
71.5
69.9
Dispersion (sigma)
Volume change (ml/year)
rib right 2
rib right 3
rib right 4
rib right 5
Volume for rib right 2 (ml)
Volume for rib right 3 (ml)
Volume for rib right 4 (ml)
Volume for rib right 5 (ml)

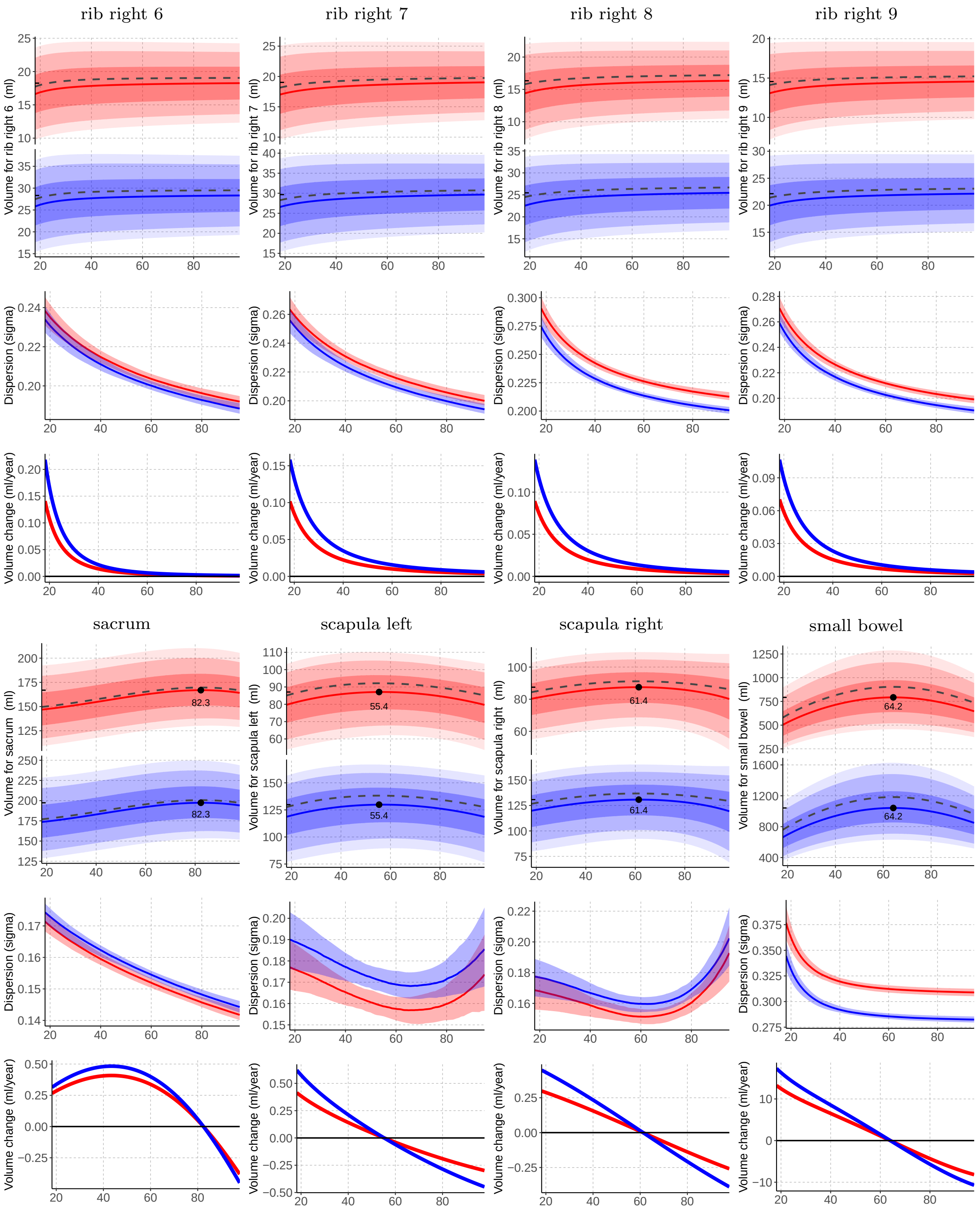

rib right 6
rib right 7
rib right 8
rib right 9
Volume for rib right 6 (ml)
Volume for rib right 7 (ml)
Volume for rib right 8 (ml)
Volume for rib right 9 (ml)
Dispersion (sigma)
Volume change (ml/year)
sacrum
scapula left
scapula right
small bowel
Volume for sacrum (ml)
Volume for scapula left (ml)
Volume for scapula right (ml)
Volume for small bowel (ml)
82.3
55.4
61.4
64.2

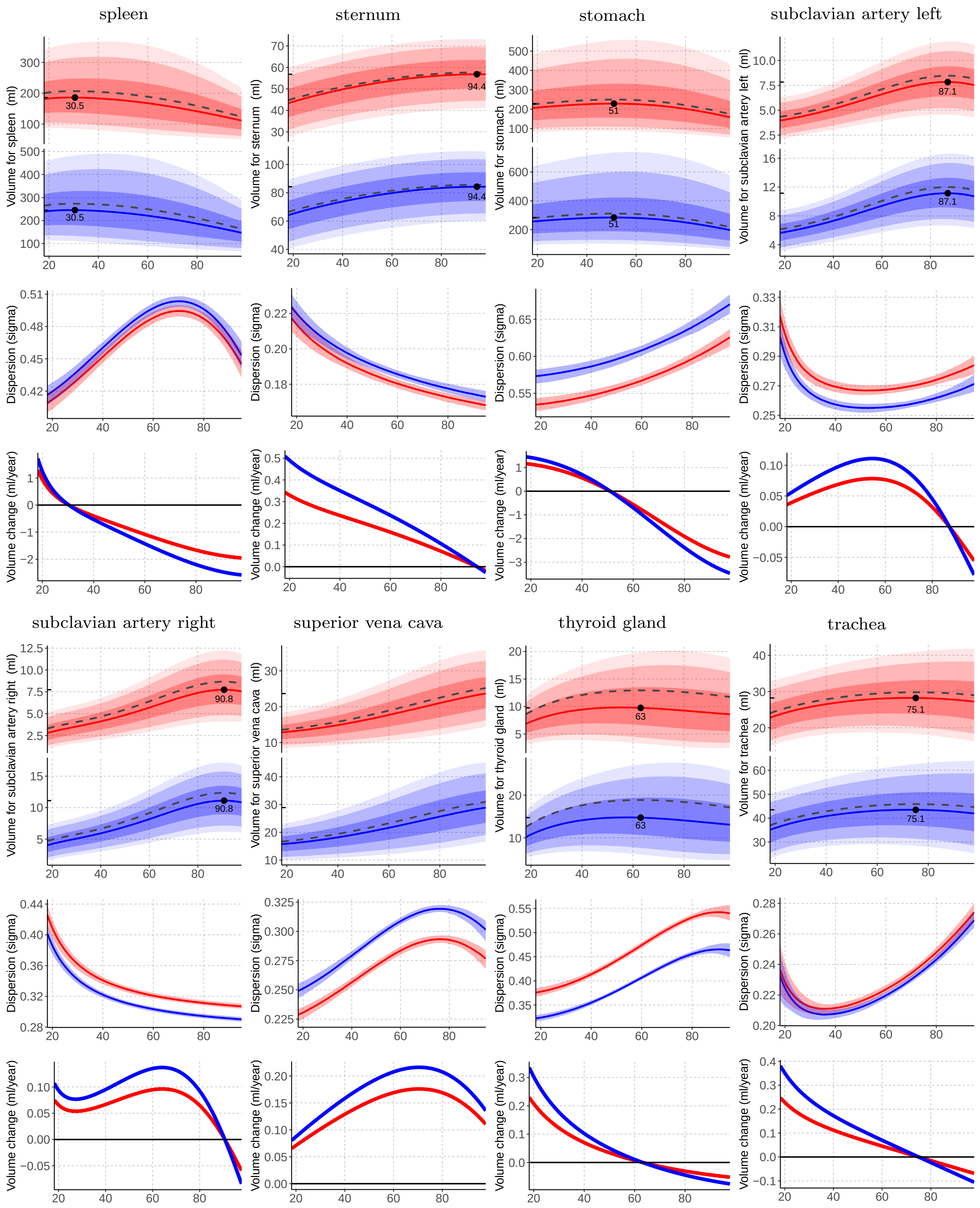
spleen
sternum
stomach
subclavian artery left
Volume for spleen (ml)
Volume for sternum (ml)
Volume for stomach (ml)
Volume for subclavian artery left (ml)
Dispersion (sigma)
Volume change (ml/year)
subclavian artery right
superior vena cava
thyroid gland
trachea
Volume for subclavian artery right (ml)
Volume for superior vena cava (ml)
Volume for thyroid gland (ml)
Volume for trachea (ml)
Dispersion (sigma)
Volume change (ml/year)

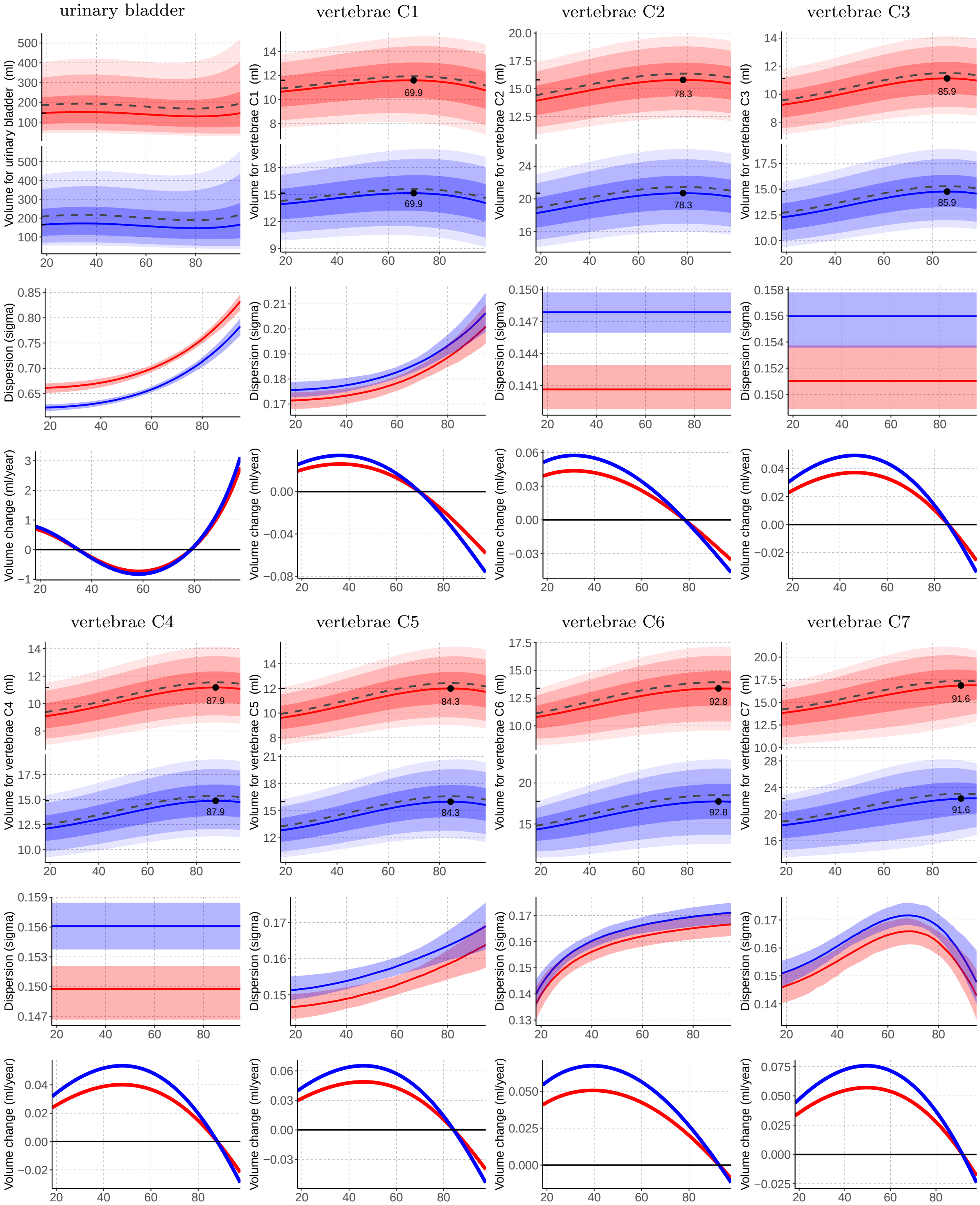
urinary bladder
vertebrae C1
vertebrae C2
vertebrae C3
vertebrae C4
vertebrae C5
vertebrae C6
vertebrae C7
Volume for urinary bladder (ml)
Dispersion (sigma)
Volume change (ml/year)
69.9
78.3
85.9
87.9
84.3
92.8
91.6

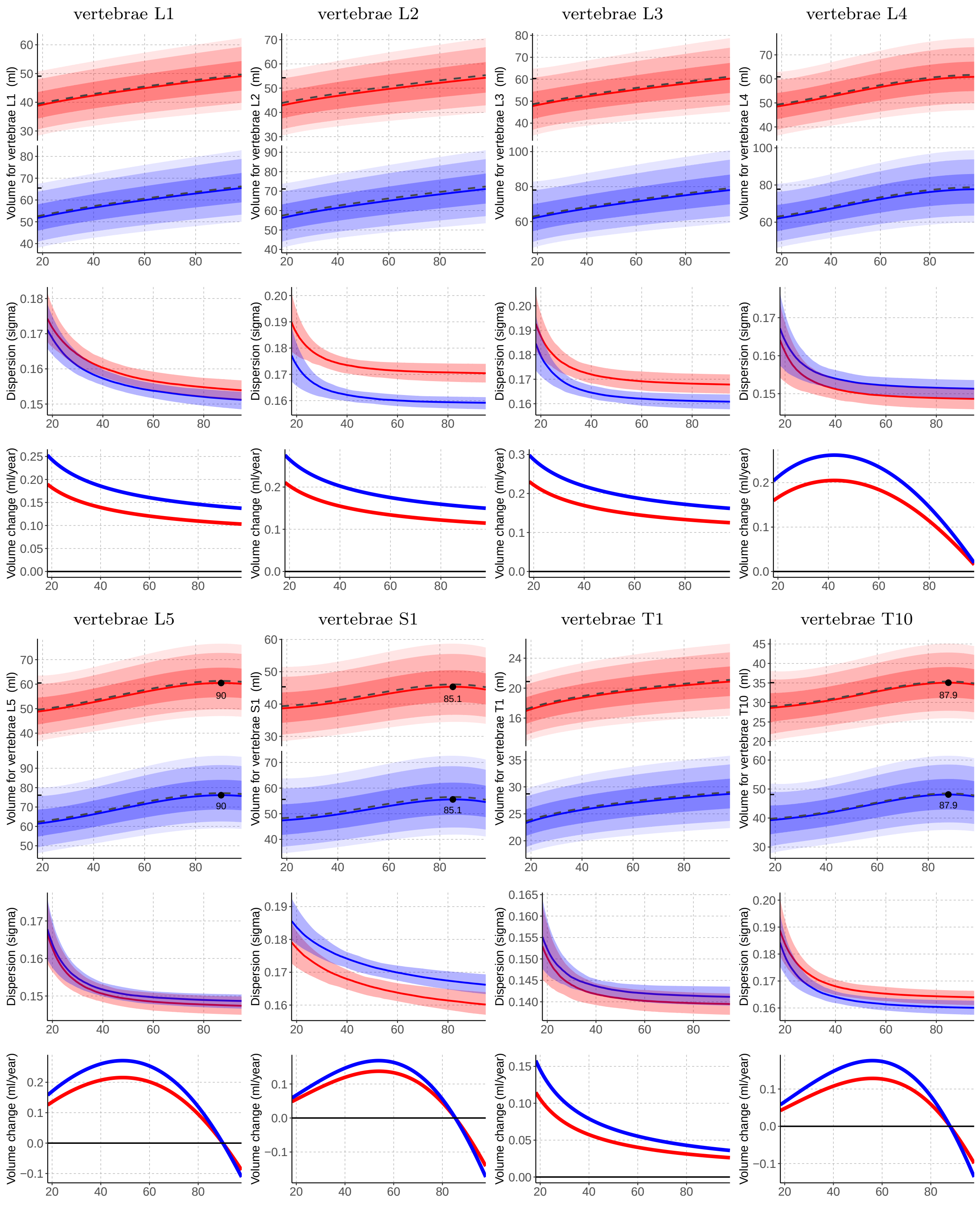
vertebrae L1
vertebrae L2
vertebrae L3
vertebrae L4
Volume for vertebrae L1 (ml)
Volume for vertebrae L2 (ml)
Volume for vertebrae L3 (ml)
Volume for vertebrae L4 (ml)
Dispersion (sigma)
Volume change (ml/year)
vertebrae L5
vertebrae S1
vertebrae T1
vertebrae T10
Volume for vertebrae L5 (ml)
Volume for vertebrae S1 (ml)
Volume for vertebrae T1 (ml)
Volume for vertebrae T10 (ml)
90
85.1
87.9

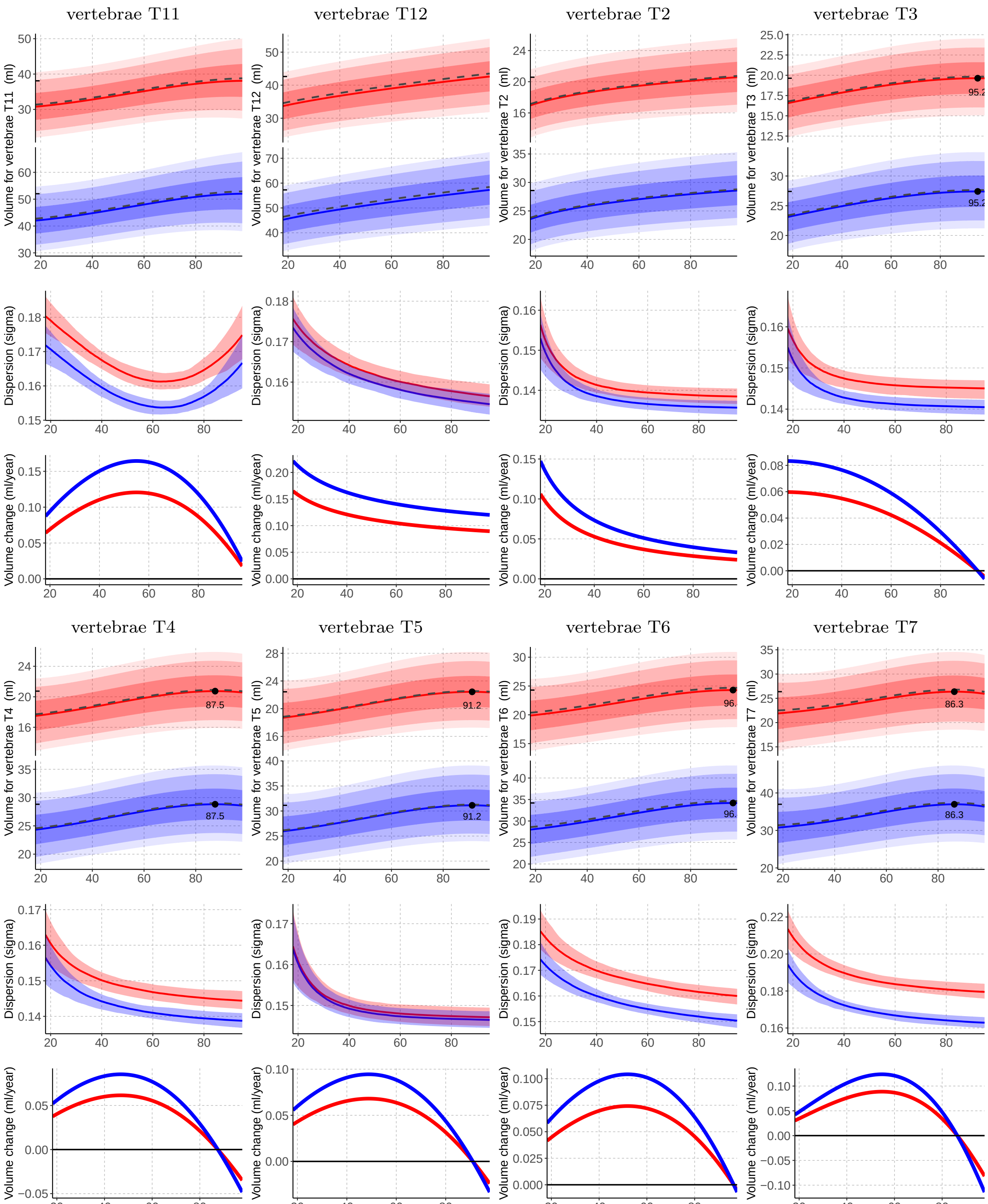
vertebrae T11
vertebrae T12
vertebrae T2
vertebrae T3
Volume for vertebrae T11 (ml)
Volume for vertebrae T12 (ml)
Volume for vertebrae T2 (ml)
Volume for vertebrae T3 (ml)
95.2
Dispersion (sigma)
Volume change (ml/year)
vertebrae T4
vertebrae T5
vertebrae T6
vertebrae T7
Volume for vertebrae T4 (ml)
Volume for vertebrae T5 (ml)
Volume for vertebrae T6 (ml)
Volume for vertebrae T7 (ml)
87.5
91.2
96.
86.3
Dispersion (sigma)
Volume change (ml/year)

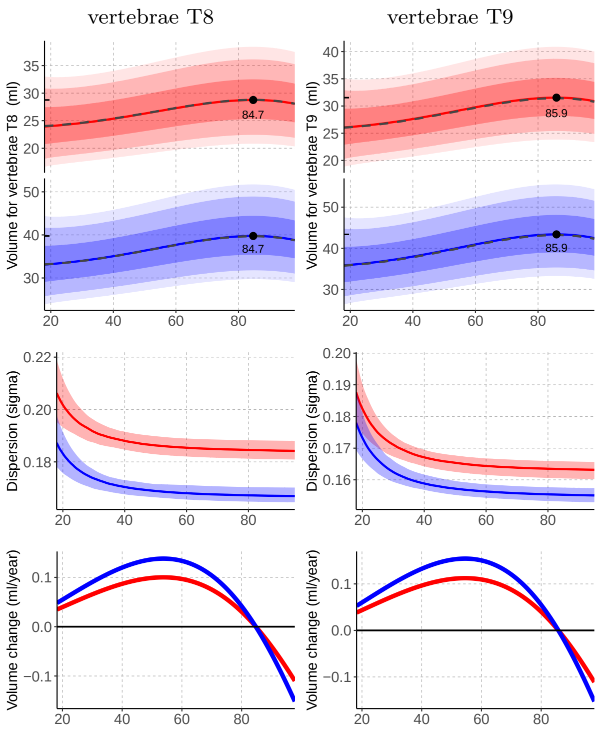
vertebrae T8
vertebrae T9
Volume for vertebrae T8 (ml)
Volume for vertebrae T9 (ml)
84.7
85.9
Dispersion (sigma)
Volume change (ml/year)

Reference charts for CT attenuation

Supplementary Figure 8: Extension of Figure 4 from the main paper with plots for all anatomical structures.

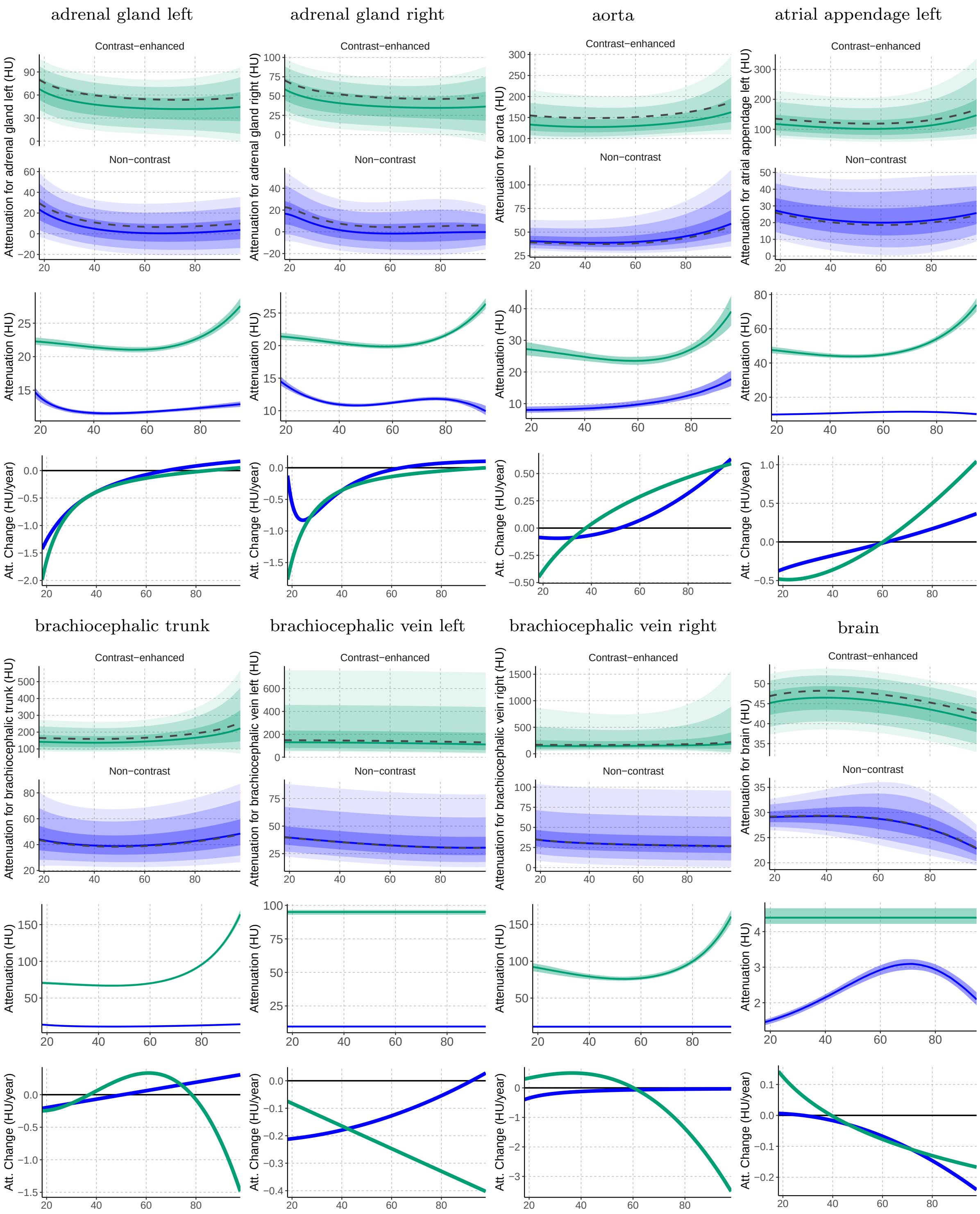

clavicula left

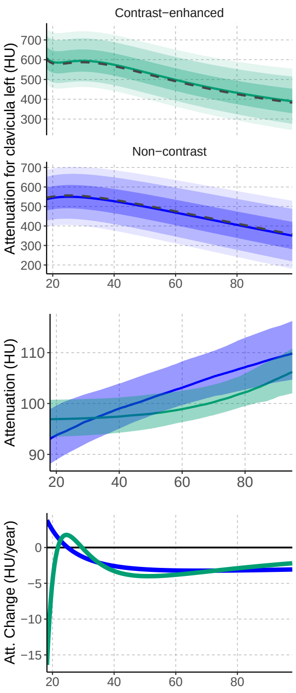

clavicula right

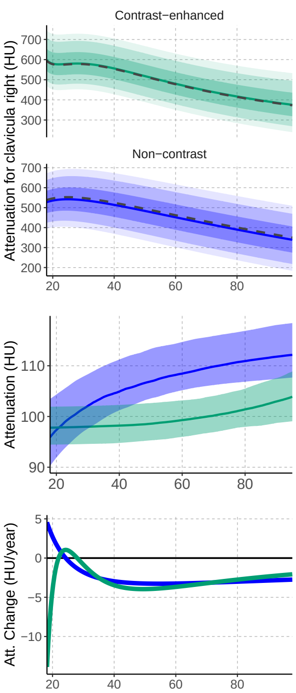

colon

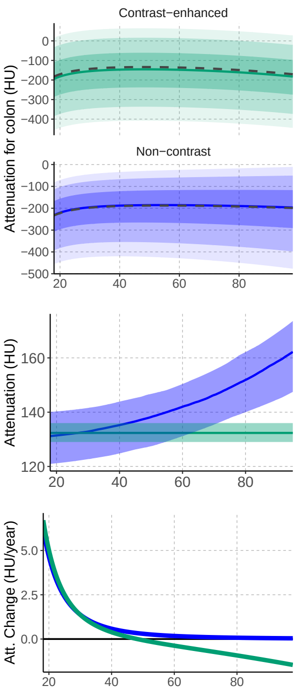

common carotid artery left

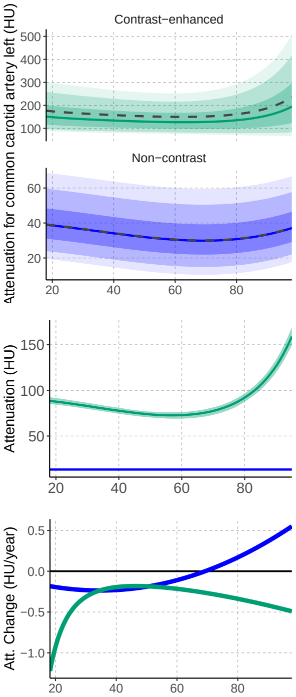

common carotid artery right

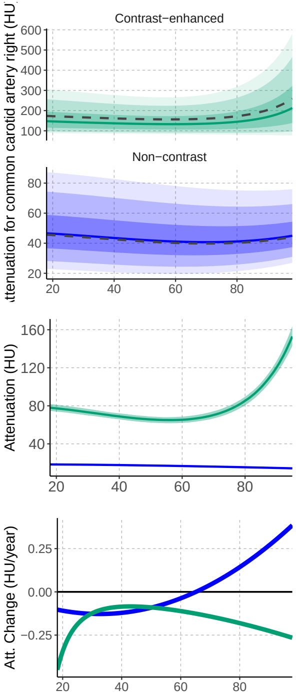

costal cartilages

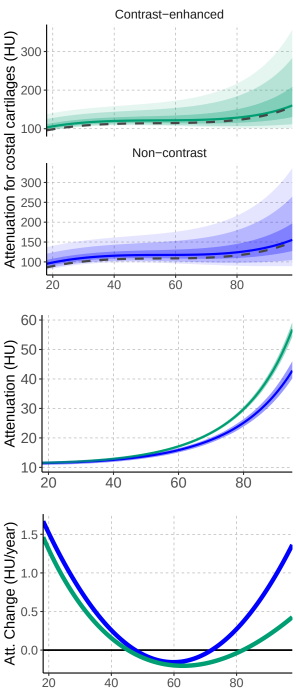

duodenum

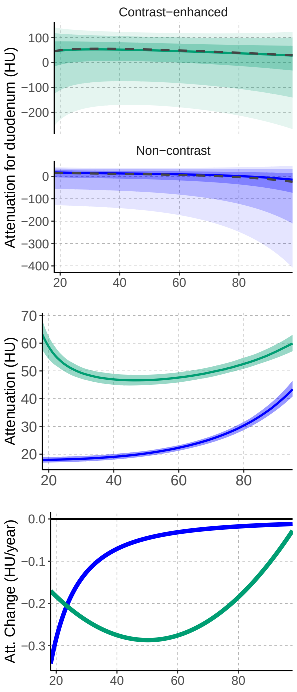

esophagus

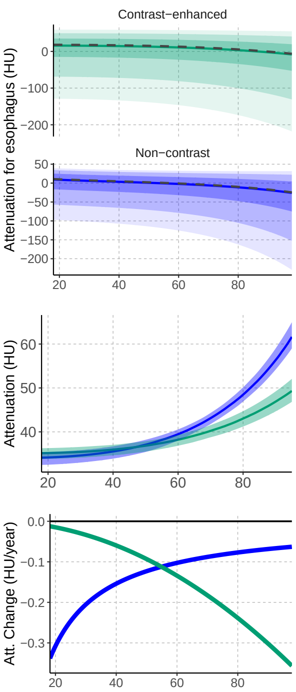

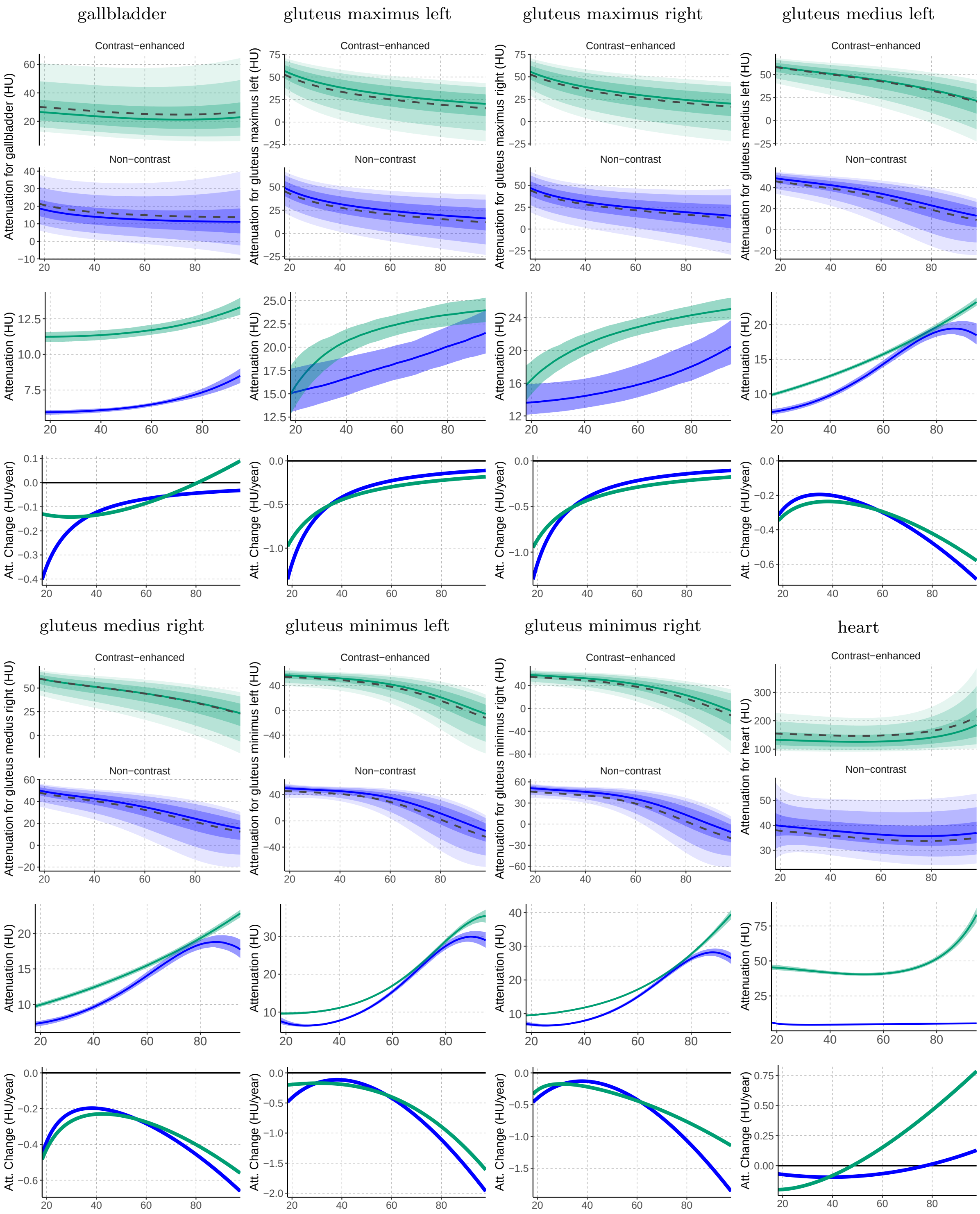
gallbladder
gluteus maximus left
gluteus maximus right
gluteus medius left
Contrast−enhanced
Non−contrast
Attenuation for gallbladder (HU)
Attenuation for gluteus maximus left (HU)
Attenuation for gluteus maximus right (HU)
Attenuation for gluteus medius left (HU)
Attenuation (HU)
Att. Change (HU/year)
gluteus medius right
gluteus minimus left
gluteus minimus right
heart
Contrast−enhanced
Non−contrast
Attenuation for gluteus medius right (HU)
Attenuation for gluteus minimus left (HU)
Attenuation for gluteus minimus right (HU)
Attenuation for heart (HU)
Attenuation (HU)
Att. Change (HU/year)

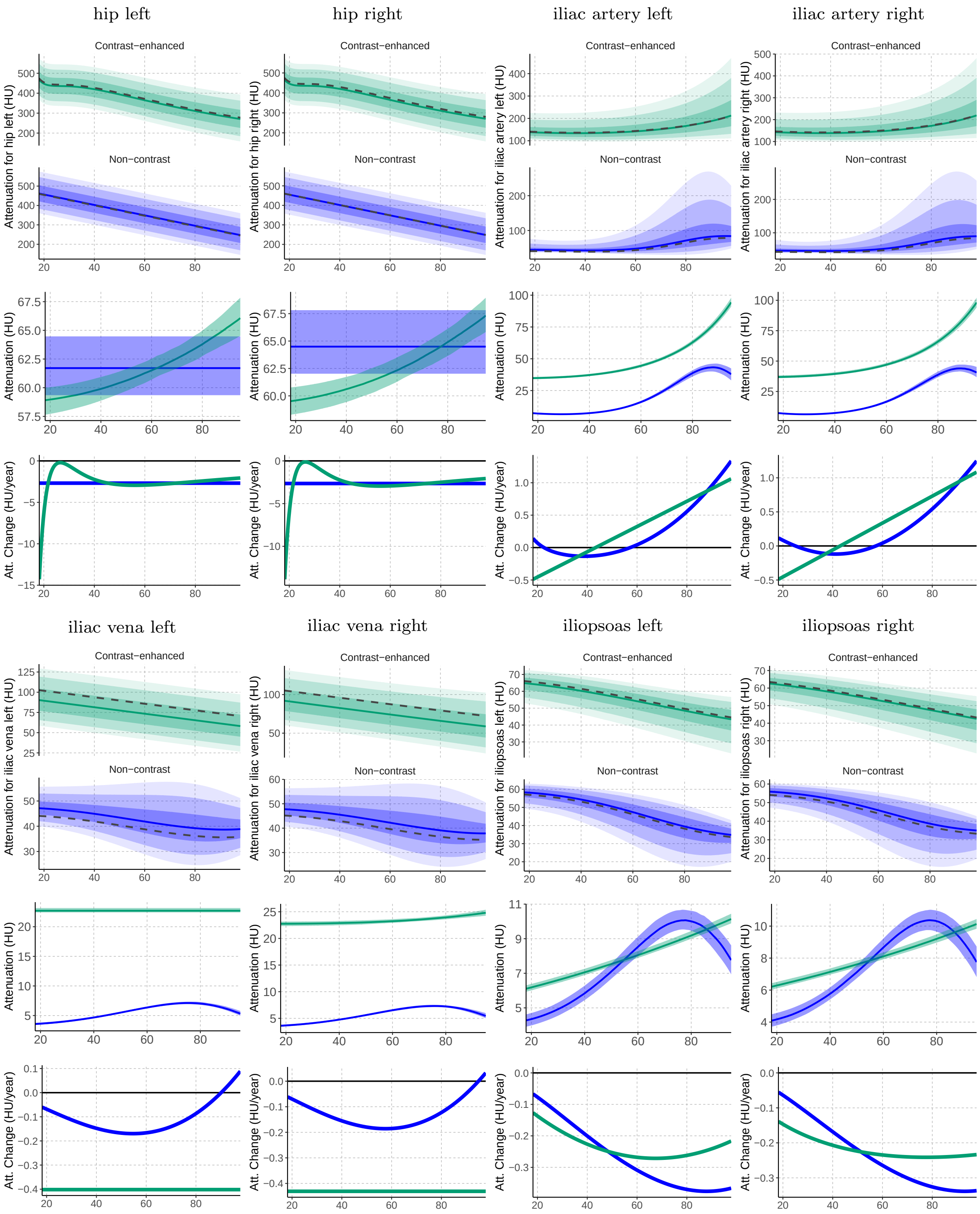
hip left
hip right
iliac artery left
iliac artery right
Contrast−enhanced
Non−contrast
Attenuation for hip left (HU)
Attenuation for hip right (HU)
Attenuation for iliac artery left (HU)
Attenuation for iliac artery right (HU)
Attenuation (HU)
Att. Change (HU/year)
iliac vena left
iliac vena right
iliopsoas left
iliopsoas right
Attenuation for iliac vena left (HU)
Attenuation for iliac vena right (HU)
Attenuation for iliopsoas left (HU)
Attenuation for iliopsoas right (HU)

inferior vena cava — kidney left — kidney right — liver

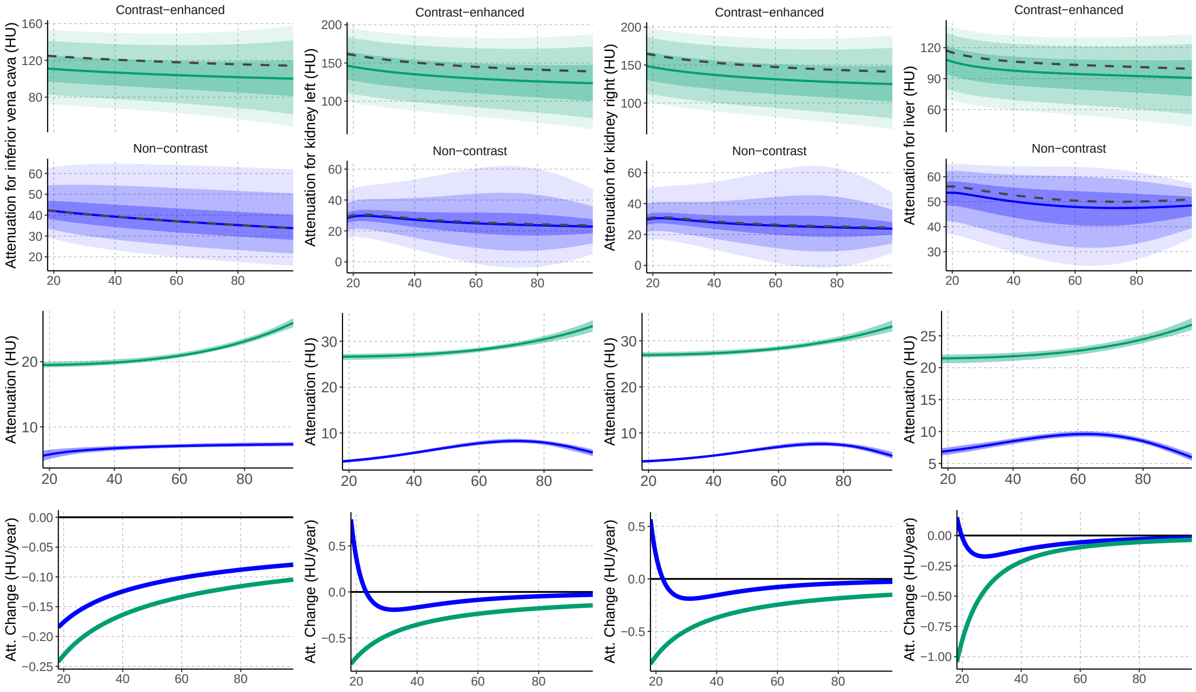

lung lower lobe left — lung lower lobe right — lung middle lobe right — lung upper lobe left

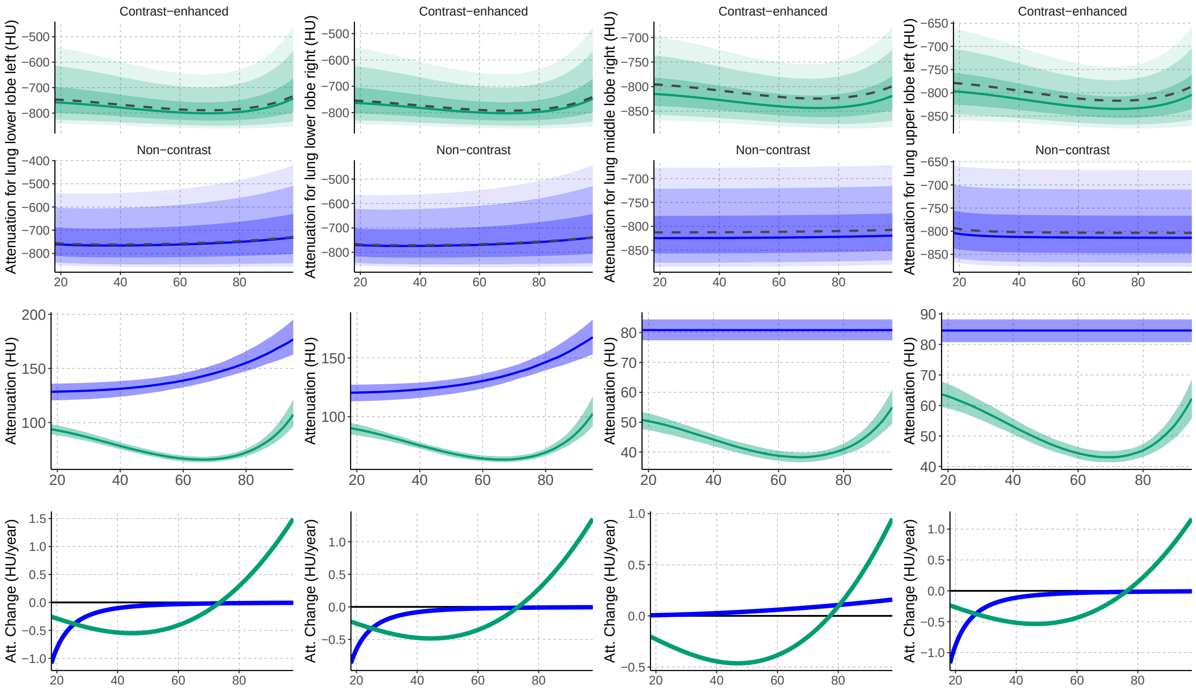

lung upper lobe right | pancreas | portal vein and splenic vein | pulmonary vein

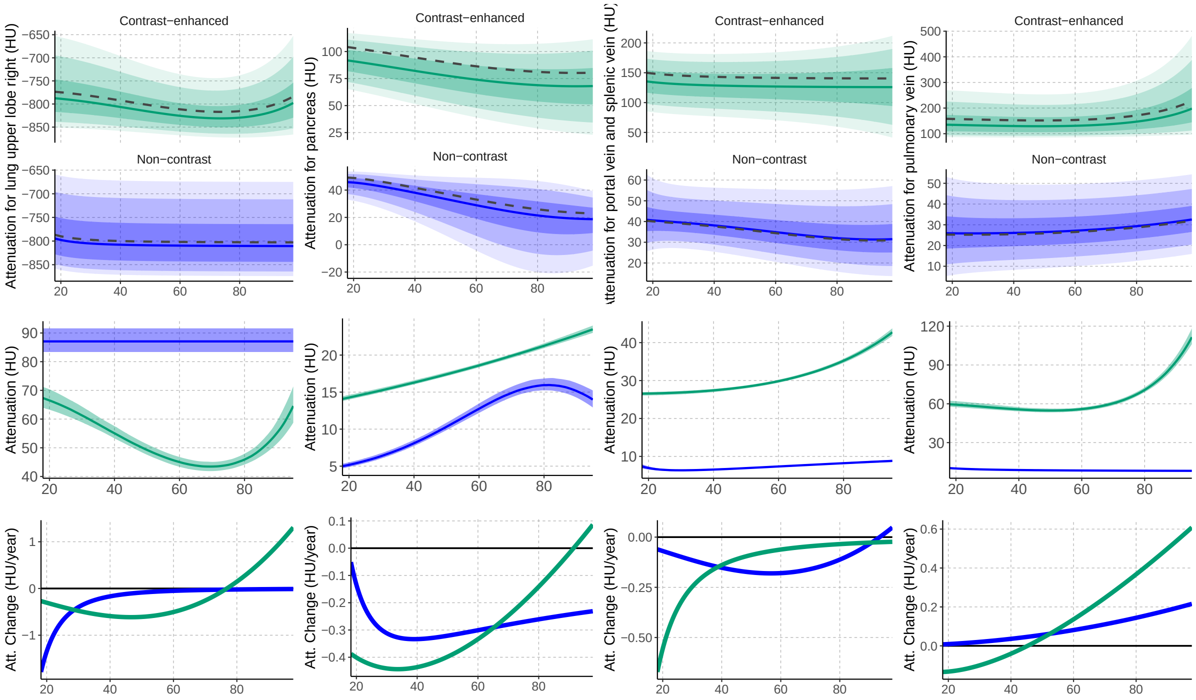

rib left 1 | rib left 10 | rib left 11 | rib left 12

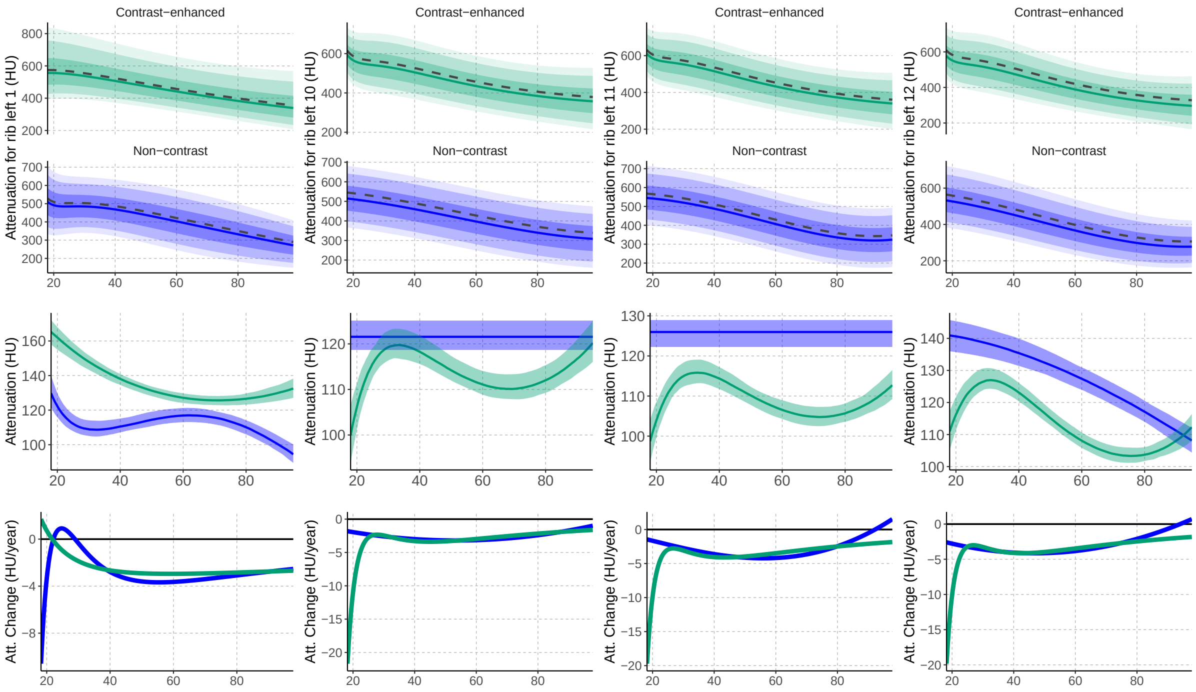

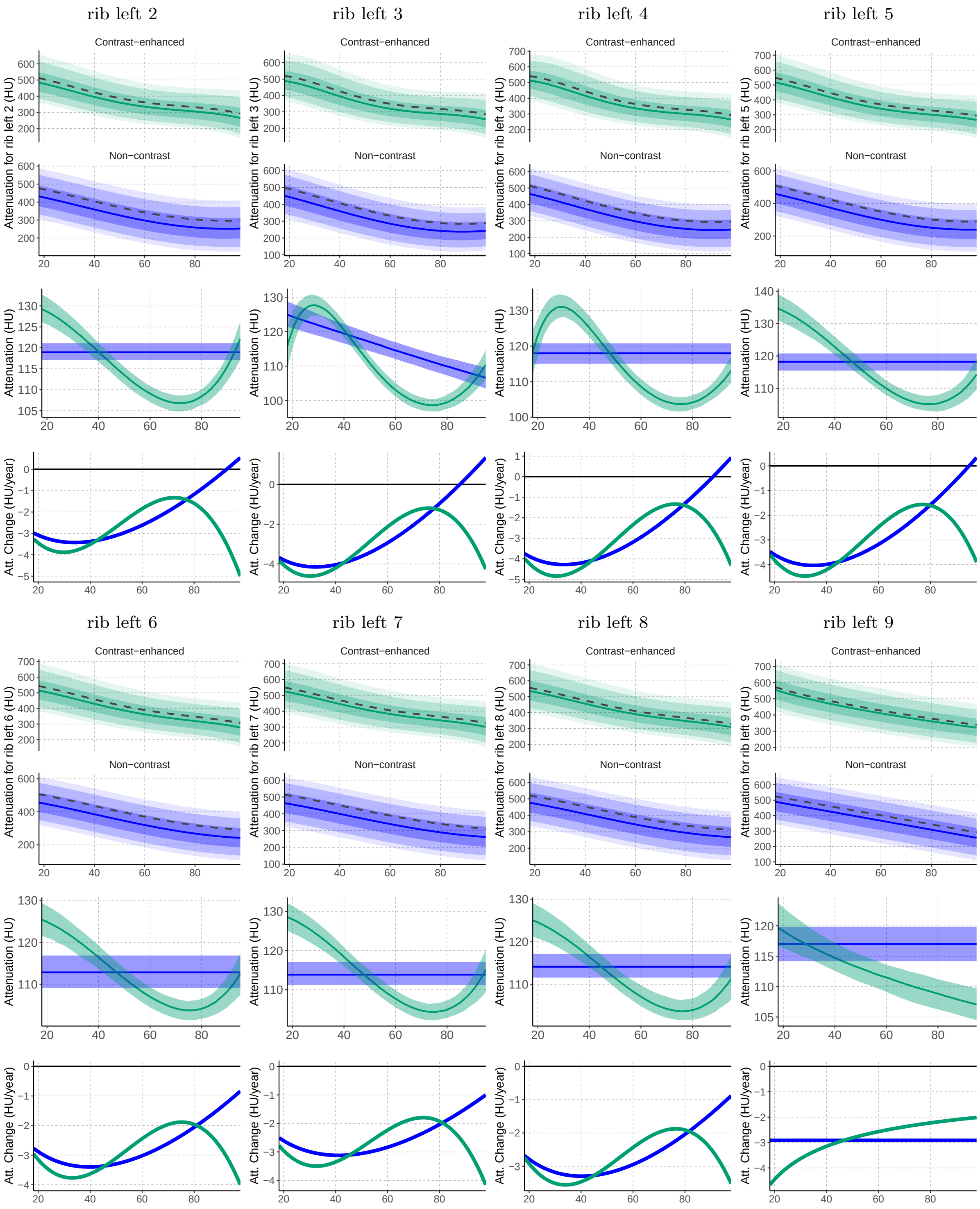
rib left 2
rib left 3
rib left 4
rib left 5
Contrast−enhanced
Non−contrast
Attenuation for rib left 2 (HU)
Attenuation for rib left 3 (HU)
Attenuation for rib left 4 (HU)
Attenuation for rib left 5 (HU)
Attenuation (HU)
Att. Change (HU/year)
rib left 6
rib left 7
rib left 8
rib left 9
Attenuation for rib left 6 (HU)
Attenuation for rib left 7 (HU)
Attenuation for rib left 8 (HU)
Attenuation for rib left 9 (HU)

rib right 1

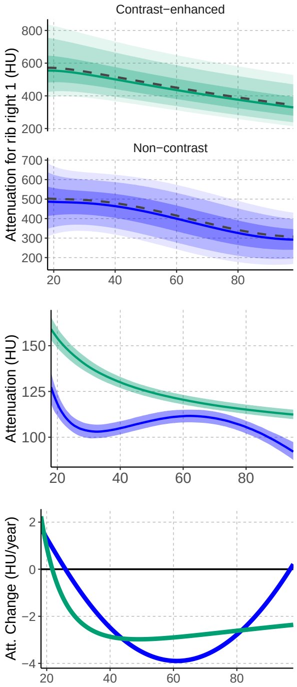

rib right 10

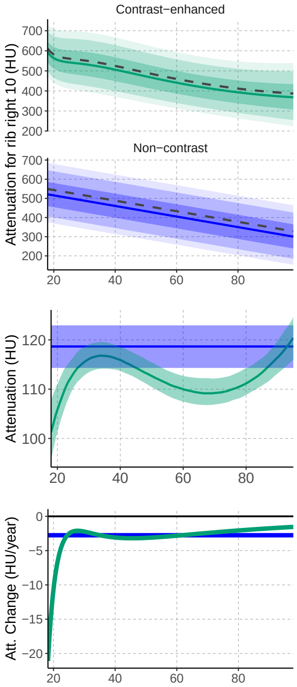

rib right 11

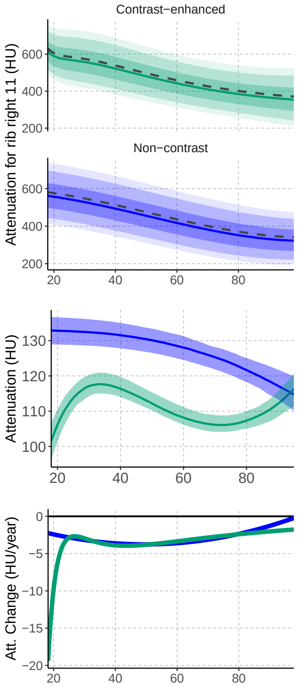

rib right 12

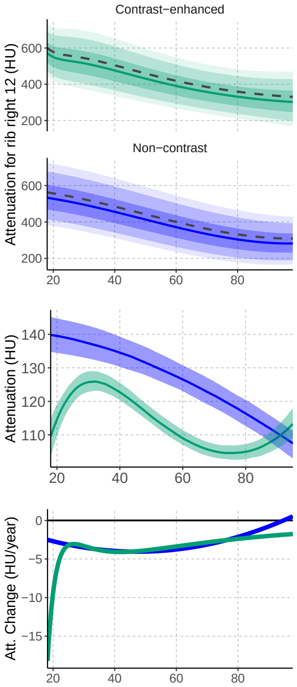

rib right 2

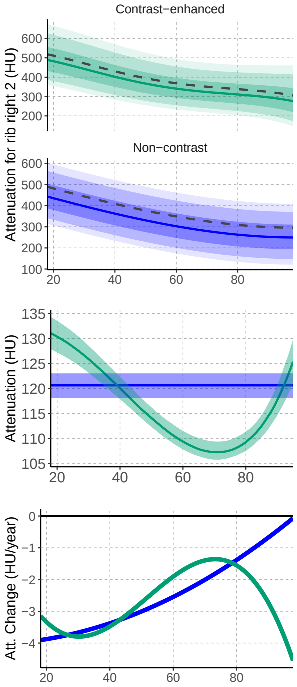

rib right 3

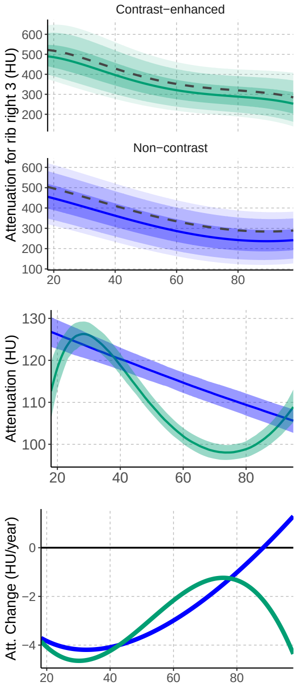

rib right 4

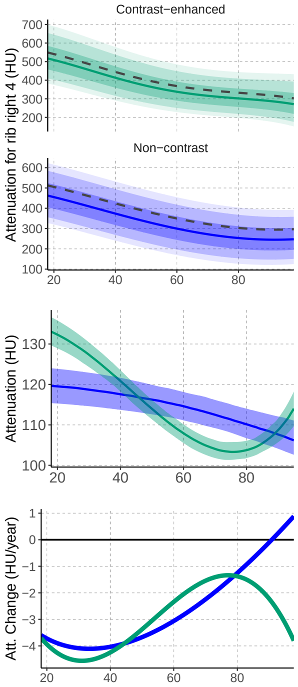

rib right 5

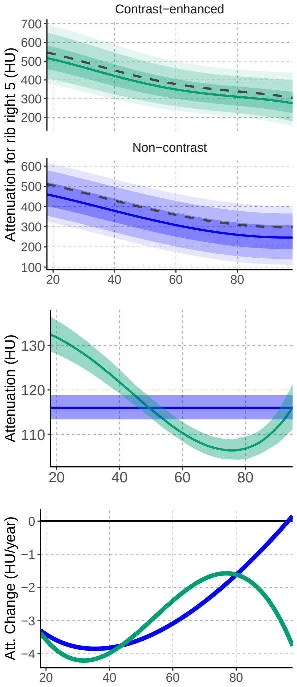

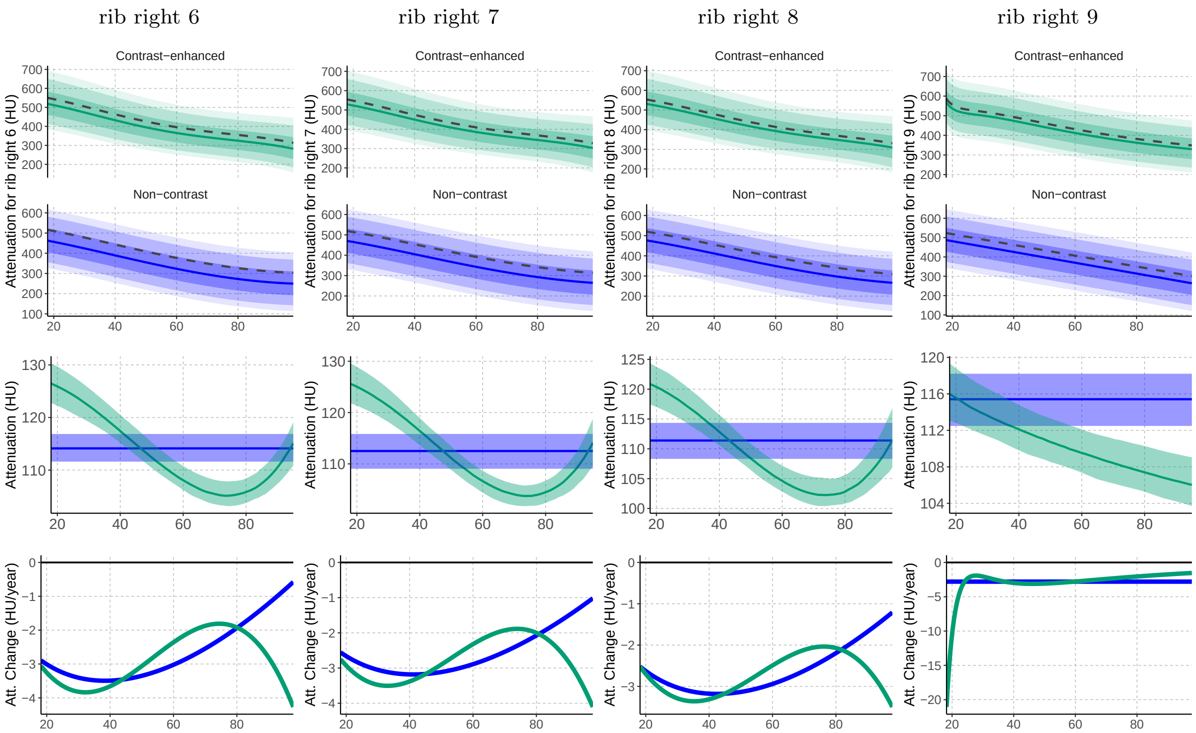
rib right 6
rib right 7
rib right 8
rib right 9
Contrast−enhanced
Non−contrast
Attenuation for rib right 6 (HU)
Attenuation for rib right 7 (HU)
Attenuation for rib right 8 (HU)
Attenuation for rib right 9 (HU)
Attenuation (HU)
Att. Change (HU/year)

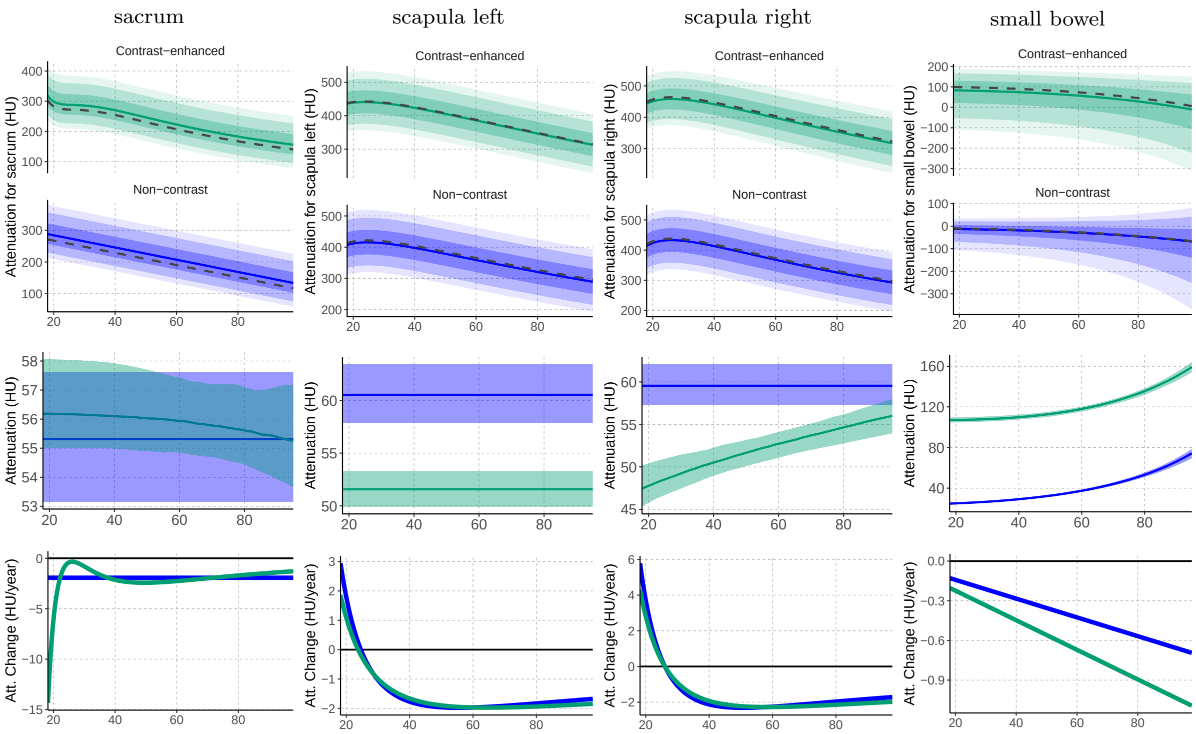
sacrum
scapula left
scapula right
small bowel
Contrast−enhanced
Non−contrast
Attenuation for sacrum (HU)
Attenuation for scapula left (HU)
Attenuation for scapula right (HU)
Attenuation for small bowel (HU)
Attenuation (HU)
Att. Change (HU/year)

spleen

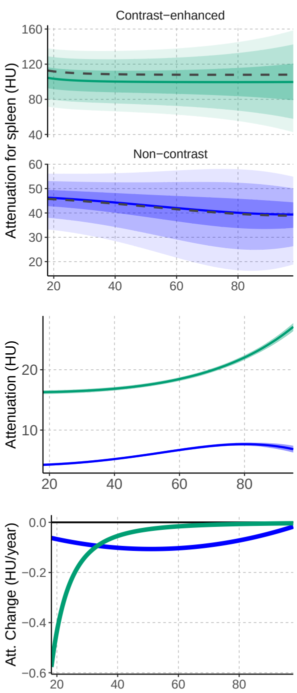

sternum

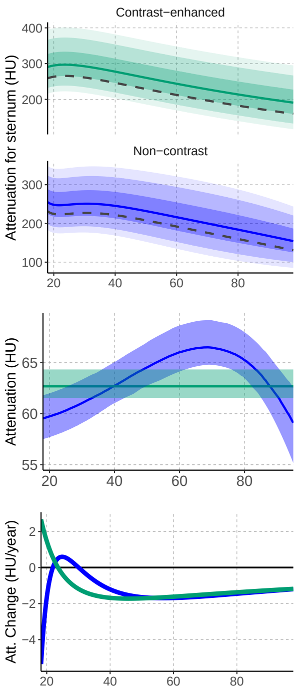

stomach

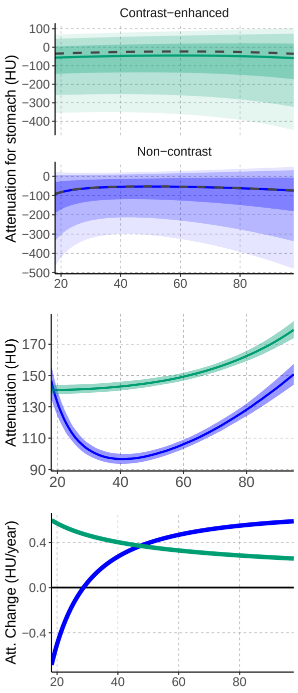

subclavian artery left

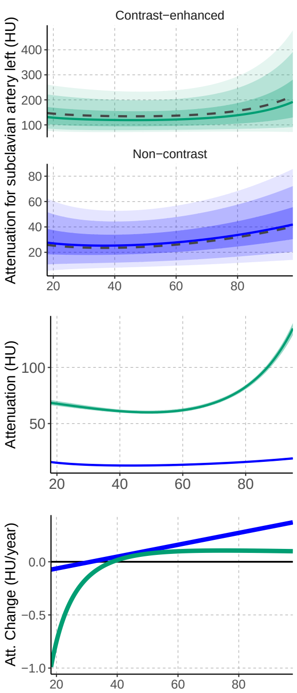

subclavian artery right

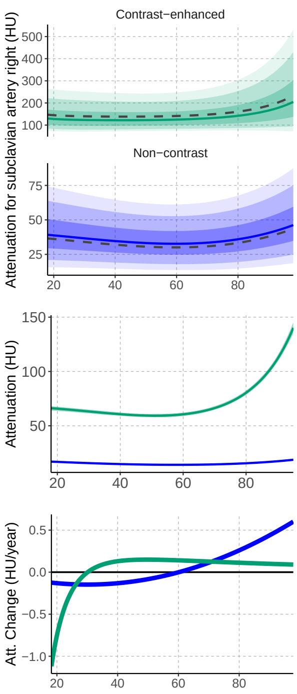

superior vena cava

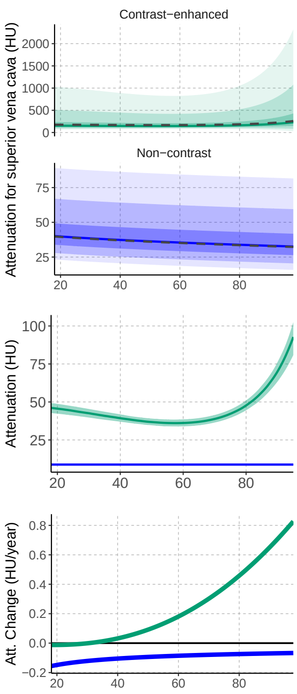

thyroid gland

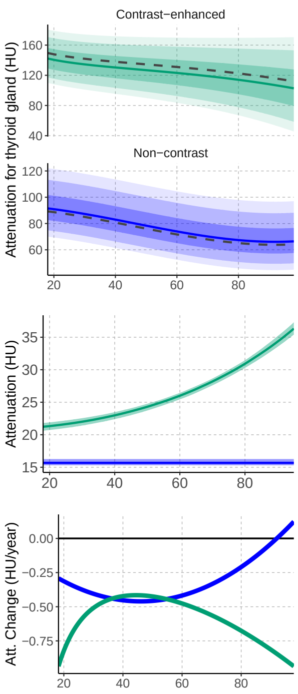

trachea

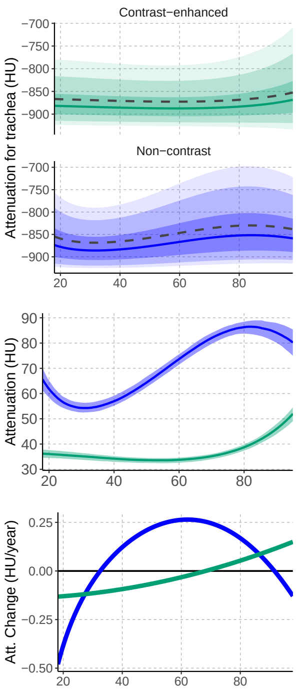

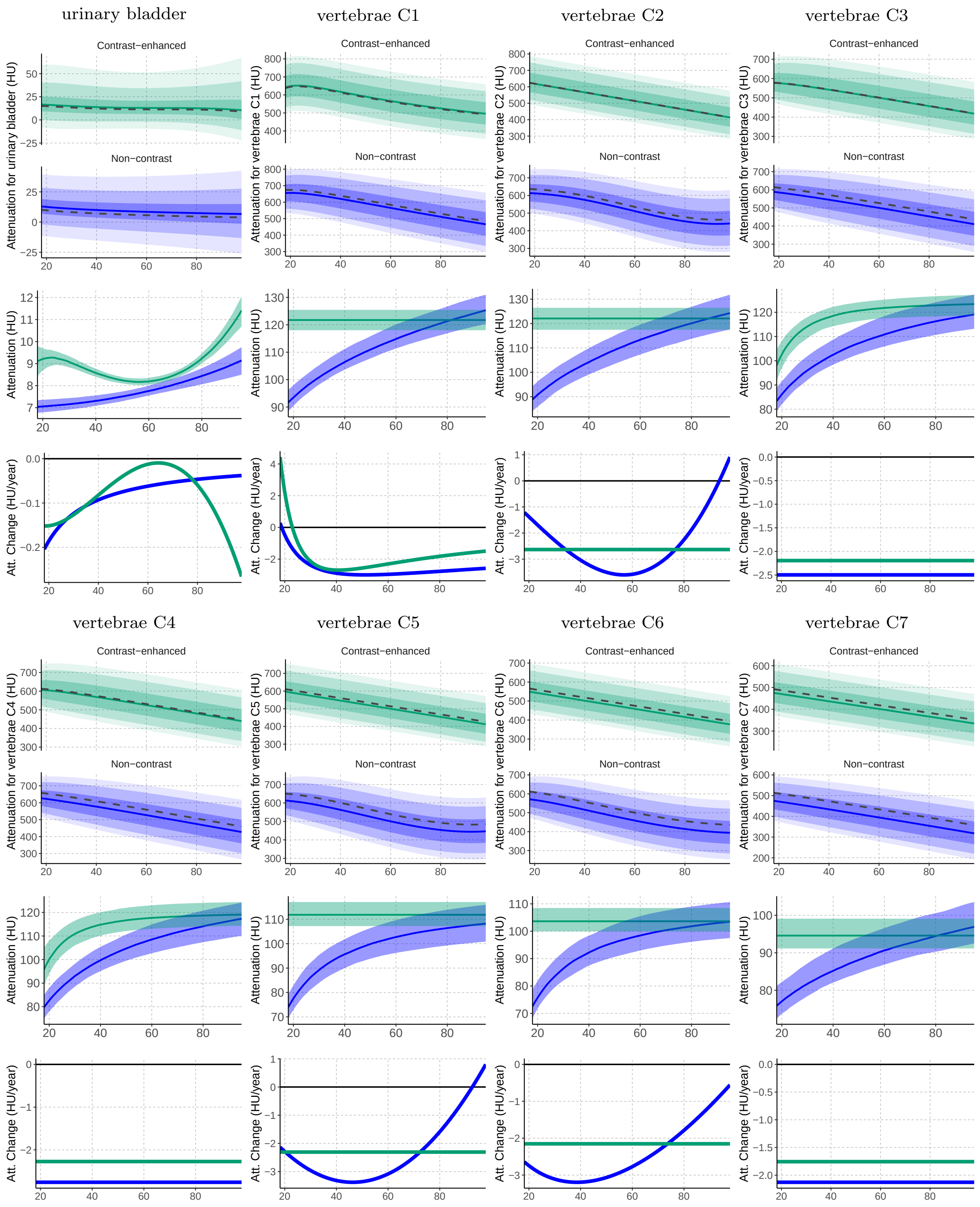
urinary bladder
vertebrae C1
vertebrae C2
vertebrae C3
Contrast−enhanced
Non−contrast
Attenuation for urinary bladder (HU)
Attenuation for vertebrae C1 (HU)
Attenuation for vertebrae C2 (HU)
Attenuation for vertebrae C3 (HU)
Attenuation (HU)
Att. Change (HU/year)
vertebrae C4
vertebrae C5
vertebrae C6
vertebrae C7
Contrast−enhanced
Non−contrast
Attenuation for vertebrae C4 (HU)
Attenuation for vertebrae C5 (HU)
Attenuation for vertebrae C6 (HU)
Attenuation for vertebrae C7 (HU)
Attenuation (HU)
Att. Change (HU/year)

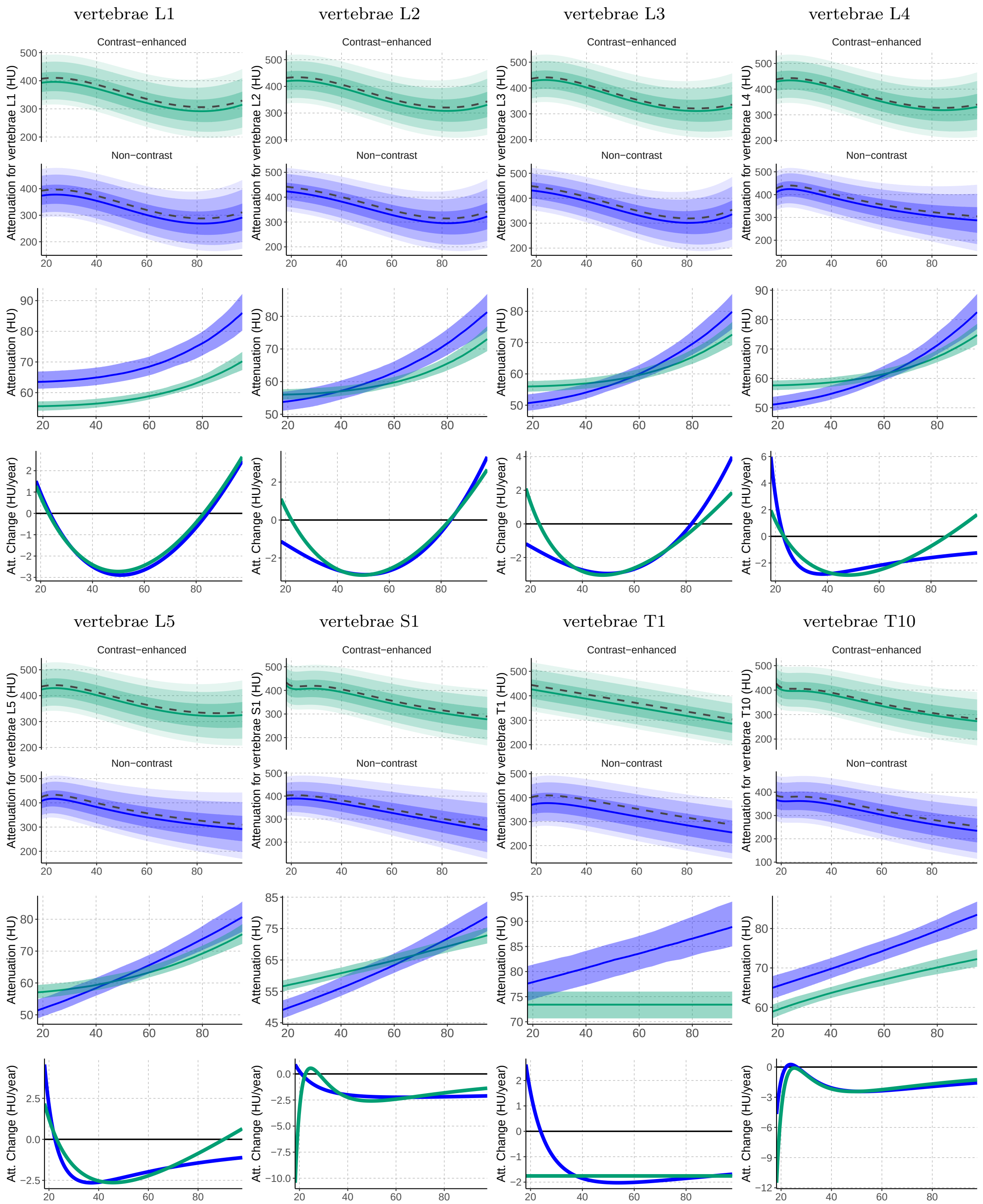
vertebrae L1
vertebrae L2
vertebrae L3
vertebrae L4
Contrast−enhanced
Non−contrast
Attenuation for vertebrae L1 (HU)
Attenuation for vertebrae L2 (HU)
Attenuation for vertebrae L3 (HU)
Attenuation for vertebrae L4 (HU)
Attenuation (HU)
Att. Change (HU/year)
vertebrae L5
vertebrae S1
vertebrae T1
vertebrae T10
Attenuation for vertebrae L5 (HU)
Attenuation for vertebrae S1 (HU)
Attenuation for vertebrae T1 (HU)
Attenuation for vertebrae T10 (HU)

vertebrae T11

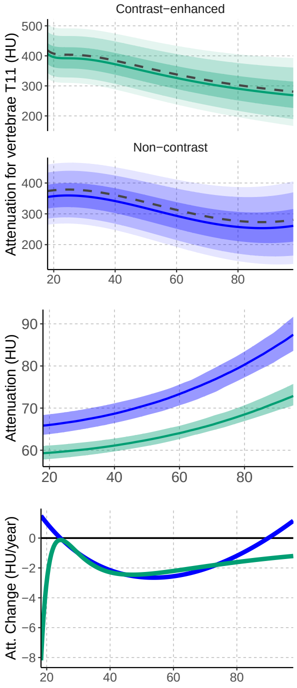

vertebrae T12

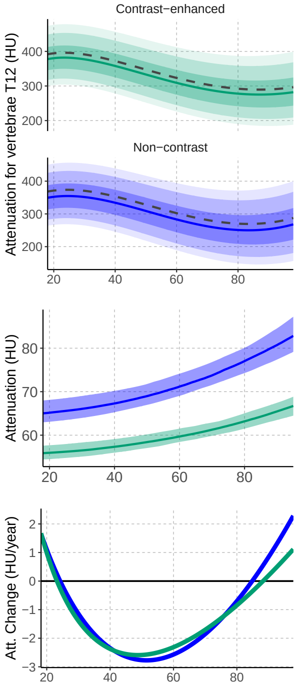

vertebrae T2

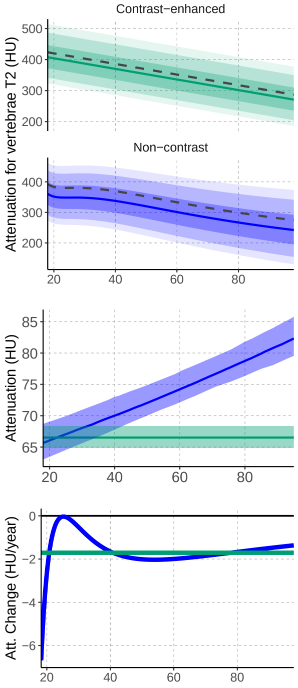

vertebrae T3

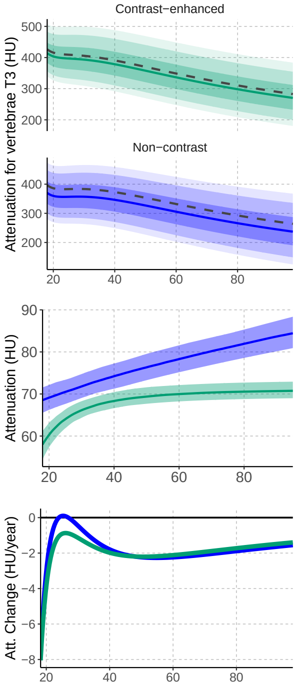

vertebrae T4

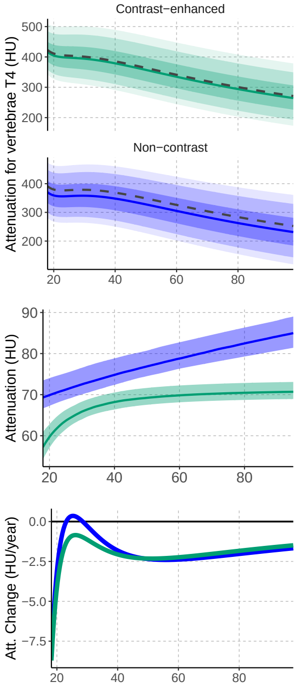

vertebrae T5

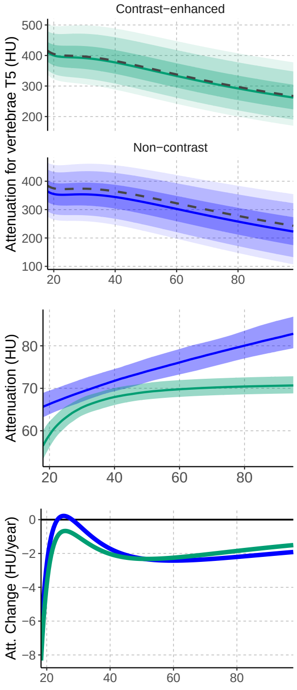

vertebrae T6

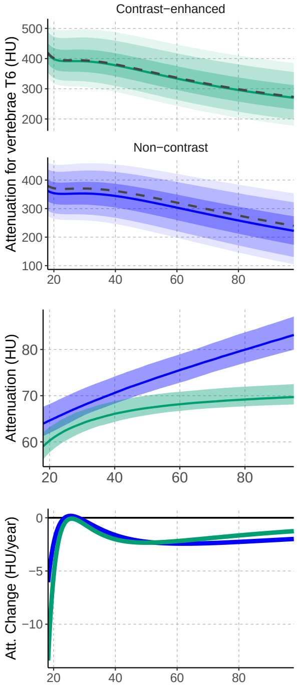

vertebrae T7

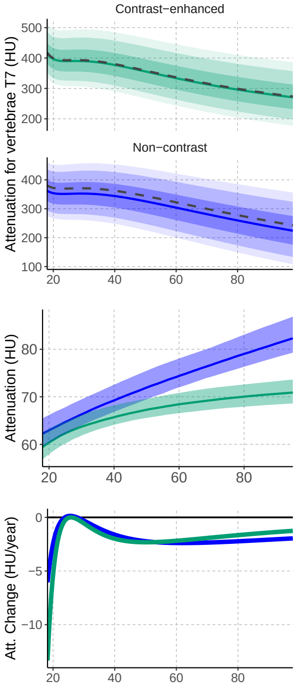

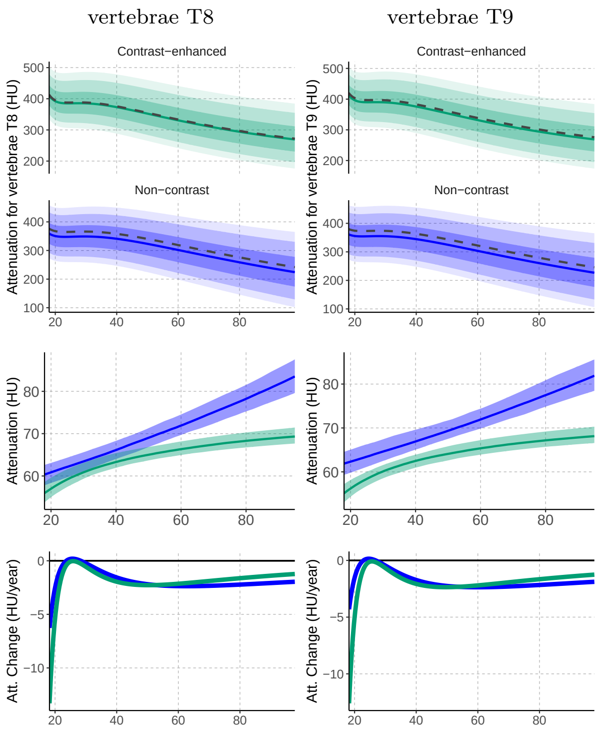
vertebrae T8
vertebrae T9
Contrast−enhanced
Non−contrast
Attenuation for vertebrae T8 (HU)
Attenuation for vertebrae T9 (HU)
Attenuation (HU)
Att. Change (HU/year)
20
40
60
80

Longitudinal reference charts for CT volume

Supplementary Figure 9: Extension of Figure 7 from the main paper with plots for all anatomical structures.

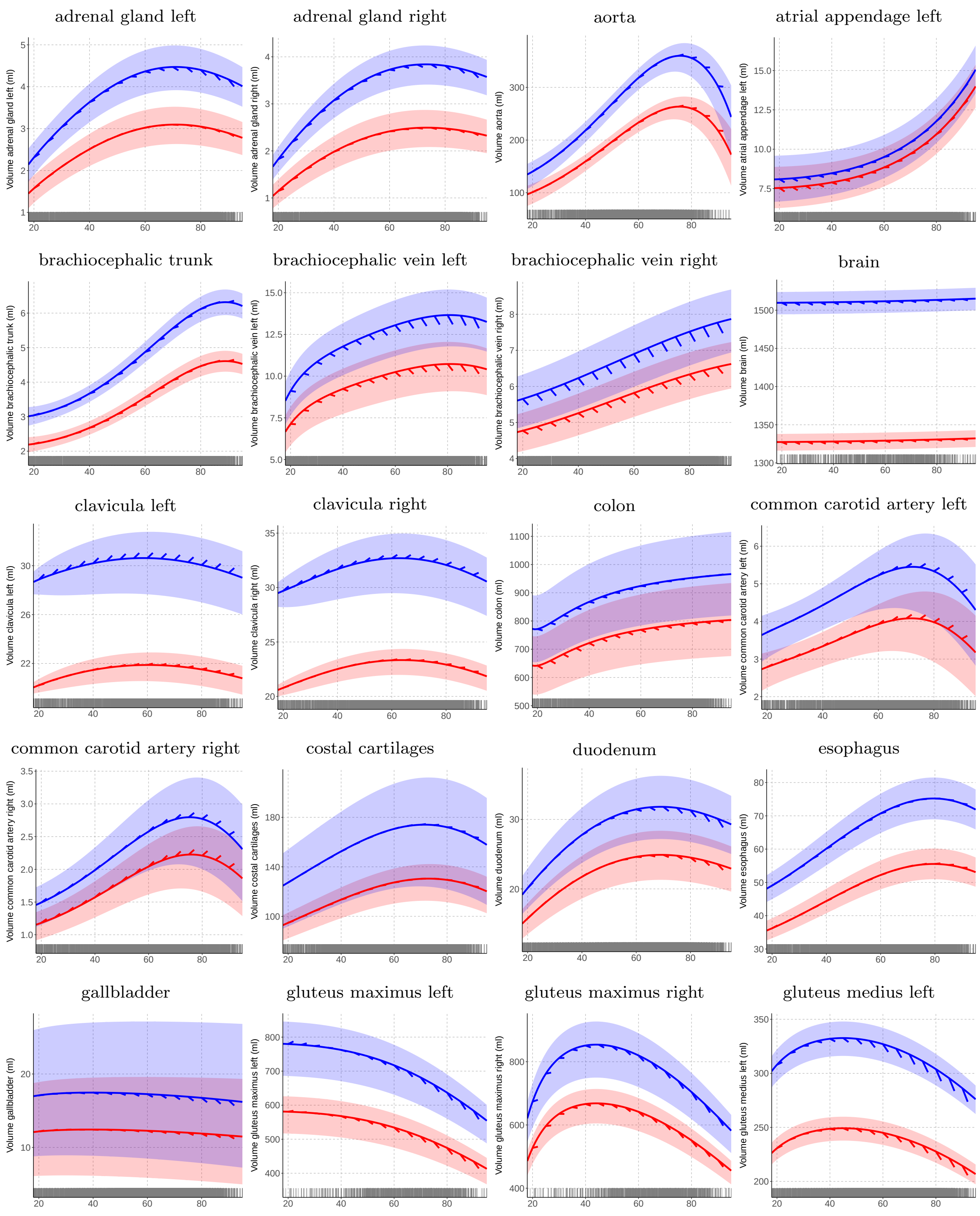

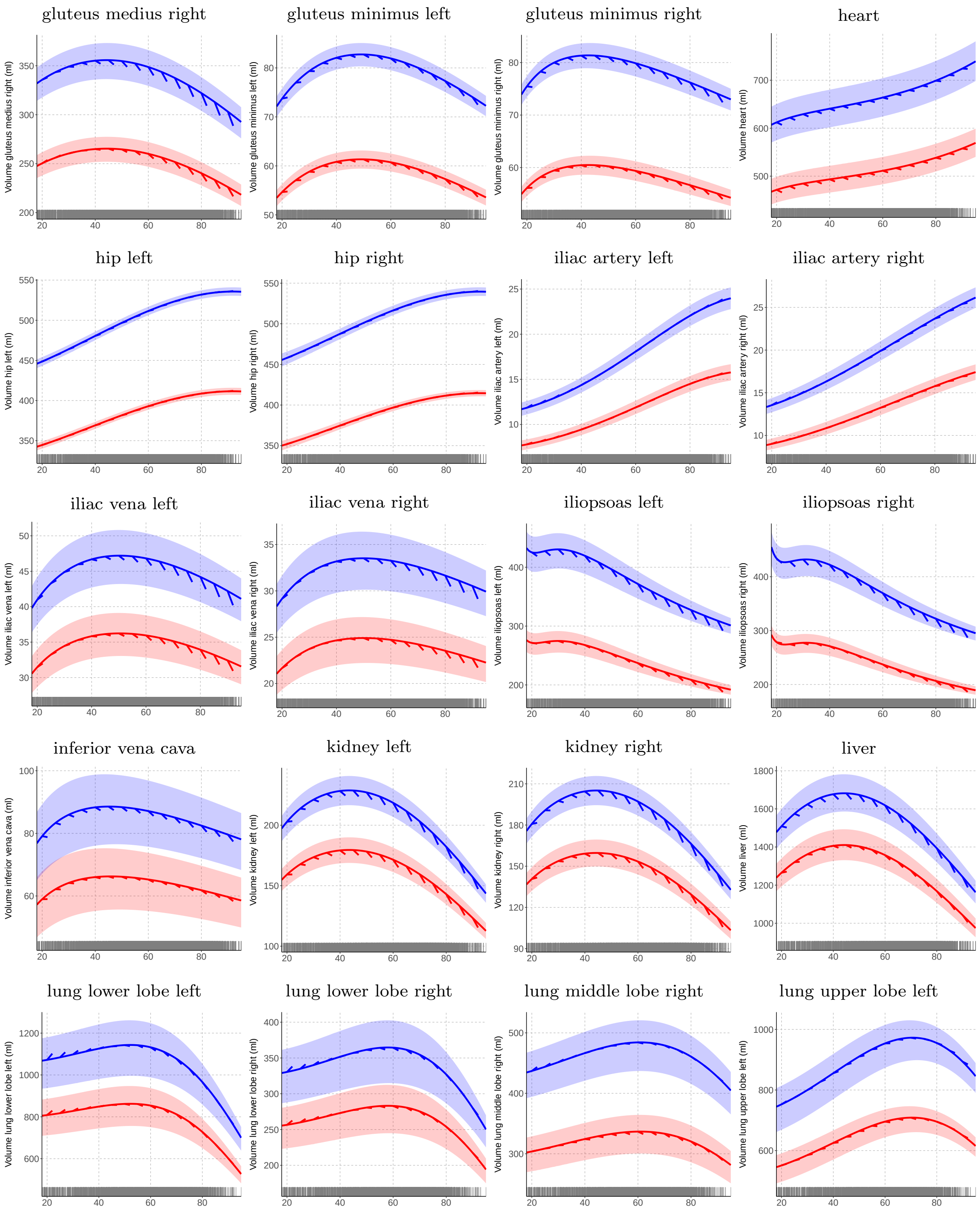
gluteus medius right
Volume gluteus medius right (ml)
gluteus minimus left
Volume gluteus minimus left (ml)
gluteus minimus right
Volume gluteus minimus right (ml)
heart
Volume heart (ml)
hip left
Volume hip left (ml)
hip right
Volume hip right (ml)
iliac artery left
Volume iliac artery left (ml)
iliac artery right
Volume iliac artery right (ml)
iliac vena left
Volume iliac vena left (ml)
iliac vena right
Volume iliac vena right (ml)
iliopsoas left
Volume iliopsoas left (ml)
iliopsoas right
Volume iliopsoas right (ml)
inferior vena cava
Volume inferior vena cava (ml)
kidney left
Volume kidney left (ml)
kidney right
Volume kidney right (ml)
liver
Volume liver (ml)
lung lower lobe left
Volume lung lower lobe left (ml)
lung lower lobe right
Volume lung lower lobe right (ml)
lung middle lobe right
Volume lung middle lobe right (ml)
lung upper lobe left
Volume lung upper lobe left (ml)

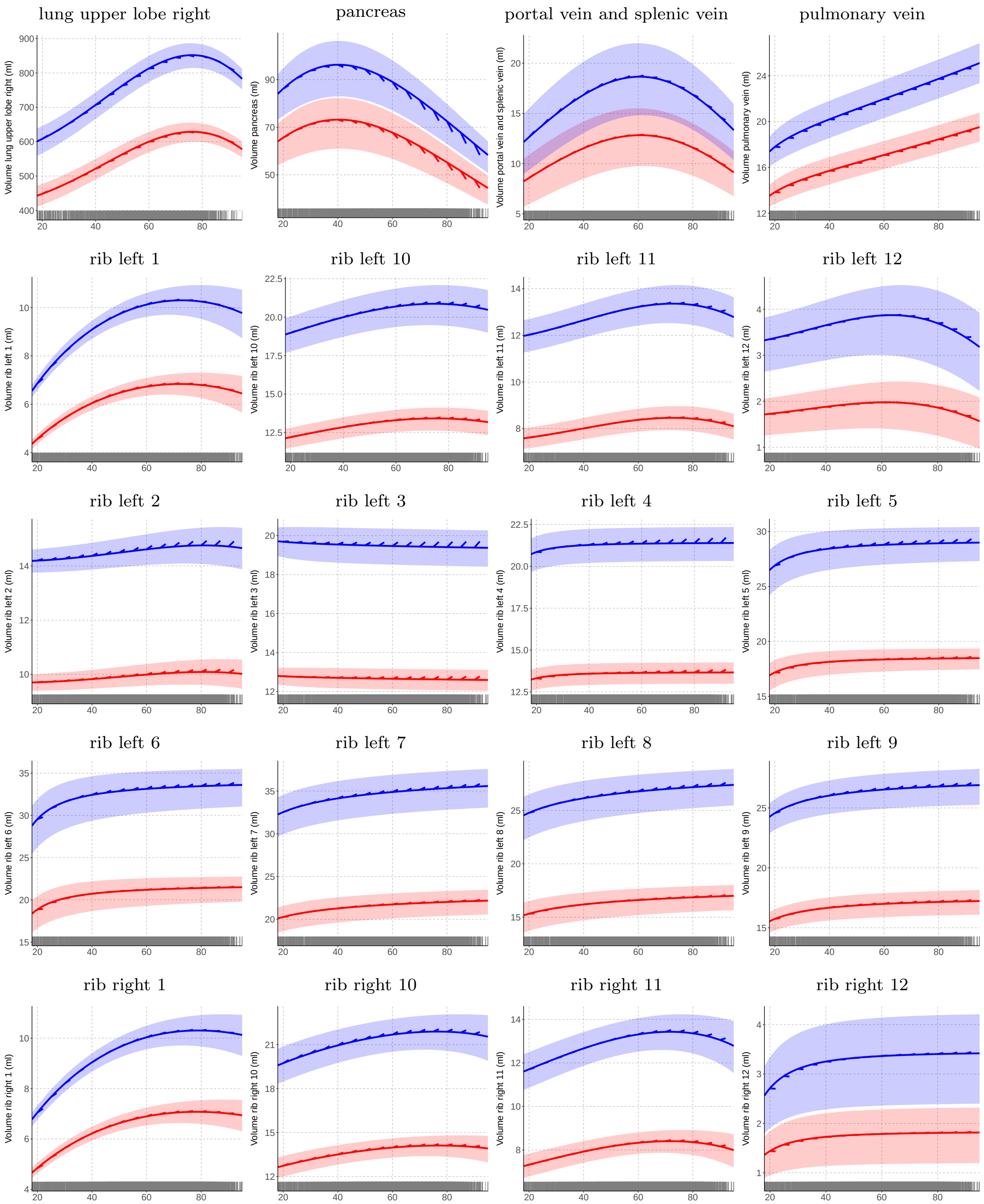
lung upper lobe right
Volume lung upper lobe right (ml)
pancreas
Volume pancreas (ml)
portal vein and splenic vein
Volume portal vein and splenic vein (ml)
pulmonary vein
Volume pulmonary vein (ml)
rib left 1
Volume rib left 1 (ml)
rib left 10
Volume rib left 10 (ml)
rib left 11
Volume rib left 11 (ml)
rib left 12
Volume rib left 12 (ml)
rib left 2
Volume rib left 2 (ml)
rib left 3
Volume rib left 3 (ml)
rib left 4
Volume rib left 4 (ml)
rib left 5
Volume rib left 5 (ml)
rib left 6
Volume rib left 6 (ml)
rib left 7
Volume rib left 7 (ml)
rib left 8
Volume rib left 8 (ml)
rib left 9
Volume rib left 9 (ml)
rib right 1
Volume rib right 1 (ml)
rib right 10
Volume rib right 10 (ml)
rib right 11
Volume rib right 11 (ml)
rib right 12
Volume rib right 12 (ml)

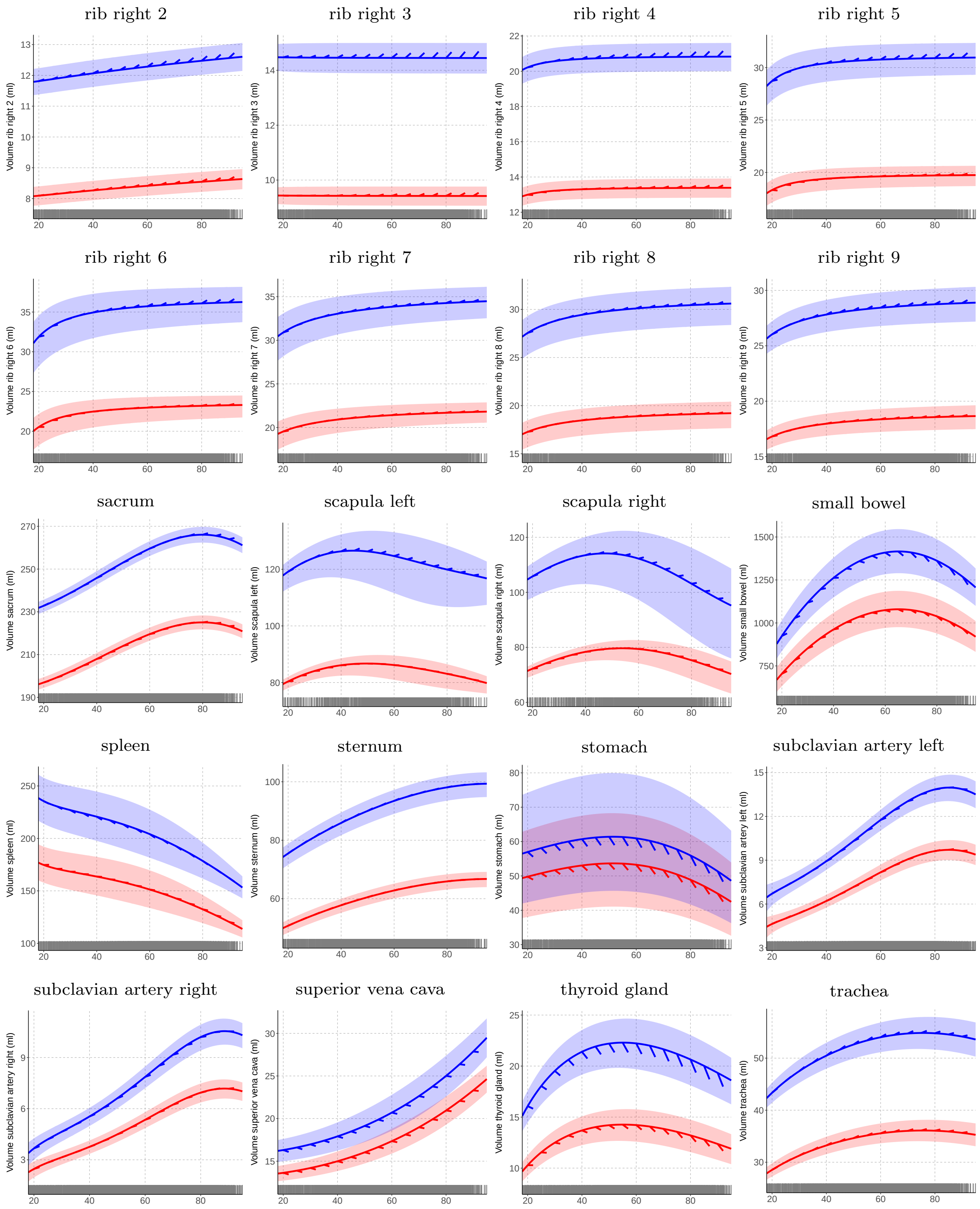
rib right 2
Volume rib right 2 (ml)
rib right 3
Volume rib right 3 (ml)
rib right 4
Volume rib right 4 (ml)
rib right 5
Volume rib right 5 (ml)
rib right 6
Volume rib right 6 (ml)
rib right 7
Volume rib right 7 (ml)
rib right 8
Volume rib right 8 (ml)
rib right 9
Volume rib right 9 (ml)
sacrum
Volume sacrum (ml)
scapula left
Volume scapula left (ml)
scapula right
Volume scapula right (ml)
small bowel
Volume small bowel (ml)
spleen
Volume spleen (ml)
sternum
Volume sternum (ml)
stomach
Volume stomach (ml)
subclavian artery left
Volume subclavian artery left (ml)
subclavian artery right
Volume subclavian artery right (ml)
superior vena cava
Volume superior vena cava (ml)
thyroid gland
Volume thyroid gland (ml)
trachea
Volume trachea (ml)

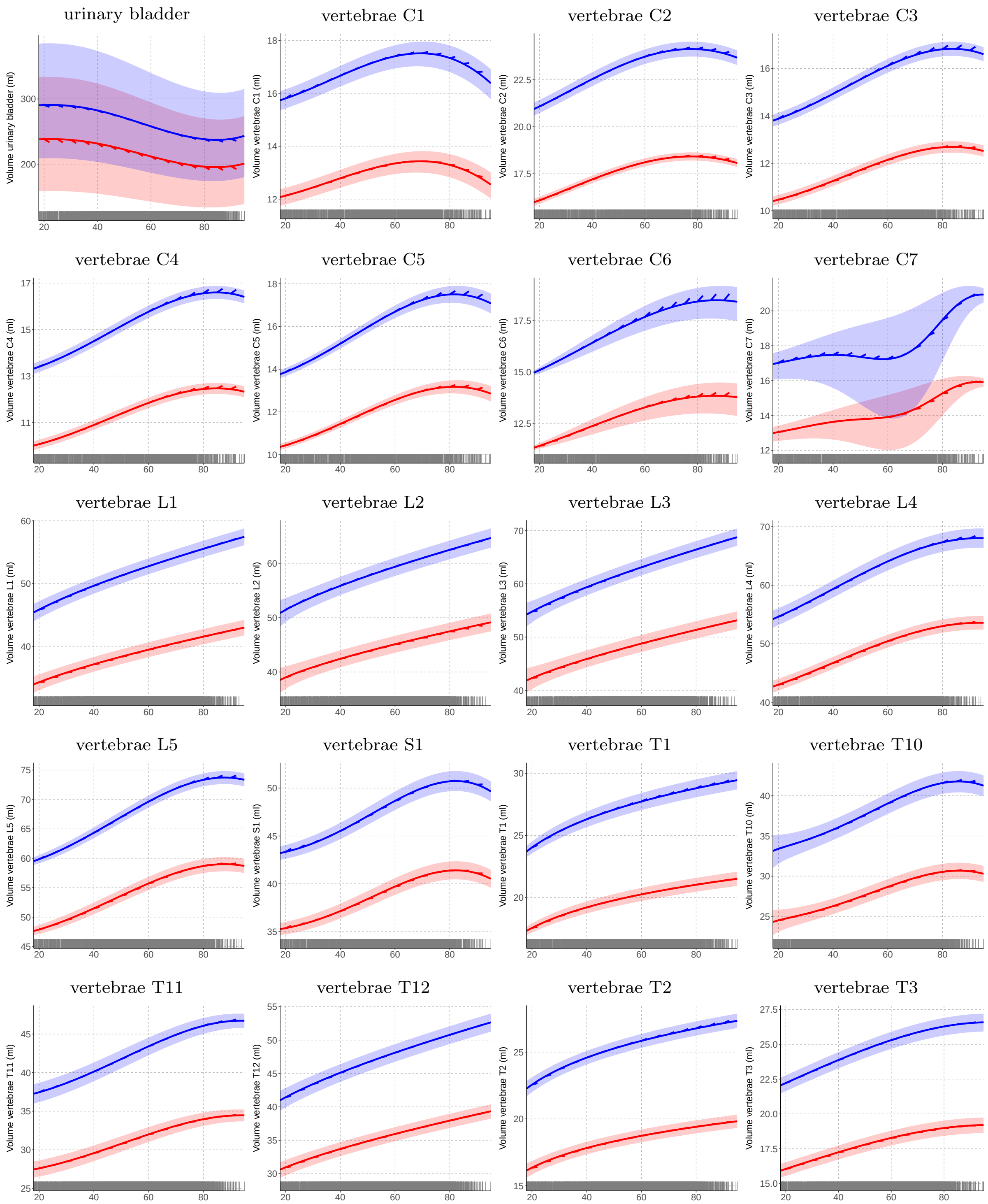
urinary bladder
Volume urinary bladder (ml)
vertebrae C1
Volume vertebrae C1 (ml)
vertebrae C2
Volume vertebrae C2 (ml)
vertebrae C3
Volume vertebrae C3 (ml)
vertebrae C4
Volume vertebrae C4 (ml)
vertebrae C5
Volume vertebrae C5 (ml)
vertebrae C6
Volume vertebrae C6 (ml)
vertebrae C7
Volume vertebrae C7 (ml)
vertebrae L1
Volume vertebrae L1 (ml)
vertebrae L2
Volume vertebrae L2 (ml)
vertebrae L3
Volume vertebrae L3 (ml)
vertebrae L4
Volume vertebrae L4 (ml)
vertebrae L5
Volume vertebrae L5 (ml)
vertebrae S1
Volume vertebrae S1 (ml)
vertebrae T1
Volume vertebrae T1 (ml)
vertebrae T10
Volume vertebrae T10 (ml)
vertebrae T11
Volume vertebrae T11 (ml)
vertebrae T12
Volume vertebrae T12 (ml)
vertebrae T2
Volume vertebrae T2 (ml)
vertebrae T3
Volume vertebrae T3 (ml)

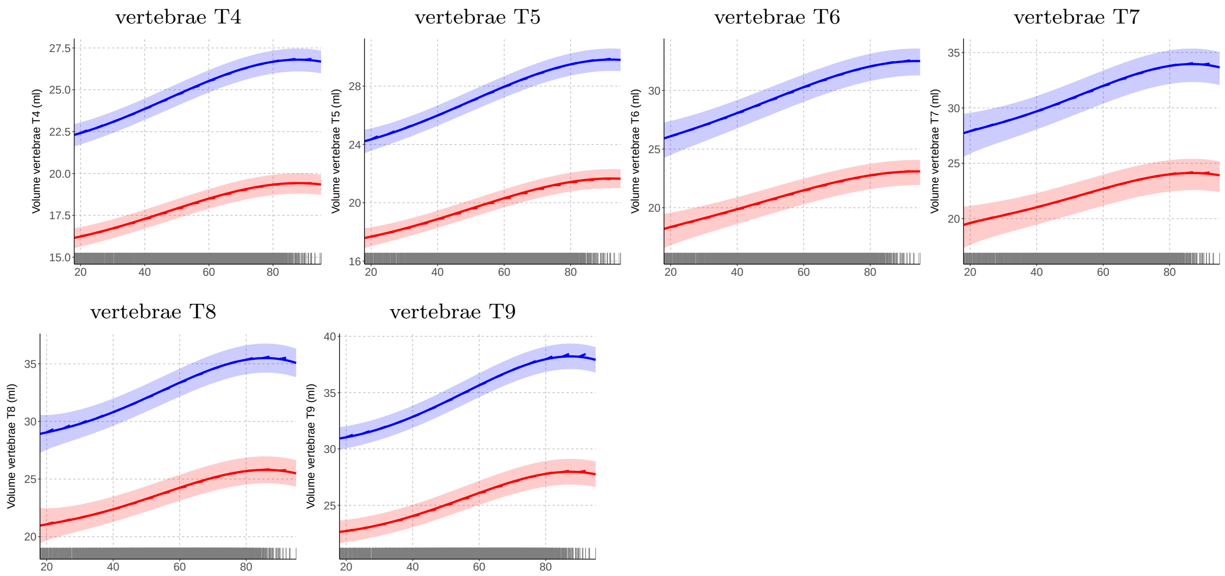
vertebrae T4
Volume vertebrae T4 (ml)
vertebrae T5
Volume vertebrae T5 (ml)
vertebrae T6
Volume vertebrae T6 (ml)
vertebrae T7
Volume vertebrae T7 (ml)
vertebrae T8
Volume vertebrae T8 (ml)
vertebrae T9
Volume vertebrae T9 (ml)

Longitudinal reference charts for CT attenuation

Supplementary Figure 10: Extension of Figure 8 from the main paper with plots for all anatomical structures.

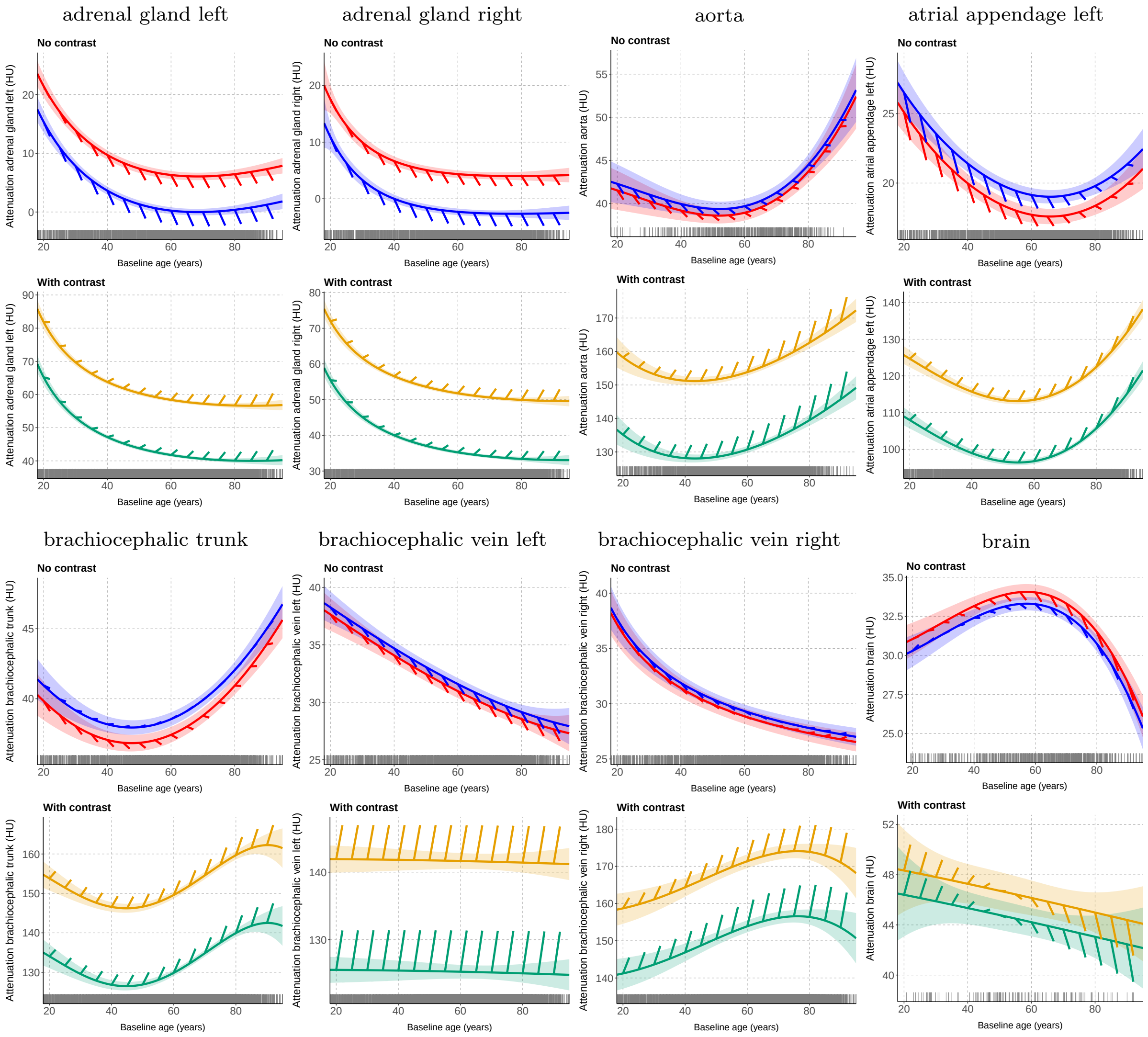

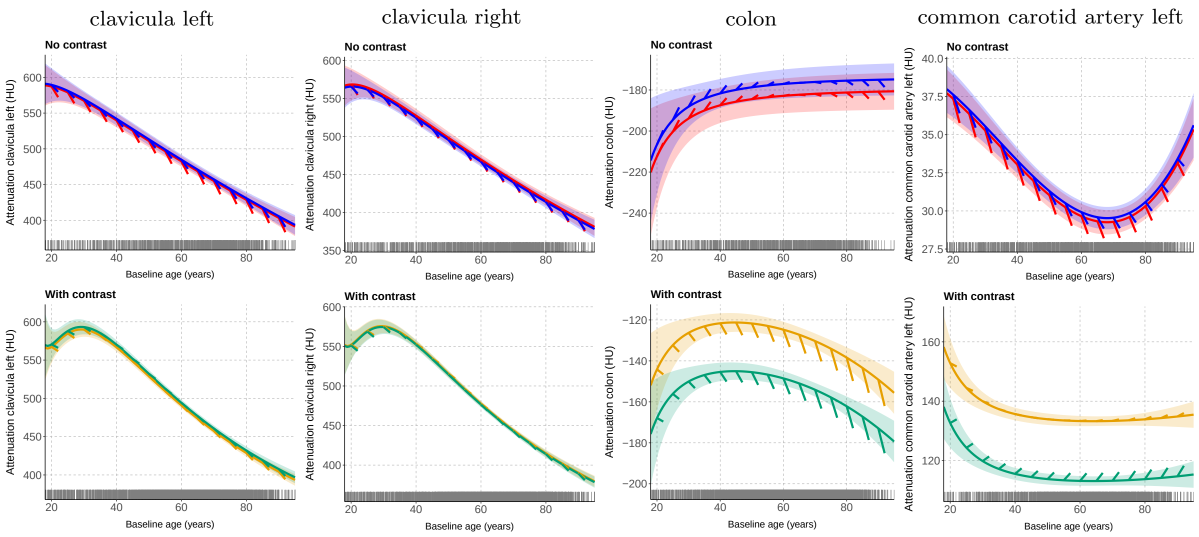
clavicula left
clavicula right
colon
common carotid artery left
No contrast
With contrast
Attenuation clavicula left (HU)
Attenuation clavicula right (HU)
Attenuation colon (HU)
Attenuation common carotid artery left (HU)
Baseline age (years)

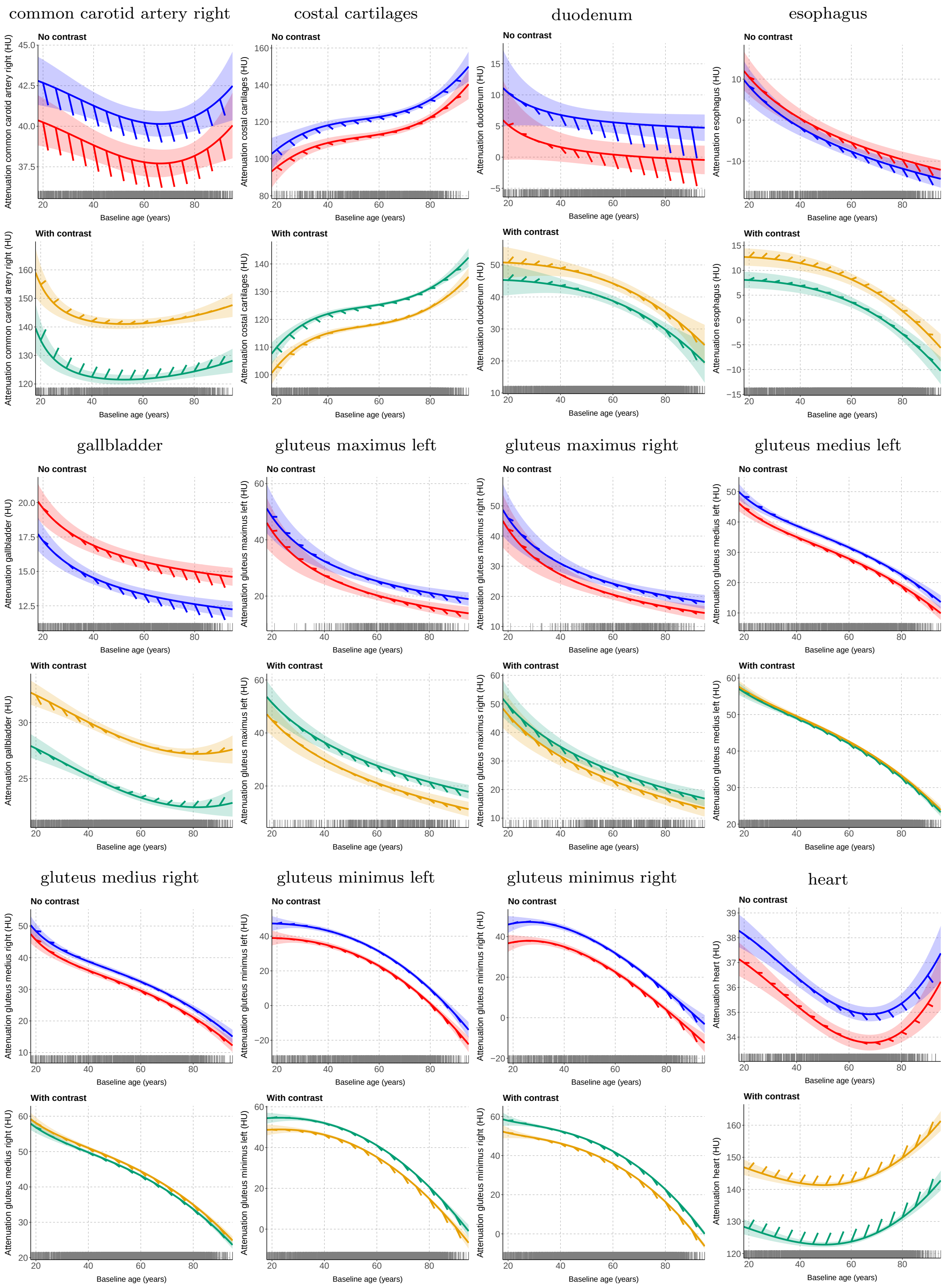
common carotid artery right
No contrast
Attenuation common carotid artery right (HU)
Baseline age (years)
With contrast
Attenuation common carotid artery right (HU)
Baseline age (years)
costal cartilages
No contrast
Attenuation costal cartilages (HU)
Baseline age (years)
With contrast
Attenuation costal cartilages (HU)
Baseline age (years)
duodenum
No contrast
Attenuation duodenum (HU)
Baseline age (years)
With contrast
Attenuation duodenum (HU)
Baseline age (years)
esophagus
No contrast
Attenuation esophagus (HU)
Baseline age (years)
With contrast
Attenuation esophagus (HU)
Baseline age (years)
gallbladder
No contrast
Attenuation gallbladder (HU)
Baseline age (years)
With contrast
Attenuation gallbladder (HU)
Baseline age (years)
gluteus maximus left
No contrast
Attenuation gluteus maximus left (HU)
Baseline age (years)
With contrast
Attenuation gluteus maximus left (HU)
Baseline age (years)
gluteus maximus right
No contrast
Attenuation gluteus maximus right (HU)
Baseline age (years)
With contrast
Attenuation gluteus maximus right (HU)
Baseline age (years)
gluteus medius left
No contrast
Attenuation gluteus medius left (HU)
Baseline age (years)
With contrast
Attenuation gluteus medius left (HU)
Baseline age (years)
gluteus medius right
No contrast
Attenuation gluteus medius right (HU)
Baseline age (years)
With contrast
Attenuation gluteus medius right (HU)
Baseline age (years)
gluteus minimus left
No contrast
Attenuation gluteus minimus left (HU)
Baseline age (years)
With contrast
Attenuation gluteus minimus left (HU)
Baseline age (years)
gluteus minimus right
No contrast
Attenuation gluteus minimus right (HU)
Baseline age (years)
With contrast
Attenuation gluteus minimus right (HU)
Baseline age (years)
heart
No contrast
Attenuation heart (HU)
Baseline age (years)
With contrast
Attenuation heart (HU)
Baseline age (years)

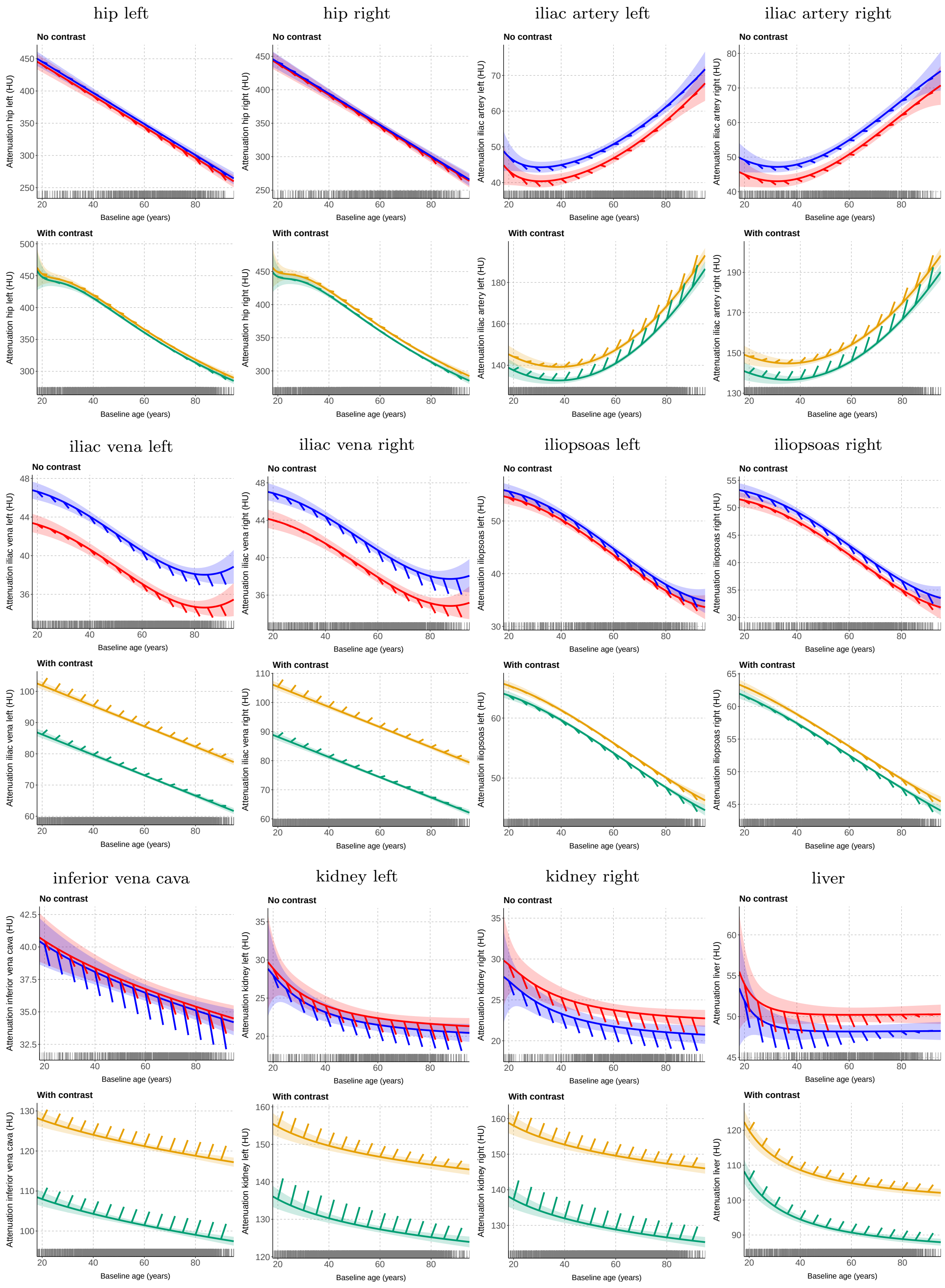
hip left
No contrast
Attenuation hip left (HU)
Baseline age (years)
With contrast
hip right
Attenuation hip right (HU)
iliac artery left
Attenuation iliac artery left (HU)
iliac artery right
Attenuation iliac artery right (HU)
iliac vena left
Attenuation iliac vena left (HU)
iliac vena right
Attenuation iliac vena right (HU)
iliopsoas left
Attenuation iliopsoas left (HU)
iliopsoas right
Attenuation iliopsoas right (HU)
inferior vena cava
Attenuation inferior vena cava (HU)
kidney left
Attenuation kidney left (HU)
kidney right
Attenuation kidney right (HU)
liver
Attenuation liver (HU)

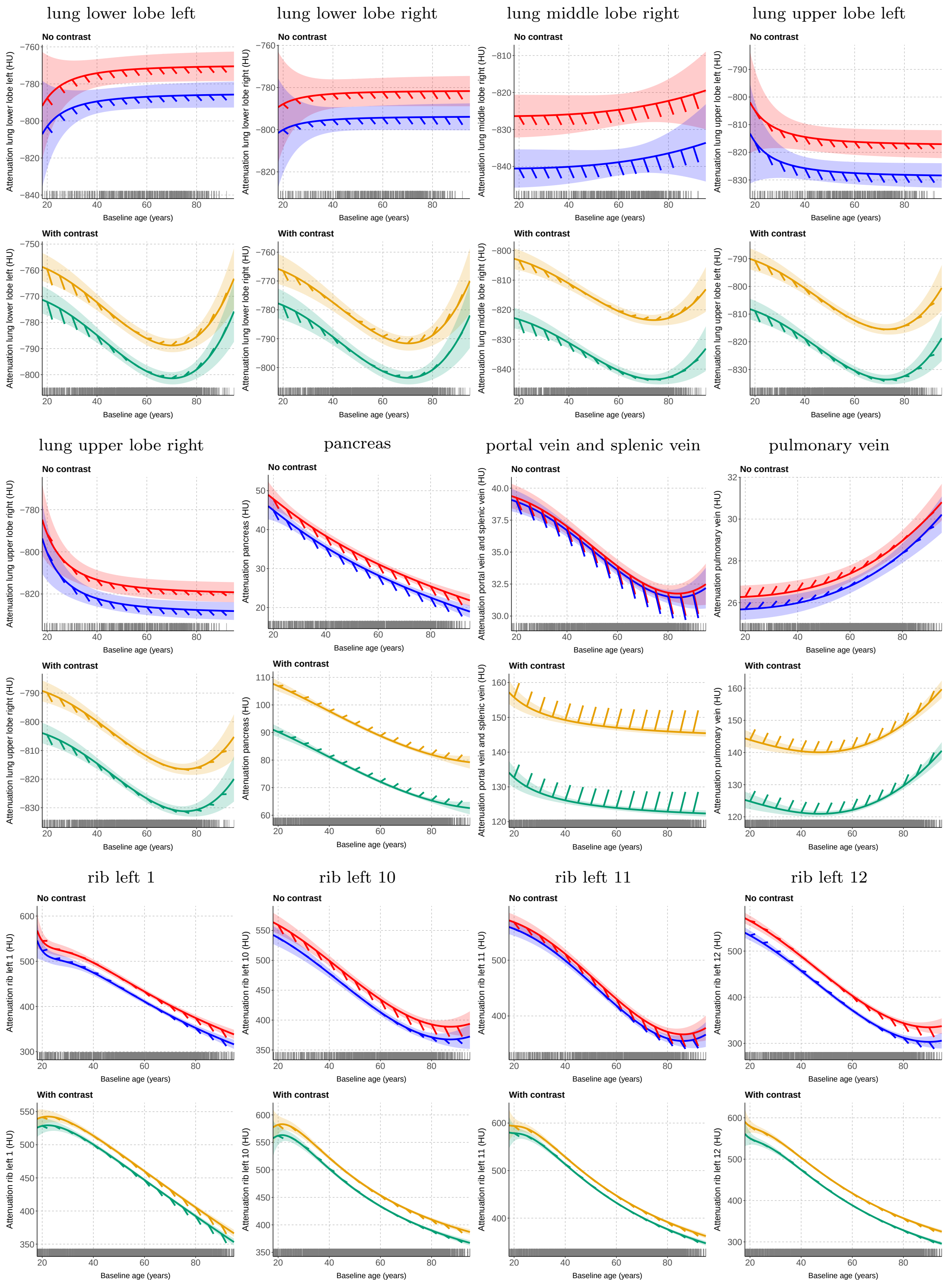
lung lower lobe left
No contrast
Attenuation lung lower lobe left (HU)
Baseline age (years)
With contrast
Attenuation lung lower lobe left (HU)
Baseline age (years)
lung lower lobe right
No contrast
Attenuation lung lower lobe right (HU)
Baseline age (years)
With contrast
Attenuation lung lower lobe right (HU)
Baseline age (years)
lung middle lobe right
No contrast
Attenuation lung middle lobe right (HU)
Baseline age (years)
With contrast
Attenuation lung middle lobe right (HU)
Baseline age (years)
lung upper lobe left
No contrast
Attenuation lung upper lobe left (HU)
Baseline age (years)
With contrast
Attenuation lung upper lobe left (HU)
Baseline age (years)
lung upper lobe right
No contrast
Attenuation lung upper lobe right (HU)
Baseline age (years)
With contrast
Attenuation lung upper lobe right (HU)
Baseline age (years)
pancreas
No contrast
Attenuation pancreas (HU)
Baseline age (years)
With contrast
Attenuation pancreas (HU)
Baseline age (years)
portal vein and splenic vein
No contrast
Attenuation portal vein and splenic vein (HU)
Baseline age (years)
With contrast
Attenuation portal vein and splenic vein (HU)
Baseline age (years)
pulmonary vein
No contrast
Attenuation pulmonary vein (HU)
Baseline age (years)
With contrast
Attenuation pulmonary vein (HU)
Baseline age (years)
rib left 1
No contrast
Attenuation rib left 1 (HU)
Baseline age (years)
With contrast
Attenuation rib left 1 (HU)
Baseline age (years)
rib left 10
No contrast
Attenuation rib left 10 (HU)
Baseline age (years)
With contrast
Attenuation rib left 10 (HU)
Baseline age (years)
rib left 11
No contrast
Attenuation rib left 11 (HU)
Baseline age (years)
With contrast
Attenuation rib left 11 (HU)
Baseline age (years)
rib left 12
No contrast
Attenuation rib left 12 (HU)
Baseline age (years)
With contrast
Attenuation rib left 12 (HU)
Baseline age (years)

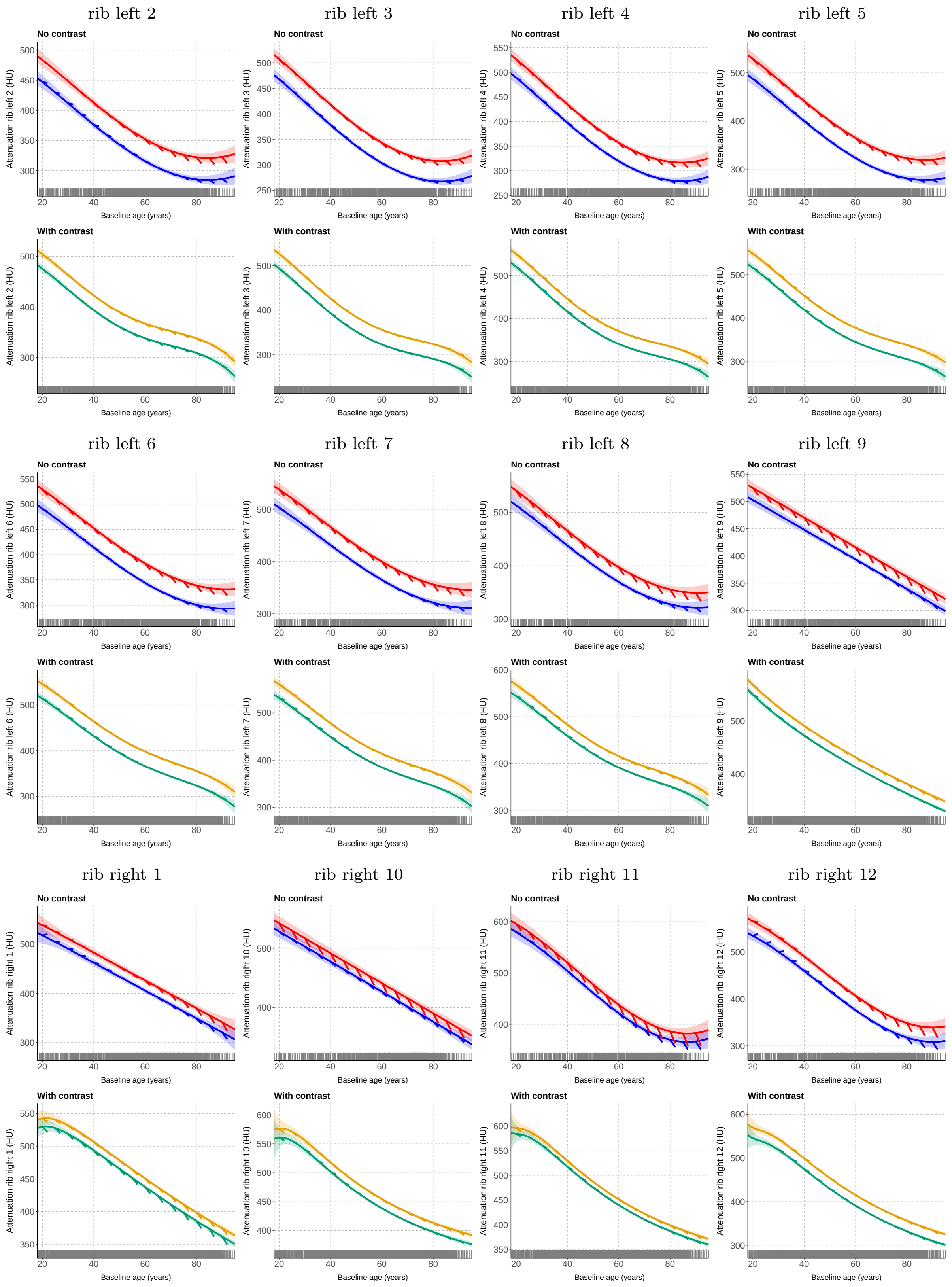
rib left 2
No contrast
Attenuation rib left 2 (HU)
With contrast
rib left 3
No contrast
Attenuation rib left 3 (HU)
With contrast
rib left 4
No contrast
Attenuation rib left 4 (HU)
With contrast
rib left 5
No contrast
Attenuation rib left 5 (HU)
With contrast
rib left 6
No contrast
Attenuation rib left 6 (HU)
With contrast
rib left 7
No contrast
Attenuation rib left 7 (HU)
With contrast
rib left 8
No contrast
Attenuation rib left 8 (HU)
With contrast
rib left 9
No contrast
Attenuation rib left 9 (HU)
With contrast
rib right 1
No contrast
Attenuation rib right 1 (HU)
With contrast
rib right 10
No contrast
Attenuation rib right 10 (HU)
With contrast
rib right 11
No contrast
Attenuation rib right 11 (HU)
With contrast
rib right 12
No contrast
Attenuation rib right 12 (HU)
With contrast
Baseline age (years)

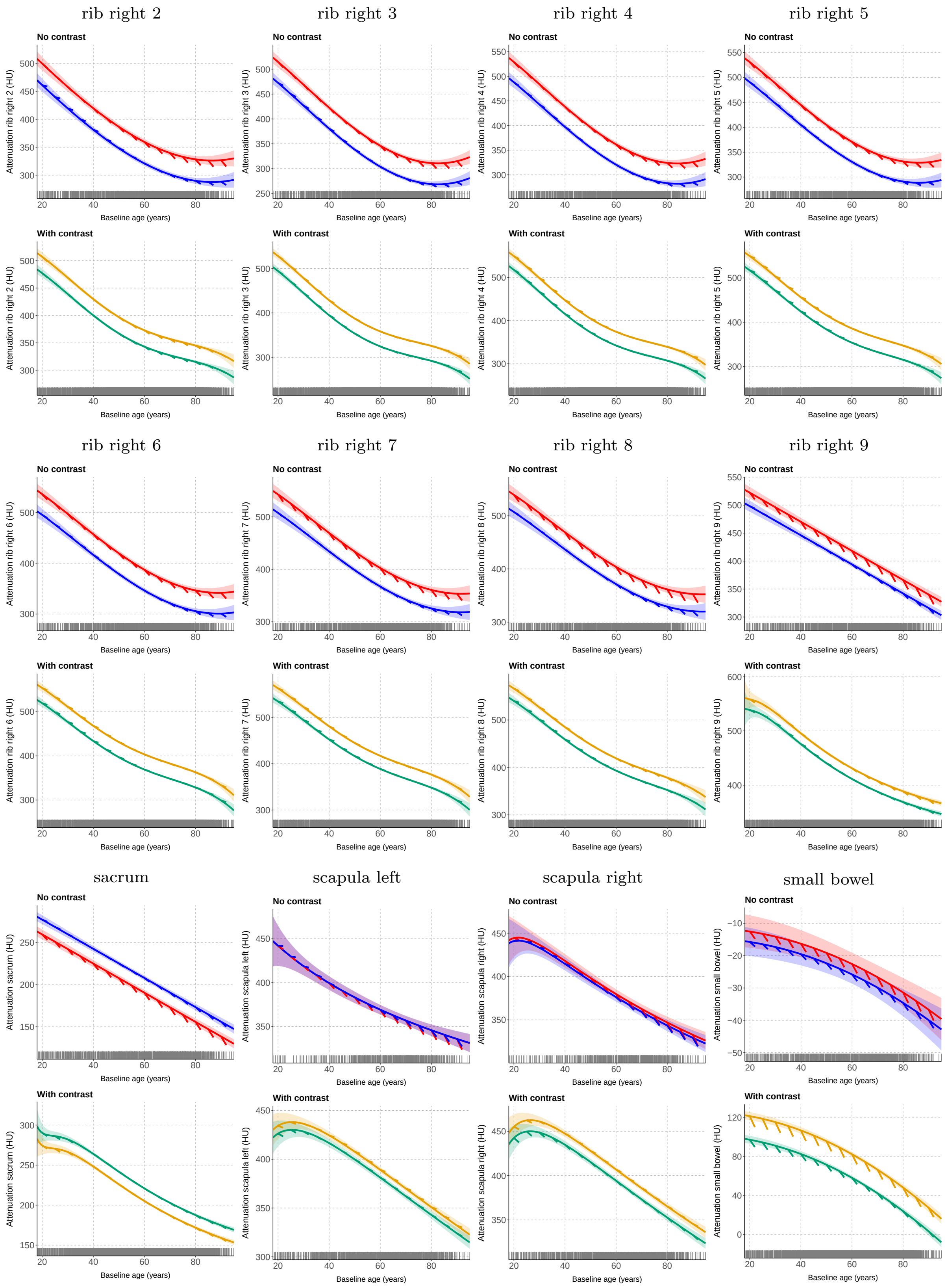
rib right 2
rib right 3
rib right 4
rib right 5
No contrast
With contrast
Attenuation rib right 2 (HU)
Attenuation rib right 3 (HU)
Attenuation rib right 4 (HU)
Attenuation rib right 5 (HU)
Baseline age (years)
rib right 6
rib right 7
rib right 8
rib right 9
Attenuation rib right 6 (HU)
Attenuation rib right 7 (HU)
Attenuation rib right 8 (HU)
Attenuation rib right 9 (HU)
sacrum
scapula left
scapula right
small bowel
Attenuation sacrum (HU)
Attenuation scapula left (HU)
Attenuation scapula right (HU)
Attenuation small bowel (HU)

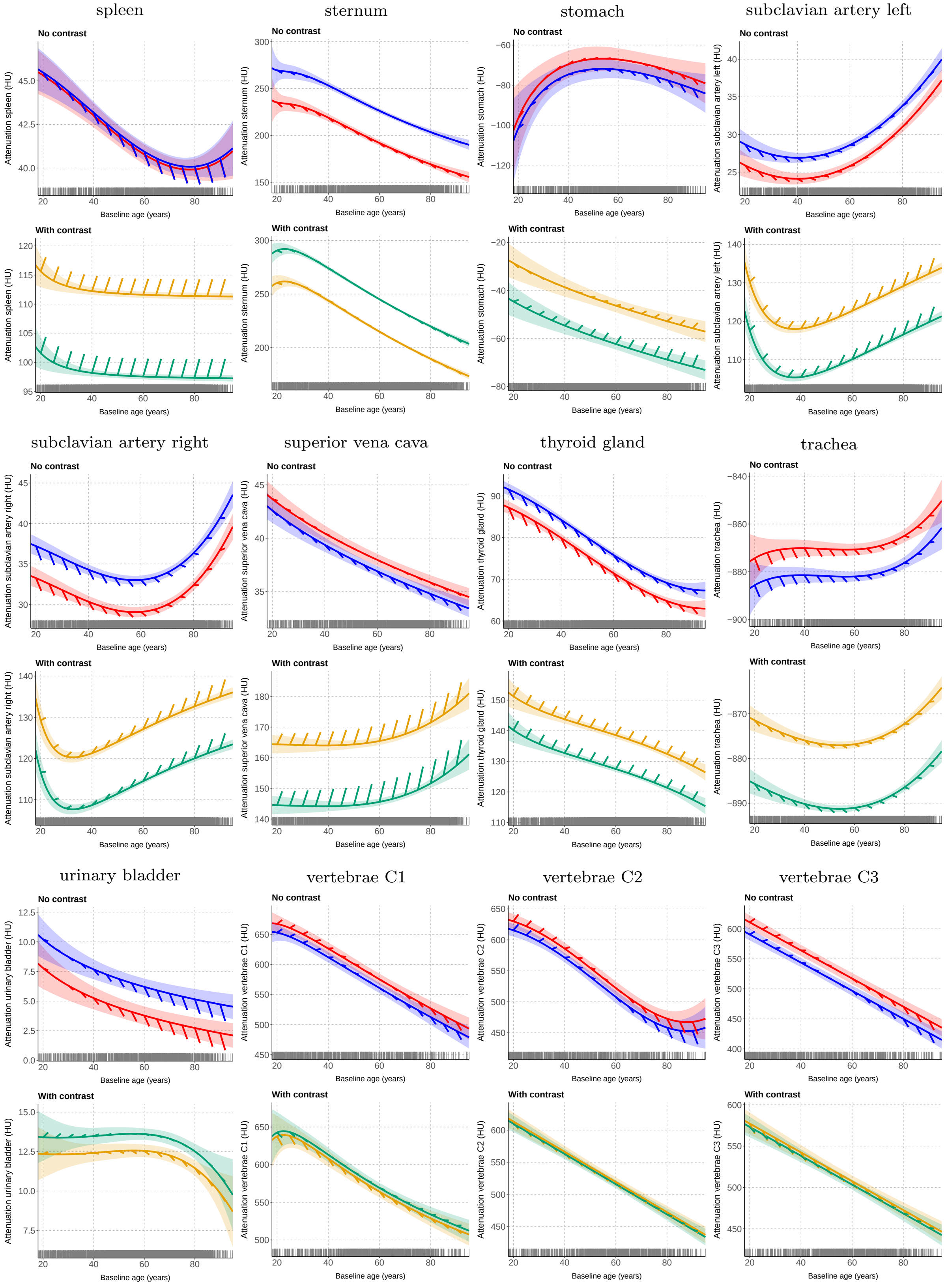
spleen
No contrast
Attenuation spleen (HU)
Baseline age (years)
With contrast
Attenuation spleen (HU)
Baseline age (years)
sternum
No contrast
Attenuation sternum (HU)
Baseline age (years)
With contrast
Attenuation sternum (HU)
Baseline age (years)
stomach
No contrast
Attenuation stomach (HU)
Baseline age (years)
With contrast
Attenuation stomach (HU)
Baseline age (years)
subclavian artery left
No contrast
Attenuation subclavian artery left (HU)
Baseline age (years)
With contrast
Attenuation subclavian artery left (HU)
Baseline age (years)
subclavian artery right
No contrast
Attenuation subclavian artery right (HU)
Baseline age (years)
With contrast
Attenuation subclavian artery right (HU)
Baseline age (years)
superior vena cava
No contrast
Attenuation superior vena cava (HU)
Baseline age (years)
With contrast
Attenuation superior vena cava (HU)
Baseline age (years)
thyroid gland
No contrast
Attenuation thyroid gland (HU)
Baseline age (years)
With contrast
Attenuation thyroid gland (HU)
Baseline age (years)
trachea
No contrast
Attenuation trachea (HU)
Baseline age (years)
With contrast
Attenuation trachea (HU)
Baseline age (years)
urinary bladder
No contrast
Attenuation urinary bladder (HU)
Baseline age (years)
With contrast
Attenuation urinary bladder (HU)
Baseline age (years)
vertebrae C1
No contrast
Attenuation vertebrae C1 (HU)
Baseline age (years)
With contrast
Attenuation vertebrae C1 (HU)
Baseline age (years)
vertebrae C2
No contrast
Attenuation vertebrae C2 (HU)
Baseline age (years)
With contrast
Attenuation vertebrae C2 (HU)
Baseline age (years)
vertebrae C3
No contrast
Attenuation vertebrae C3 (HU)
Baseline age (years)
With contrast
Attenuation vertebrae C3 (HU)
Baseline age (years)

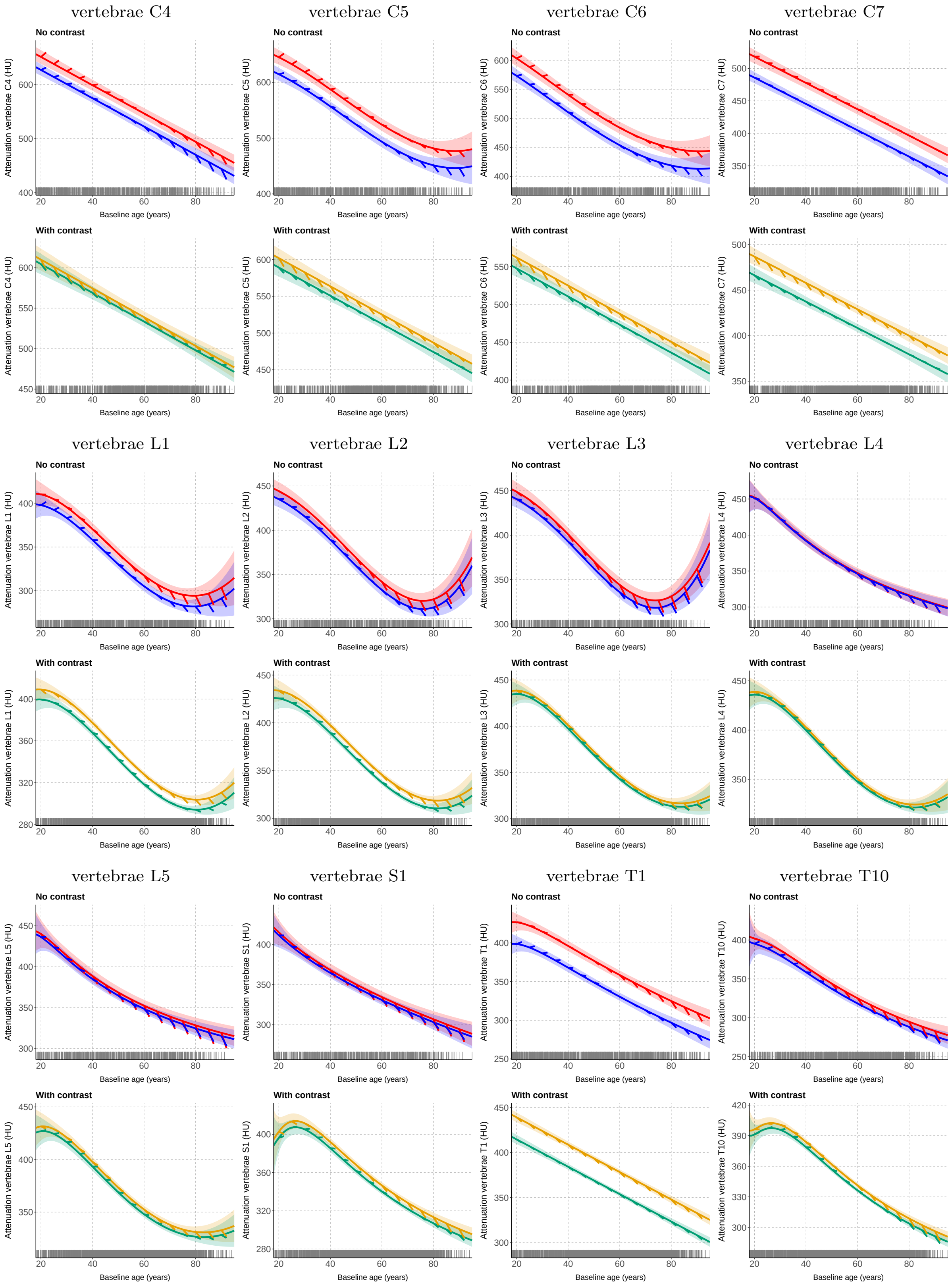
vertebrae C4
vertebrae C5
vertebrae C6
vertebrae C7
vertebrae L1
vertebrae L2
vertebrae L3
vertebrae L4
vertebrae L5
vertebrae S1
vertebrae T1
vertebrae T10
No contrast
With contrast
Baseline age (years)
Attenuation vertebrae C4 (HU)
Attenuation vertebrae C5 (HU)
Attenuation vertebrae C6 (HU)
Attenuation vertebrae C7 (HU)
Attenuation vertebrae L1 (HU)
Attenuation vertebrae L2 (HU)
Attenuation vertebrae L3 (HU)
Attenuation vertebrae L4 (HU)
Attenuation vertebrae L5 (HU)
Attenuation vertebrae S1 (HU)
Attenuation vertebrae T1 (HU)
Attenuation vertebrae T10 (HU)

vertebrae T11

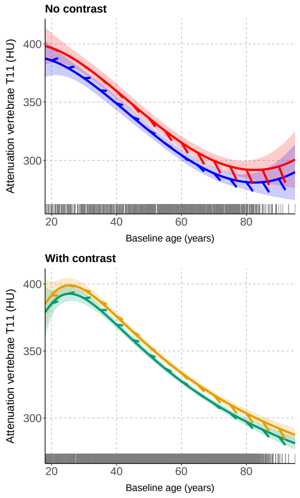

vertebrae T12

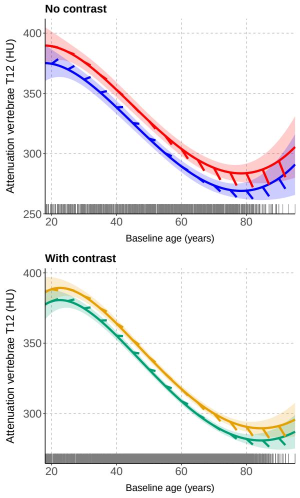

vertebrae T2

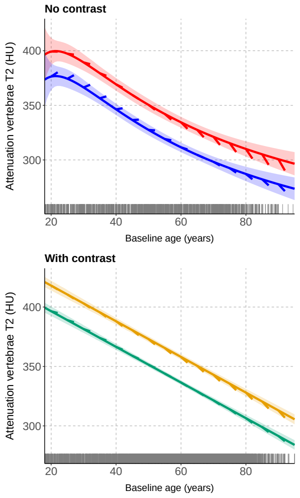

vertebrae T3

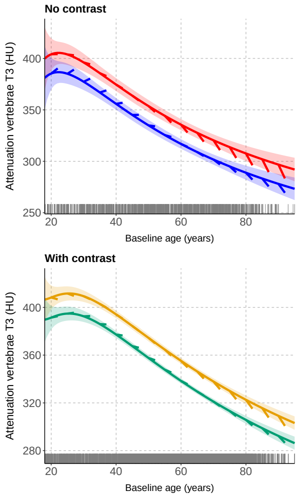

vertebrae T4

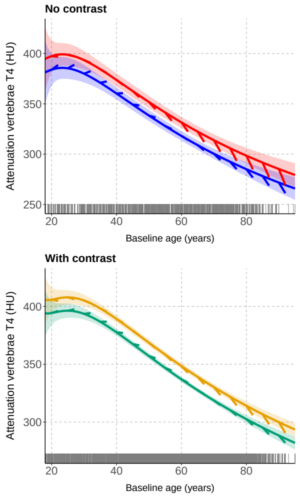

vertebrae T5

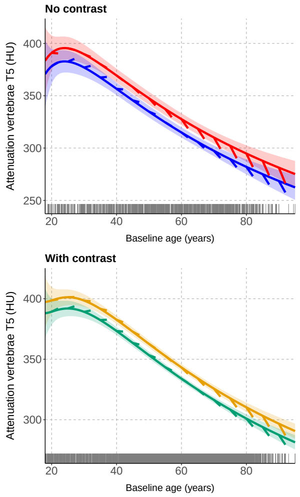

vertebrae T6

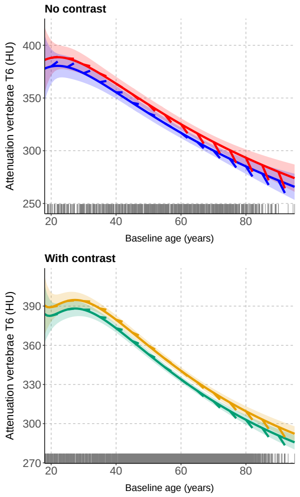

vertebrae T7

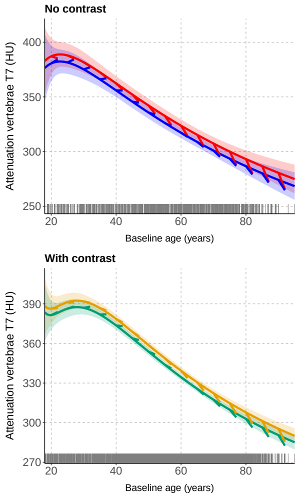

vertebrae T8

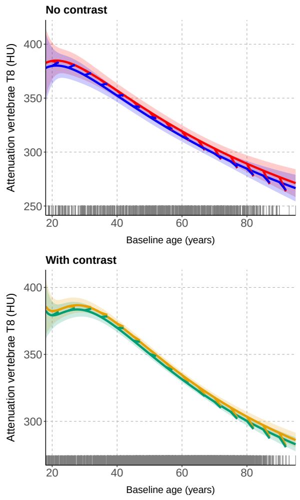

vertebrae T9

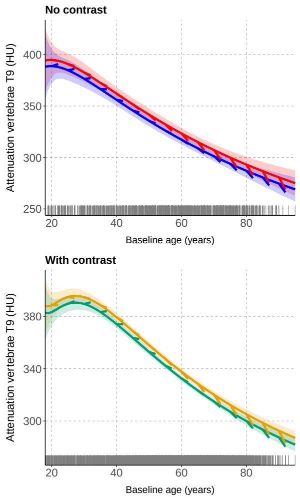

Regression coefficients for cross-sectional volume models

	μ								σ		
Structure	Age-1	Age-2	Sex	kVp×10	Contr	GE	SIE	TOS	Age-1	Age-2	Sex
adrenal gland left	**2.53**	**0.99**	**1.38**	**1.02**	**1.07**	1.00	1.01	0.97	**3.01**		**0.88**
adrenal gland right	**2.21**	**0.99**	**1.40**	**1.03**	**1.11**	1.01	**1.04**	1.00	**2.34**		**0.82**
aorta	**1.04**	**1.00**	**1.34**	1.00	**0.98**	1.06	**0.91**		**0.97**	**1.00**	1.01
atrial appendage left	**1.00**		**1.06**	1.00	**0.97**	0.99	**0.98**	0.99	**1.00**		**1.04**
brachiocephalic trunk	**0.77**	**1.01**	**1.35**	**1.01**	**0.95**	0.99	1.00	**0.92**	**5.35**	**0.07**	**0.94**
brachiocephalic vein left	**0.45**	**1.00**	**1.26**	**1.01**	**1.11**	1.00	**1.01**	1.02	**2.36**	**1.00**	**0.91**
brachiocephalic vein right	**1.03**	**0.99**	**1.23**	**1.02**	**1.02**	0.98	**1.02**	0.99	**1.00**		**1.02**
brain	**1.00**		**1.13**	1.00	**0.98**	0.98	**0.99**				1.01
clavicula left	**1.19**	**1.00**	**1.45**	**1.01**	**0.98**	0.97	**0.99**	0.99	**1.53**		**1.10**
clavicula right	**1.07**	**0.99**	**1.46**	**1.01**	**0.98**	0.99	1.00	1.00	**2.13**		**1.07**
colon	**2.10**	**0.08**	**1.17**	**1.02**	**1.07**	**0.96**	1.00				1.00
common carotid artery left	**1.01**	**1.00**	**1.34**	**1.01**	**1.12**	0.98	1.01	1.01	**0.42**	**1.24**	**0.96**
common carotid artery right	**1.03**	**1.00**	**1.32**	**1.02**	**1.48**	0.92	1.01	0.96	**1.87**	**1.00**	1.01
costal cartilages	**1.07**	**1.00**	**1.57**	1.00	**0.99**	**0.97**	**0.95**	0.97	**1.00**	**1.00**	**1.17**
duodenum	**1.92**	**0.80**	**1.28**	**1.02**	**1.03**	**0.97**	1.00				**0.98**
esophagus	**1.05**	**0.98**	**1.34**	**1.01**	**0.99**	**0.95**	**0.98**	**0.97**			**0.95**
gallbladder	**0.39**		**1.29**	**1.03**	**1.05**	**1.05**	1.02	0.90	**1.00**		**0.95**
gluteus maximus left	**1.00**		**1.35**	**1.04**	**0.97**	0.99	0.99				**1.07**
gluteus maximus right	**0.48**	**1.00**	**1.35**	**1.04**	**0.96**	0.94	1.00				**1.07**
gluteus medius left	**0.72**	**1.00**	**1.34**	**1.01**	**0.99**	**0.96**	**0.99**				**1.03**
gluteus medius right	**1.24**	**0.99**	**1.34**	**1.02**	**0.98**	**0.95**	**0.99**		**1.00**		1.02
gluteus minimus left	**1.96**	**0.86**	**1.34**	**1.01**	**1.01**	**0.98**	**1.01**		**0.55**	**1.14**	**0.97**
gluteus minimus right	**1.65**	**0.60**	**1.34**	**1.01**	1.00	**0.98**	1.00		**0.98**	**1.01**	0.98
heart	**0.79**	**1.00**	**1.30**	**1.01**	**1.01**	**0.94**	**0.97**	**0.95**	**1.00**		**1.04**
hip left	**1.01**	**1.00**	**1.30**	**1.00**	1.00	**1.01**	**1.01**		**0.85**		0.98
hip right	**1.01**	**1.00**	**1.30**	**1.01**	1.00	**1.01**	**1.02**		**0.85**		0.99
iliac artery left	**1.02**	**1.00**	**1.52**	**1.02**	**1.05**	**0.97**	**0.99**		**1.04**		**1.05**
iliac artery right	**1.03**	**0.99**	**1.49**	**1.02**	**0.97**	**0.96**	0.99		**1.00**	**1.00**	**1.04**
iliac vena left	**0.45**	**1.00**	**1.28**	**1.02**	**0.98**	**0.95**	**0.98**		**1.00**		0.99
iliac vena right	**1.64**	**0.89**	**1.31**	**1.02**	**1.02**	**0.95**	**0.98**		**1.60**	**1.00**	0.98
iliopsoas left	**0.10**	**0.00**	**1.56**	**1.01**	**0.97**	**0.96**	**0.98**		**1.00**		**1.02**
iliopsoas right	**0.00**	**0.00**	**1.55**	**1.01**	**0.97**	**0.97**	**0.99**		**1.00**		**1.04**
inferior vena cava	**0.37**	**0.94**	**1.27**	**1.03**	**1.22**	**0.95**	**1.01**	0.97	**0.83**	**1.00**	**0.97**
kidney left	**0.50**	**1.00**	**1.25**	**1.02**	**1.08**	**0.98**	1.00				**0.96**
kidney right	**0.47**	**1.00**	**1.26**	**1.02**	**1.07**	0.99	1.00				**0.95**
liver	**1.57**	**0.99**	**1.18**	**1.01**	**1.02**	**0.96**	**0.97**	0.95	**1.00**		**0.96**
lung lower lobe left	**1.00**	**1.00**	**1.33**	**1.02**	**1.12**	**1.19**	**0.95**	0.98	**1.00**	**1.00**	**1.04**
lung lower lobe right	**1.00**	**1.00**	**1.28**	**1.02**	**1.10**	**1.17**	**0.94**	0.98	**1.00**	**1.00**	**1.04**
lung middle lobe right	**1.01**	**1.00**	**1.41**	**1.01**	**1.05**	**1.09**	**0.96**	0.99			1.01
lung upper lobe left	**1.01**	**1.00**	**1.38**	**1.01**	**1.07**	**1.16**	0.99	1.01	**1.00**	**1.00**	**1.06**
lung upper lobe right	**1.02**	**1.00**	**1.35**	**1.01**	**1.08**	**1.15**	1.00	1.01	**0.91**	**1.00**	1.03
pancreas	**1.62**	**0.82**	**1.26**	**1.01**	**0.98**	**0.94**	**0.98**	0.86	**153.64**	**0.00**	**0.94**
portal vein and splenic vein	**1.60**	**1.00**	**1.38**	**1.04**	**1.72**	**0.96**	**1.03**	1.01	**0.98**	**1.01**	**0.92**
pulmonary vein	**0.67**	**1.03**	**1.29**	**1.01**	**1.10**	**0.91**	**0.97**	**0.93**	**1.36**	**0.20**	1.00
rib left 1	**1.59**	**1.00**	**1.47**	1.00	**1.04**	**0.96**	**0.95**	**0.97**	**0.97**	**1.01**	**0.96**
rib left 10	**1.02**	**1.00**	**1.54**	**1.01**	1.00	0.99	**1.02**	0.99	**0.99**	**1.00**	1.01
rib left 11	**1.00**	**1.00**	**1.56**	**1.01**	1.00	**1.02**	**1.04**	1.03	**0.98**	**1.01**	1.02

	μ								σ		
Structure	Age-1	Age-2	Sex	kVp×10	Contr	GE	SIE	TOS	Age-1	Age-2	Sex
rib left 12	**1.00**	**1.00**	**1.65**	**1.01**	**1.02**	1.01	**1.02**	1.00	**0.92**	**1.00**	**0.89**
rib left 2	**1.00**	**1.00**	**1.45**	**1.00**	1.00	**0.96**	**0.97**	**0.97**	**0.83**	**1.00**	0.99
rib left 3	**0.98**		**1.53**	**1.01**	**1.01**	**0.97**	**0.98**	**0.98**	**1.53**		0.99
rib left 4	0.96		**1.56**	**1.01**	**1.01**	0.98	**0.99**	0.99	**1.51**		**0.97**
rib left 5	**0.83**		**1.57**	**1.01**	1.00	0.99	**0.99**	1.00	**1.70**		**0.98**
rib left 6	**0.76**		**1.55**	**1.01**	1.00	0.99	**0.99**	1.00	**0.87**		**0.96**
rib left 7	**0.83**		**1.58**	**1.01**	0.99	0.99	1.00	1.00	**0.85**		**0.97**
rib left 8	**0.85**		**1.55**	**1.01**	1.00	**0.97**	1.00	0.99	**0.83**		**0.94**
rib left 9	**0.84**		**1.53**	**1.01**	1.01	**0.98**	**1.01**	1.01	**2.22**		**0.95**
rib right 1	**2.86**	**0.83**	**1.43**	1.00	**1.04**	**0.96**	**0.97**	**0.98**	**0.89**	**1.01**	**0.94**
rib right 10	**1.08**	**1.00**	**1.53**	**1.01**	1.00	1.01	**1.03**	1.03	**0.98**	**1.00**	1.01
rib right 11	**1.02**	**1.00**	**1.57**	**1.01**	**1.01**	**1.02**	**1.04**	1.07	**0.99**	**1.00**	1.01
rib right 12	**0.74**		**1.63**	**1.01**	**1.04**	**1.02**	**1.03**	1.03			**0.93**
rib right 2	0.95	**1.02**	**1.45**	**1.00**	**1.01**	**0.96**	**0.97**	**0.97**			0.99
rib right 3	**1.00**		**1.53**	**1.01**	**1.02**	**0.97**	**0.98**	**0.98**	**1.78**		0.98
rib right 4	**0.92**		**1.55**	**1.01**	**1.01**	0.99	**0.99**	1.00	**1.49**		**0.97**
rib right 5	**0.82**		**1.57**	**1.01**	**1.01**	0.99	0.99	1.00	**1.69**		**0.97**
rib right 6	**0.79**		**1.55**	**1.01**	1.00	1.00	1.00	1.01	**0.88**		0.98
rib right 7	**0.83**		**1.56**	**1.01**	1.00	0.99	1.00	1.01	**0.85**		**0.97**
rib right 8	**0.83**		**1.55**	**1.01**	1.01	0.99	**1.01**	1.00	**2.10**		**0.94**
rib right 9	**0.85**		**1.52**	**1.01**	**1.01**	0.99	**1.02**	1.01	**2.07**		**0.96**
sacrum	**1.01**	**1.00**	**1.18**	**1.00**	**1.01**	**1.01**	**1.01**		**0.90**		1.02
scapula left	**1.18**	**1.00**	**1.50**	**1.01**	**1.02**	**0.88**	**0.98**	**0.93**	**0.99**	**1.00**	**1.07**
scapula right	**1.05**	**1.00**	**1.50**	**1.01**	1.01	**0.93**	**0.99**	0.97	**1.00**	**1.00**	**1.05**
small bowel	**1.60**	**1.00**	**1.31**	**1.04**	**1.11**	**0.94**	1.01		**1.93**		**0.91**
spleen	**0.80**	**1.00**	**1.32**	**1.03**	**1.07**	**0.93**	**0.95**	**0.91**	**1.01**	**1.00**	1.02
sternum	**1.23**	**1.00**	**1.49**	**1.01**	**0.99**	0.98	**0.99**	1.00	**0.86**		**1.03**
stomach	**1.07**	**1.00**	**1.25**	**1.03**	**1.07**	**0.95**	1.01	0.96	**1.00**		**1.07**
subclavian artery left	**1.03**	**1.00**	**1.42**	**1.02**	**1.03**	0.98	0.99	**0.97**	**2.01**	**1.00**	**0.96**
subclavian artery right	**0.57**	**1.01**	**1.43**	**1.02**	**1.05**	**0.96**	**0.97**	**0.94**	**2.05**		**0.95**
superior vena cava	**1.02**	**1.00**	**1.23**	**1.01**	**1.03**	**0.92**	1.00	1.00	**1.02**	**1.00**	**1.09**
thyroid gland	**0.20**	**1.00**	**1.46**	**1.01**	**1.17**	0.93	**0.98**	**0.94**	**1.00**	**1.00**	**0.86**
trachea	**1.22**	**1.00**	**1.54**	**1.01**	**1.03**	**1.08**	**0.98**	1.02	**1.97**	**1.00**	0.98
urinary bladder	**1.05**	**0.98**	**1.12**	1.00	**1.18**	**1.07**	1.02		**1.00**		**0.94**
vertebrae C1	**1.01**	**1.00**	**1.31**	**0.99**	1.00	0.98	**1.02**		**1.00**		1.02
vertebrae C2	**1.01**	**0.99**	**1.31**	**0.99**	**1.03**	1.02	**1.01**				**1.05**
vertebrae C3	**1.01**	**1.00**	**1.33**	**0.99**	**1.03**	1.04	**1.01**				1.03
vertebrae C4	**1.01**	**1.00**	**1.33**	**0.99**	**1.02**	1.02	1.00				**1.04**
vertebrae C5	**1.01**	**1.00**	**1.34**	**0.99**	**1.02**	1.03	1.01		**1.00**		1.03
vertebrae C6	**1.02**	**0.99**	**1.33**	**0.99**	1.01	1.00	1.00	0.97	**0.64**		1.03
vertebrae C7	**1.01**	**1.00**	**1.33**	**0.99**	1.00	1.00	1.00	1.01	**1.00**	**1.00**	1.04
vertebrae L1	**1.14**		**1.33**	1.00	1.00	**1.01**	**1.02**	1.01	**1.32**		0.98
vertebrae L2	**1.14**		**1.31**	1.00	1.00	**1.02**	**1.02**		**1.42**		**0.94**
vertebrae L3	**1.14**		**1.29**	1.00	1.01	**1.02**	**1.02**		**1.61**		**0.96**
vertebrae L4	**1.01**	**0.99**	**1.28**	1.00	1.01	**1.03**	**1.03**		**1.44**		1.02
vertebrae L5	**1.01**	**1.00**	**1.26**	1.00	1.01	**1.02**	**1.02**		**1.49**		1.01
vertebrae S1	**1.00**	**1.00**	**1.23**	1.00	**1.01**	0.99	0.99		**1.31**		**1.04**
vertebrae T1	**1.13**		**1.38**	1.00	1.00	1.01	**1.01**	**1.02**	**1.39**		1.01
vertebrae T10	**1.00**	**1.00**	**1.37**	**1.00**	**0.99**	**1.02**	**1.02**	**1.03**	**1.63**		**0.98**
vertebrae T11	**1.01**	**1.00**	**1.36**	**1.00**	1.00	1.01	**1.02**	**1.02**	**0.98**	**1.01**	**0.95**
vertebrae T12	**1.14**		**1.34**	1.00	1.00	1.00	**1.01**	1.02	**1.31**		0.99
vertebrae T2	**1.12**		**1.39**	1.00	1.00	1.01	**1.01**	**1.02**	**1.49**		0.98
vertebrae T3	**1.04**	**1.00**	**1.39**	**1.00**	**0.99**	**1.02**	**1.02**	**1.02**	**1.37**		**0.97**
vertebrae T4	**1.01**	**1.00**	**1.39**	**1.00**	**0.99**	**1.03**	**1.02**	**1.03**	**1.31**		**0.96**
vertebrae T5	**1.01**	**1.00**	**1.39**	**1.00**	**0.99**	**1.03**	**1.02**	**1.03**	**1.45**		0.99
vertebrae T6	**1.01**	**1.00**	**1.40**	**1.01**	**0.99**	**1.03**	**1.02**	**1.03**	**1.42**		**0.94**

	μ								σ		
Structure	Age-1	Age-2	Sex	kVp×10	Contr	GE	SIE	TOS	Age-1	Age-2	Sex
vertebrae T7	**1.00**	**1.00**	**1.39**	**1.01**	**0.99**	1.01	**1.02**	**1.03**	**1.47**		**0.91**
vertebrae T8	**1.00**	**1.00**	**1.38**	**1.00**	**0.99**	1.00	**1.01**	**1.02**	**1.45**		**0.91**
vertebrae T9	**1.00**	**1.00**	**1.37**	**1.00**	**0.99**	1.01	**1.01**	**1.02**	**1.60**		**0.95**

Supplementary Table 4: GAMLSS coefficient estimates for each anatomical structure. Columns are grouped by distribution parameters (μ and σ). For μ, estimates represent multiplicative effects on the location parameter: Age terms (Age-1, Age-2; fractional-polynomial basis coefficients), Sex (male vs female), kVp (per 10 kVp increase), Contrast (contrast-enhanced vs non-contrast), and manufacturer indicators (GE, Siemens, Toshiba vs the reference manufacturer). For σ, estimates analogously summarize effects on the scale parameter (Age-1, Age-2; Sex). Estimates are shown as $\exp(\hat{\beta})$; values > 1 indicate an increase and values < 1 a decrease in the respective parameter relative to the reference. Boldface marks Bonferroni-adjusted $p < 0.05$. A third age fractional-polynomial term for μ was selected for: brachiocephalic trunk (1.00), brachiocephalic vein left (1.00), iliopsoas left (973.92), iliopsoas right (6.2e+06), subclavian artery right (1.00), and urinary bladder (1.01). A third age fractional-polynomial term for σ was selected for: prostate (0.98).

Regression coefficients for cross-sectional attenuation models

		μ								σ		
Structure	Cont	Age-1	Age-2	Age-3	Sex	kVp×10	GE	SIE	TOS	Age-1	Age-2	Sex
adrenal gland left	×	**-115**	**29**		**-6**	**1**	2	**-1**		**88.85**	**2.86**	1.02
adrenal gland left	✓	**66**	**0**		**-11**	**-4**	**-2**	**-5**	0	**1.00**	**1.00**	**0.88**
adrenal gland right	×	**-335**	**677**	**-452**	**-6**	**1**	7	0		**0.85**	**1.14**	0.97
adrenal gland right	✓	**59**	0		**-11**	**-3**	**2**	**-1**	**4**	**1.00**	**1.00**	**0.88**
aorta	×	**0**	**0**		0	**0**		**3**		**1.00**		**1.20**
aorta	✓	**-14**	**6**		**-18**	**-26**	-13	**-16**		**0.99**	**1.00**	**0.83**
atrial appendage left	×	**-11**	**0**		**1**	**0**	3	**1**		**1.00**	**1.00**	**1.13**
atrial appendage left	✓	**-3**	**1**		**-15**	**-20**	**-5**	**-14**	**-50**	**0.99**	**1.00**	**0.93**
brachiocephalic trunk	×	**-3**	**0**		0	**1**	5	**2**		**0.08**	**2.09**	1.04
brachiocephalic trunk	✓	**-1**	**1**	**0**	**-15**	**-26**	**14**	**-10**	**-78**	**1.00**	**1.00**	**0.88**
brachiocephalic vein left	×	**-2**	**0**		0	**0**	-1	**2**				**1.08**
brachiocephalic vein left	✓	**0**			0	0	0	0	**-8**			**0.83**
brachiocephalic vein right	×	**20**			**-1**	**1**	9	**8**				**1.10**
brachiocephalic vein right	✓	**1**	**0**		**-11**	-1	-5	**-10**	**-42**	**0.97**	**1.00**	**0.89**
brain	×	**0**	**0**		0	**-3**	**6**	**10**		**1.06**	**0.99**	1.02
brain	✓	**4**	**-2**		**-2**	**-2**	4	**1**				0.92
clavicula left	×	**201**	**-110**		**-8**	**-18**		**-12**		**1.11**		1.05
clavicula left	✓	**2356**	**-4930**	**5723**	8	**-36**	**-49**	**-68**	**-15**	1.00		0.98
clavicula right	×	**-1668**	**-535**		**-12**	**-17**		**-13**		**0.69**		1.02
clavicula right	✓	**2080**	**-4227**	**5180**	4	**-33**	**-54**	**-62**	**-19**	1.00		0.97
colon	×	**-186**			0	7	45	7		**1.00**		0.98
colon	✓	**-204**	**0**		**-12**	3	**-9**	0				1.00
common carotid artery left	×	**-1**	**0**		0	0	8	**5**				1.03
common carotid artery left	✓	36	**0**		**-11**	**-15**	19	**-9**	8	**0.99**	**1.00**	**0.82**
common carotid artery right	×	**0**	**0**		1	0	10	**5**		**1.00**		1.02
common carotid artery right	✓	13	0		**-11**	**-13**	24	**-8**	**-62**	**0.99**	**1.00**	**0.79**
costal cartilages	×	**36**	**-6**	**0**	**10**	0		**-6**		**1.00**		**0.89**
costal cartilages	✓	**45**	**-13**	**4**	**9**	**-3**	**-13**	**-13**	-1	**1.00**		**0.90**
duodenum	×	**11**			**-3**	0	2	1		**1.00**		**0.78**
duodenum	✓	**-1**	**0**		**-10**	**-4**	**-5**	**-7**		**3.76**	**1.00**	**0.84**
esophagus	×	**-6**			-1	0	-12	**6**		**1.00**		1.00
esophagus	✓	**0**			**-3**	-1	0	2	**-8**	**1.00**		0.97
gallbladder	×	**20**			**-3**	0	1	0		**1.00**		0.97
gallbladder	✓	**-1**	**0**		**-3**	**-1**	**2**	**-3**	**4**	**1.00**		**0.95**
gluteus maximus left	×	**67**			2	2		2		**1.05**		0.88
gluteus maximus left	✓	**-18**			**3**	**-3**	**6**	**-4**		**0.35**		**0.90**
gluteus maximus right	×	**64**			2	1		2		**1.01**		0.92
gluteus maximus right	✓	**-17**			**2**	**-3**	**11**	**-4**		**0.34**		0.93
gluteus medius left	×	**14**	**0**		**2**	0	**8**	0		**1.01**	**1.00**	**0.88**
gluteus medius left	✓	**-6**	**0**		0	**-3**	**7**	**-5**		**1.12**		**0.93**
gluteus medius right	×	**14**	**0**		**1**	0	**8**	1		**1.01**	**1.00**	**0.89**
gluteus medius right	✓	**23**	**0**		**-1**	**-3**	**7**	**-5**		**1.12**		**0.92**
gluteus minimus left	×	**-23**	**8**	**-3**	**3**	**-1**	**13**	**3**		**0.10**	**2.17**	**0.75**
gluteus minimus left	✓	**-3**	**0**	**0**	**1**	**-3**	**12**	0		**0.99**	**1.02**	**0.83**

		μ								σ		
Structure	Cont	Age-1	Age-2	Age-3	Sex	kVp×10	GE	SIE	TOS	Age-1	Age-2	Sex
gluteus minimus right	×	**-21**	**7**	**-3**	**3**	**-1**	**11**	**3**		**9.10**	**1.03**	**0.76**
gluteus minimus right	✓	**9**	**0**		**2**	**-3**	**12**	0		**1.01**	**1.00**	**0.84**
heart	×	**0**	**0**		**2**	0		**0**		**239.76**	**0.03**	**1.08**
heart	✓	**-1**	**1**		**-15**	**-24**	**-11**	**-15**	**-29**	**0.99**	**1.00**	**0.84**
hip left	×	**-27**			3	**-15**	-14	**-40**				0.98
hip left	✓	**-3637**	**-6853**	**5213**	**-7**	**-19**	**-31**	**-47**		**1.00**		1.01
hip right	×	**-26**			0	**-16**	-15	**-38**				0.97
hip right	✓	**-3656**	**-6846**	**5220**	**-10**	**-19**	**-28**	**-46**		**1.00**		1.01
iliac artery left	×	-7	**0**	**0**	**3**	**-2**	-3	0		**0.75**	**1.13**	1.10
iliac artery left	✓	**-8**	**1**		**-4**	**-12**	**-17**	**-21**		**1.00**		**1.07**
iliac artery right	×	**3**	**-3**	**1**	**3**	**-2**	-4	**-1**		**0.73**	**1.35**	1.10
iliac artery right	✓	**-8**	**1**		**-5**	**-12**	**-17**	**-21**		**1.00**		**1.05**
iliac vena left	×	**0**	**0**		**3**	**-2**	3	0		**1.01**	**0.99**	**1.08**
iliac vena left	✓	**-4**			**-10**	**-2**	**-5**	**-2**				**0.85**
iliac vena right	×	**0**	**0**		**3**	**-1**	2	0		**1.01**	**0.99**	1.06
iliac vena right	✓	**-4**			**-11**	**-2**	**-4**	**-2**		**1.00**		**0.84**
iliopsoas left	×	**0**	**0**		**1**	**-1**	4	0		**1.01**	**0.99**	0.99
iliopsoas left	✓	**0**	**0**		**-1**	**-2**	**5**	**-2**		**1.07**		**0.96**
iliopsoas right	×	**0**	**0**		**2**	**-1**	5	0		**1.02**	**0.99**	1.03
iliopsoas right	✓	**-1**	**0**		**-1**	**-2**	**7**	**-1**		**1.07**		**0.96**
inferior vena cava	×	**-5**			0	0	2	**2**		**0.47**		**1.21**
inferior vena cava	✓	**-7**			**-14**	**-6**	**-13**	**-11**	-30	**1.00**		**0.90**
kidney left	×	**-33**	**98**		**-1**	**0**	6	**1**		**1.05**	**1.00**	**1.35**
kidney left	✓	**-14**			**-15**	**-13**	**-15**	**-24**		**1.00**		**0.92**
kidney right	×	**-25**	**85**		**-1**	0	5	1		**1.01**	**0.99**	**1.42**
kidney right	✓	**-15**			**-16**	**-13**	**-13**	**-23**		**1.00**		**0.92**
liver	×	-10	**55**		**-2**	**1**	5	**3**		**1.03**	**1.00**	1.04
liver	✓	**35**			**-9**	**-5**		**-6**	4	**1.00**		**0.92**
lung lower lobe left	×	33			**-8**	**-3**		**-9**		**1.00**		1.04
lung lower lobe left	✓	**0**	**0**		**-14**	**-4**	**-50**	**15**	-3	**0.99**	**1.00**	1.04
lung lower lobe right	×	26			**-8**	**-3**		**-8**		**1.00**		1.06
lung lower lobe right	✓	**0**	**0**		**-13**	**-4**	**-45**	**14**	-8	**0.99**	**1.00**	1.07
lung middle lobe right	×	0			**-10**	**-2**		**-7**				0.97
lung middle lobe right	✓	**0**	**0**		**-19**	**-3**	**-21**	**8**	-6	**0.99**	**1.00**	0.98
lung upper lobe left	×	**35**			**-8**	**-3**		**-6**				0.97
lung upper lobe left	✓	**0**	**0**		**-17**	**-4**	**-26**	**11**	-7	**0.99**	**1.00**	0.99
lung upper lobe right	×	**54**			**-7**	**-3**		**-5**				0.98
lung upper lobe right	✓	**0**	**0**		**-14**	**-4**	**-23**	**11**	-7	**0.99**	**1.00**	1.00
pancreas	×	**-103**	**-39**		**-3**	**-1**	3	**-1**		**1.06**	**0.99**	**1.22**
pancreas	✓	**-2**	**1**		**-12**	**-7**	**-4**	**-11**	9	**1.07**		**0.94**
portal vein and splenic vein	×	**0**	**0**		0	0	-1	**-3**		**479.78**	**0.00**	1.02
portal vein and splenic vein	✓	**22**			**-14**	**-9**	**-22**	**-23**	**-19**	**1.00**		**0.92**
prostate	×	**88**	**-53**		-2	0	13	**5**		**1.08**		0.76
prostate	✓	**-36**	**139**		**-3**	**-3**	**4**	**-3**		**0.97**	**1.00**	**0.73**
pulmonary vein	×	**0**			**0**	**1**	4	**7**		**1.62**		**1.08**
pulmonary vein	✓	**-1**	**0**		**-17**	**-30**	**-16**	**-21**	**-43**	**1.00**	**1.00**	**0.88**
rib left 1	×	**-4584**	**-7920**	**6248**	**-16**	**-7**		**9**		**0.00**	**807.70**	0.97
rib left 1	✓	**135**	**-87**		**-8**	**-32**	**-49**	**-65**	**-22**	**0.87**	**1.01**	**0.92**
rib left 10	×	**-6**	**0**		**-26**	**-7**	-8	**-17**				**0.94**
rib left 10	✓	**1961**	**-3453**	**4024**	**-20**	**-25**	**-87**	**-97**	**-66**	**2.5e+08**	**0.00**	0.98
rib left 11	×	**-2**	**1**		**-19**	**-6**	-32	**-32**				**0.95**
rib left 11	✓	**1970**	**-3380**	**4339**	**-20**	**-26**	**-77**	**-92**	**-62**	**1417.60**	**0.01**	0.99
rib left 12	×	**-9**	**1**		**-19**	**-3**	-31	**-26**		**1.00**		**0.87**
rib left 12	✓	**1946**	**-3288**	**4260**	**-26**	**-25**	**-63**	**-83**	**-65**	**13.07**	**0.17**	**0.93**

		μ								σ		
Structure	Cont	Age-1	Age-2	Age-3	Sex	kVp×10	GE	SIE	TOS	Age-1	Age-2	Sex
rib left 2	×	**-14**	**5**		**-37**	**-7**		3				**0.90**
rib left 2	✓	**-9**	**7**	**-2**	**-20**	**-20**	**-60**	**-68**	**-47**	**1.00**	**1.00**	**0.91**
rib left 3	×	**-17**	**6**		**-39**	**-7**		-2		**0.98**		**0.91**
rib left 3	✓	**-10**	**8**	**-2**	**-25**	**-17**	**-65**	**-75**	**-48**	**13.84**	**0.16**	**0.93**
rib left 4	×	**-17**	**6**		**-43**	**-7**		-4				**0.92**
rib left 4	✓	**-11**	**9**	**-2**	**-22**	**-15**	**-66**	**-81**	**-50**	**11.64**	**0.18**	**0.94**
rib left 5	×	**-16**	**6**		**-44**	**-7**		-4				**0.92**
rib left 5	✓	**-10**	**8**	**-2**	**-23**	**-15**	**-67**	**-84**	**-54**	**1.00**	**1.00**	**0.93**
rib left 6	×	**-12**	**4**		**-44**	**-8**		**-5**				**0.92**
rib left 6	✓	**-8**	**6**	**-1**	**-22**	**-15**	**-71**	**-87**	**-58**	**1.00**	**1.00**	**0.93**
rib left 7	×	**-11**	**4**		**-43**	**-8**	-2	**-7**				**0.90**
rib left 7	✓	**-7**	**6**	**-1**	**-17**	**-16**	**-77**	**-91**	**-63**	**1.00**	**1.00**	**0.92**
rib left 8	×	**-12**	**4**		**-38**	**-8**	21	**-5**				**0.91**
rib left 8	✓	**-7**	**6**	**-1**	**-15**	**-19**	**-83**	**-90**	**-61**	**1.00**	**1.00**	**0.93**
rib left 9	×	**-29**			**-30**	**-7**	-20	**-7**				**0.93**
rib left 9	✓	**-126**			**-13**	**-22**	**-84**	**-90**	**-67**	**0.94**		**0.95**
rib right 1	×	**34**	**-34**	**8**	**-15**	**-8**		**11**		**0.00**	**1.8e+05**	0.99
rib right 1	✓	**-1290**	**-437**		**-6**	**-31**	**-55**	**-61**	**-22**	**2.31**		**0.91**
rib right 10	×	**-28**			**-24**	**-8**	-18	**-15**				**0.95**
rib right 10	✓	**1902**	**-3378**	**3883**	**-18**	**-24**	**-82**	**-95**	**-62**	**6014.22**	**0.01**	0.98
rib right 11	×	**-8**	**1**		**-13**	**-8**	-18	**-37**		**1.00**		**0.92**
rib right 11	✓	**1922**	**-3305**	**4207**	**-16**	**-26**	**-77**	**-95**	**-59**	**8.59**	**0.23**	0.99
rib right 12	×	**-9**	**1**		**-19**	**-3**	-22	**-30**		**1.00**		**0.89**
rib right 12	✓	**1815**	**-3015**	**4033**	**-23**	**-25**	**-64**	**-83**	**-71**	**12.70**	**0.17**	**0.94**
rib right 2	×	**-40**	**0**		**-37**	**-8**		3				**0.90**
rib right 2	✓	**-9**	**7**	**-1**	**-20**	**-20**	**-57**	**-67**	**-44**	**1.00**	**1.00**	**0.91**
rib right 3	×	**-17**	**6**		**-40**	**-7**		0		**0.98**		**0.90**
rib right 3	✓	**-10**	**8**	**-2**	**-25**	**-16**	**-61**	**-74**	**-46**	**3.0e+05**	**0.00**	**0.92**
rib right 4	×	**-17**	**6**		**-43**	**-7**		-2		**1.00**		**0.91**
rib right 4	✓	**-10**	**8**	**-2**	**-25**	**-15**	**-63**	**-80**	**-48**	**1.00**	**1.00**	**0.93**
rib right 5	×	**-15**	**5**		**-44**	**-8**		-3				**0.92**
rib right 5	✓	**-9**	**7**	**-1**	**-23**	**-15**	**-64**	**-83**	**-49**	**1.00**	**1.00**	**0.93**
rib right 6	×	**-13**	**5**		**-47**	**-8**		-4				**0.91**
rib right 6	✓	**-8**	**6**	**-1**	**-25**	**-15**	**-67**	**-86**	**-54**	**1.00**	**1.00**	**0.92**
rib right 7	×	**-11**	**4**		**-42**	**-8**	5	**-6**				**0.90**
rib right 7	✓	**-7**	**6**	**-1**	**-18**	**-16**	**-70**	**-89**	**-60**	**1.00**	**1.00**	**0.92**
rib right 8	×	**-11**	**4**		**-37**	**-8**	19	-5				**0.91**
rib right 8	✓	**-6**	**5**	**-1**	**-16**	**-18**	**-72**	**-88**	**-56**	**1.00**	**1.00**	**0.93**
rib right 9	×	**-28**			**-31**	**-8**	-6	**-7**				**0.92**
rib right 9	✓	**1911**	**-3435**	**3900**	**-16**	**-21**	**-76**	**-89**	**-61**	**0.95**		**0.95**
sacrum	×	**-19**			**15**	**-3**	-12	**-24**				**1.07**
sacrum	✓	**1535**	**-2991**	**3361**	**13**	**-13**	**-13**	**-32**		1.00		**1.08**
scapula left	×	**-1032**	**-329**		**-7**	**-9**		**-17**				1.05
scapula left	✓	**110**	**-64**		-2	**-29**	-5	**-52**	-3			1.03
scapula right	×	**828**	**794**		-5	**-10**		**-15**				1.02
scapula right	✓	**-1289**	**-400**		**-6**	**-29**	**-21**	**-56**	-7	**1.09**		1.03
small bowel	×	**0**			-3	2	11	1		**1.01**		0.97
small bowel	✓	**-1**			**-15**	0	**-19**	**-13**		**1.00**		1.02
spleen	×	**0**	**0**		**1**	0	**6**	**2**		**1.01**	**1.00**	**1.14**
spleen	✓	**17**			**-8**	**-7**	**-10**	**-20**	**-8**	**1.00**		**0.90**
sternum	×	**-2195**	**-3879**	**3010**	**20**	**-6**	34	2		**1.00**	**1.00**	**1.10**
sternum	✓	**581**	**508**		**27**	**-9**	**-33**	**-30**	**-23**			**1.12**
stomach	×	**68**	**7**		-2	1	27	1		**0.16**	**1.91**	0.99
stomach	✓	**16**			**-15**	-1	-2	**-7**	-9	**1.00**		**1.06**
subclavian artery left	×	**-2**	**0**		**2**	**1**	6	**7**		**0.16**	**1.55**	0.98
subclavian artery left	✓	**-42**	**-65**		**-9**	**-23**	**-8**	**-15**	**-87**	**0.98**	**1.00**	**0.88**

		μ								σ		
Structure	Cont	Age-1	Age-2	Age-3	Sex	kVp×10	GE	SIE	TOS	Age-1	Age-2	Sex
subclavian artery right	×	**0**	**0**		**3**	**1**	8	**5**		**0.96**	**1.02**	1.00
subclavian artery right	✓	8	**-74**		**-9**	**-24**	-1	**-15**	**-90**	**0.99**	**1.00**	**0.86**
superior vena cava	×	**-4**			0	**0**	-2	**4**				**1.10**
superior vena cava	✓	0	0		**-19**	**-25**	**-31**	**-15**	**-34**	**0.99**	**1.00**	**0.92**
thyroid gland	×	**-1**	**0**		**2**	**-3**	4	-1				0.99
thyroid gland	✓	**44**	**0**		**-5**	**-7**	-5	**-13**	**-34**	**1.01**		**0.80**
trachea	×	**30**	**0**	**0**	**-10**	**-1**		**-14**		**0.14**	**3.50**	**0.85**
trachea	✓	-1	0		**-13**	**2**	**34**	**33**	**35**	**1.00**	**1.00**	**0.94**
urinary bladder	×	**-4**			**3**	0	2	1		**1.00**		**0.84**
urinary bladder	✓	**-1**	**0**	**0**	**2**	**-2**	**3**	0	0	5628.07	**0.01**	**0.83**
vertebrae C1	×	**97**	**-77**		**-17**	**-31**	30	**-27**		**1.21**		0.98
vertebrae C1	✓	**-1344**	**1775**		5	**-41**	13	**-28**				0.98
vertebrae C2	×	**-2**	**1**		**-22**	**-28**	11	**-24**		**1.23**		0.97
vertebrae C2	✓	**-26**			0	**-34**	13	**-22**				0.99
vertebrae C3	×	**-25**			**-23**	**-25**	7	**-21**		**0.42**		0.95
vertebrae C3	✓	**-22**			0	**-33**	14	**-18**		**0.47**		0.99
vertebrae C4	×	**-28**			**-30**	**-23**		**-30**		**0.39**		0.96
vertebrae C4	✓	**-23**			-4	**-36**	12	**-21**		**0.48**		0.98
vertebrae C5	×	**-7**	**1**		**-34**	**-19**		**-38**		**0.42**		**0.92**
vertebrae C5	✓	**-23**			**-10**	**-33**	0	**-22**	-23			0.95
vertebrae C6	×	**-12**	**4**		**-38**	**-13**		**-43**		**0.44**		0.93
vertebrae C6	✓	**-22**			**-13**	**-30**	-12	**-26**	-23			0.95
vertebrae C7	×	**-21**			**-35**	**-6**		**-30**		**1.17**		**0.92**
vertebrae C7	✓	**-18**			**-14**	**-28**	-9	**-29**	2			0.95
vertebrae L1	×	**132**	**-49**	**16**	**-17**	**-3**	-9	**-16**		**1.00**		0.94
vertebrae L1	✓	**123**	**-47**	**15**	**-13**	**-21**	**-29**	**-41**	-25	**1.00**		0.96
vertebrae L2	×	**-2**	**1**		**-17**	-1	-17	**-47**		**1.00**		0.93
vertebrae L2	✓	**123**	**-47**	**15**	**-12**	**-22**	**-31**	**-46**	-41	**1.00**		0.97
vertebrae L3	×	**-2**	**1**		**-15**	-1	-19	**-54**		**1.01**		**0.90**
vertebrae L3	✓	**202**	**-149**	**16**	**-9**	**-23**	**-34**	**-48**		**1.00**		0.98
vertebrae L4	×	**133**	**825**		**-13**	-3	-4	**-54**		**1.01**		**0.90**
vertebrae L4	✓	**189**	**-139**	**15**	**-9**	**-23**	**-36**	**-49**		**1.00**		0.99
vertebrae L5	×	**139**	**724**		**-14**	**-4**	-15	**-53**		**1.06**		**0.91**
vertebrae L5	✓	**391**	**-286**	**57**	**-11**	**-24**	**-37**	**-52**		**1.00**		0.99
vertebrae S1	×	**-349**	**-168**		**-12**	-2	-16	**-40**		**1.07**		**0.91**
vertebrae S1	✓	**1438**	**-2915**	**3481**	**-11**	**-22**	**-27**	**-47**		**1.03**		0.97
vertebrae T1	×	**-1011**	**-327**		**-29**	**-3**		**15**		**1.02**		**0.94**
vertebrae T1	✓	**-18**			**-15**	**-23**	6	**-24**	6			**0.95**
vertebrae T10	×	**-2765**	**-4422**	**3674**	**-18**	**-6**	15	**13**		**1.04**		0.97
vertebrae T10	✓	**1386**	**-2719**	**3210**	**-9**	**-15**	**-36**	**-39**	**-26**	**1.13**		0.99
vertebrae T11	×	**114**	**-42**	**13**	**-18**	**-5**	0	**12**		**1.00**		0.97
vertebrae T11	✓	**1160**	**-2227**	**2887**	**-11**	**-17**	**-33**	**-39**	**-23**	**1.00**		0.98
vertebrae T12	×	**131**	**-48**	**15**	**-17**	**-5**	-4	**7**		**1.00**		0.95
vertebrae T12	✓	**163**	**-119**	**12**	**-13**	**-18**	**-29**	**-37**	**-21**	**1.00**		**0.96**
vertebrae T2	×	**-2409**	**-4184**	**3323**	**-30**	**-7**		**21**		**1.03**		**0.95**
vertebrae T2	✓	**-17**			**-14**	**-19**	**-12**	**-27**	**-12**			**0.96**
vertebrae T3	×	**-2753**	**-4842**	**3809**	**-25**	**-8**		**17**		**1.14**		0.97
vertebrae T3	✓	**-2363**	**-4080**	**3306**	**-11**	**-17**	**-14**	**-31**	**-18**	**0.52**		0.98
vertebrae T4	×	**-3002**	**-5343**	**4159**	**-20**	**-7**		**15**		**1.13**		0.98
vertebrae T4	✓	**-2529**	**-4404**	**3546**	**-7**	**-15**	**-12**	**-32**	**-18**	**0.50**		1.00
vertebrae T5	×	**-700**	**-3031**	**1968**	**-19**	**-7**		**16**		**1.16**		0.98
vertebrae T5	✓	**-2578**	**-4468**	**3593**	**-6**	**-14**	**-13**	**-34**	**-22**	**0.48**		1.02
vertebrae T6	×	**4096**	**-3020**	**3192**	**-17**	**-7**		**16**		**1.17**		0.99
vertebrae T6	✓	**1487**	**-2941**	**3299**	**-4**	**-13**	**-17**	**-36**	**-26**	**0.70**		1.02
vertebrae T7	×	**4030**	**-2970**	**3138**	**-17**	**-7**		**15**		**1.18**		0.97
vertebrae T7	✓	**1489**	**-2960**	**3307**	**-4**	**-13**	**-23**	**-37**	**-29**	**0.67**		1.02

		μ								σ		
Structure	Cont	Age-1	Age-2	Age-3	Sex	kVp×10	GE	SIE	TOS	Age-1	Age-2	Sex
vertebrae T8	×	**4097**	**-3024**	**3169**	**-16**	**-7**		**14**		**1.05**		0.98
vertebrae T8	✓	**1470**	**-2919**	**3252**	-3	**-13**	**-31**	**-37**	**-30**	**0.60**		1.01
vertebrae T9	×	**3576**	**-2628**	**2890**	**-18**	**-7**	-2	**13**		**1.04**		0.97
vertebrae T9	✓	**1441**	**-2842**	**3249**	**-7**	**-14**	**-36**	**-38**	**-29**	**0.59**		1.00

Supplementary Table 5: GAMLSS(ST1) coefficient estimates for intensity models, stratified by contrast status (Cont). Bold entries indicate Bonferroni-adjusted $p < 0.05$. For μ (identity link), coefficients are additive effects on intensity; the kVp coefficient is scaled to represent the effect per 10 kVp. For σ (log link), coefficients are reported as $\exp(\beta)$ and represent multiplicative effects on the scale parameter. Age terms (Age-1 to Age-3) are fractional-polynomial basis coefficients from FP(Age). Manufacturer effects (GE/SIEMENS/TOSHIBA) are relative to the reference manufacturer level (Philips).

Fractional polynomials for age in volume GAMLSS

Structure	$\mathrm{FP}_\mu(\text{age})$	$\mathrm{FP}_\sigma(\text{age})$
adrenal gland left	$\beta_{\mu 1} \cdot x^{0.5} + \beta_{\mu 2} \cdot x^2$	$\beta_{\sigma 1} \cdot x^{-2}$
adrenal gland right	$\beta_{\mu 1} \cdot log(x) + \beta_{\mu 2} \cdot x^2$	$\beta_{\sigma 1} \cdot x^{-1}$
aorta	$\beta_{\mu 1} \cdot x^2 + \beta_{\mu 2} \cdot x^3$	$\beta_{\sigma 1} \cdot x^2 + \beta_{\sigma 2} \cdot x^3$
atrial appendage left	$\beta_{\mu 1} \cdot x^3$	$\beta_{\sigma 1} \cdot x^2$
brachiocephalic trunk	$\beta_{\mu 1} \cdot x^{-2} + \beta_{\mu 2} \cdot x^3 + \beta_{\mu 3} \cdot x^3 \log(x)$	$\beta_{\sigma 1} \cdot x^{-2} + \beta_{\sigma 2} \cdot x^{-2} \log(x)$
brachiocephalic vein left	$\beta_{\mu 1} \cdot x^{-2} + \beta_{\mu 2} \cdot x^3 + \beta_{\mu 3} \cdot x^3 \log(x)$	$\beta_{\sigma 1} \cdot x^{-2} + \beta_{\sigma 2} \cdot x^2$
brachiocephalic vein right	$\beta_{\mu 1} \cdot x^2 + \beta_{\mu 2} \cdot x^2 \log(x)$	$\beta_{\sigma 1} \cdot x^3$
brain	$\beta_{\mu 1} \cdot x^2$	
clavicula left	$\beta_{\mu 1} \cdot x^{0.5} + \beta_{\mu 2} \cdot x^2$	$\beta_{\sigma 1} \cdot x^{-2}$
clavicula right	$\beta_{\mu 1} \cdot x^1 + \beta_{\mu 2} \cdot x^2$	$\beta_{\sigma 1} \cdot x^{-2}$
colon	$\beta_{\mu 1} \cdot x^{-2} + \beta_{\mu 2} \cdot x^{-2} \log(x)$	
common carotid artery left	$\beta_{\mu 1} \cdot x^3 + \beta_{\mu 2} \cdot x^3 \log(x)$	$\beta_{\sigma 1} \cdot x^{0.5} + \beta_{\sigma 2} \cdot x^1$
common carotid artery right	$\beta_{\mu 1} \cdot x^2 + \beta_{\mu 2} \cdot x^3$	$\beta_{\sigma 1} \cdot x^{-0.5} + \beta_{\sigma 2} \cdot x^3$
costal cartilages	$\beta_{\mu 1} \cdot x^1 + \beta_{\mu 2} \cdot x^3$	$\beta_{\sigma 1} \cdot x^3 + \beta_{\sigma 2} \cdot x^3 \log(x)$
duodenum	$\beta_{\mu 1} \cdot x^1 + \beta_{\mu 2} \cdot x^1 \log(x)$	
esophagus	$\beta_{\mu 1} \cdot x^2 + \beta_{\mu 2} \cdot x^2 \log(x)$	
gallbladder	$\beta_{\mu 1} \cdot x^{-1}$	$\beta_{\sigma 1} \cdot x^2$
gluteus maximus left	$\beta_{\mu 1} \cdot x^3$	
gluteus maximus right	$\beta_{\mu 1} \cdot x^{-2} + \beta_{\mu 2} \cdot x^3$	
gluteus medius left	$\beta_{\mu 1} \cdot x^{-1} + \beta_{\mu 2} \cdot x^3$	
gluteus medius right	$\beta_{\mu 1} \cdot x^{0.5} + \beta_{\mu 2} \cdot x^2$	$\beta_{\sigma 1} \cdot x^2$
gluteus minimus left	$\beta_{\mu 1} \cdot x^{0.5} + \beta_{\mu 2} \cdot x^1$	$\beta_{\sigma 1} \cdot x^{0.5} + \beta_{\sigma 2} \cdot x^1$
gluteus minimus right	$\beta_{\mu 1} \cdot log(x) + \beta_{\mu 2} \cdot x^{0.5}$	$\beta_{\sigma 1} \cdot x^2 + \beta_{\sigma 2} \cdot x^2 \log(x)$
heart	$\beta_{\mu 1} \cdot x^{-1} + \beta_{\mu 2} \cdot x^3$	$\beta_{\sigma 1} \cdot x^3$
hip left	$\beta_{\mu 1} \cdot x^2 + \beta_{\mu 2} \cdot x^2 \log(x)$	$\beta_{\sigma 1} \cdot log(x)$
hip right	$\beta_{\mu 1} \cdot x^2 + \beta_{\mu 2} \cdot x^2 \log(x)$	$\beta_{\sigma 1} \cdot log(x)$
iliac artery left	$\beta_{\mu 1} \cdot x^2 + \beta_{\mu 2} \cdot x^3$	$\beta_{\sigma 1} \cdot x^{0.5}$
iliac artery right	$\beta_{\mu 1} \cdot x^2 + \beta_{\mu 2} \cdot x^2 \log(x)$	$\beta_{\sigma 1} \cdot x^3 + \beta_{\sigma 2} \cdot x^3 \log(x)$
iliac vena left	$\beta_{\mu 1} \cdot x^{-0.5} + \beta_{\mu 2} \cdot x^2$	$\beta_{\sigma 1} \cdot x^3$
iliac vena right	$\beta_{\mu 1} \cdot log(x) + \beta_{\mu 2} \cdot x^1$	$\beta_{\sigma 1} \cdot x^{-0.5} + \beta_{\sigma 2} \cdot x^2$
iliopsoas left	$\beta_{\mu 1} \cdot x^{-2} + \beta_{\mu 2} \cdot x^{-2} \log(x) + \beta_{\mu 3} \cdot x^{-0.5}$	$\beta_{\sigma 1} \cdot x^3$
iliopsoas right	$\beta_{\mu 1} \cdot x^{-2} + \beta_{\mu 2} \cdot x^{-2} \log(x) + \beta_{\mu 3} \cdot x^{-1}$	$\beta_{\sigma 1} \cdot x^3$
inferior vena cava	$\beta_{\mu 1} \cdot x^{-0.5} + \beta_{\mu 2} \cdot x^1$	$\beta_{\sigma 1} \cdot x^{0.5} + \beta_{\sigma 2} \cdot x^3$
kidney left	$\beta_{\mu 1} \cdot x^{-0.5} + \beta_{\mu 2} \cdot x^3$	
kidney right	$\beta_{\mu 1} \cdot x^{-0.5} + \beta_{\mu 2} \cdot x^3$	
liver	$\beta_{\mu 1} \cdot x^{0.5} + \beta_{\mu 2} \cdot x^2$	$\beta_{\sigma 1} \cdot x^3$
lung lower lobe left	$\beta_{\mu 1} \cdot x^3 + \beta_{\mu 2} \cdot x^3 \log(x)$	$\beta_{\sigma 1} \cdot x^3 + \beta_{\sigma 2} \cdot x^3 \log(x)$
lung lower lobe right	$\beta_{\mu 1} \cdot x^3 + \beta_{\mu 2} \cdot x^3 \log(x)$	$\beta_{\sigma 1} \cdot x^3 + \beta_{\sigma 2} \cdot x^3 \log(x)$
lung middle lobe right	$\beta_{\mu 1} \cdot x^2 + \beta_{\mu 2} \cdot x^3$	
lung upper lobe left	$\beta_{\mu 1} \cdot x^2 + \beta_{\mu 2} \cdot x^3$	$\beta_{\sigma 1} \cdot x^3 + \beta_{\sigma 2} \cdot x^3 \log(x)$
lung upper lobe right	$\beta_{\mu 1} \cdot x^2 + \beta_{\mu 2} \cdot x^3$	$\beta_{\sigma 1} \cdot x^1 + \beta_{\sigma 2} \cdot x^3$
pancreas	$\beta_{\mu 1} \cdot x^1 + \beta_{\mu 2} \cdot x^1 \log(x)$	$\beta_{\sigma 1} \cdot x^{-1} + \beta_{\sigma 2} \cdot x^{-0.5}$
portal vein and splenic vein	$\beta_{\mu 1} \cdot x^{0.5} + \beta_{\mu 2} \cdot x^3$	$\beta_{\sigma 1} \cdot x^2 + \beta_{\sigma 2} \cdot x^2 \log(x)$
pulmonary vein	$\beta_{\mu 1} \cdot x^{-2} + \beta_{\mu 2} \cdot x^1$	$\beta_{\sigma 1} \cdot x^{-1} + \beta_{\sigma 2} \cdot x^{-1} \log(x)$
rib left 1	$\beta_{\mu 1} \cdot log(x) + \beta_{\mu 2} \cdot x^2$	$\beta_{\sigma 1} \cdot x^2 + \beta_{\sigma 2} \cdot x^2 \log(x)$
rib left 10	$\beta_{\mu 1} \cdot x^1 + \beta_{\mu 2} \cdot x^3$	$\beta_{\sigma 1} \cdot x^2 + \beta_{\sigma 2} \cdot x^3$
rib left 11	$\beta_{\mu 1} \cdot x^2 + \beta_{\mu 2} \cdot x^3$	$\beta_{\sigma 1} \cdot x^2 + \beta_{\sigma 2} \cdot x^2 \log(x)$
rib left 12	$\beta_{\mu 1} \cdot x^2 + \beta_{\mu 2} \cdot x^3$	$\beta_{\sigma 1} \cdot log(x) + \beta_{\sigma 2} \cdot x^3$

Structure	FP_μ(age)	FP_σ(age)
rib left 2	$\beta_{\mu1} \cdot x^3 + \beta_{\mu2} \cdot x^3 \log(x)$	$\beta_{\sigma1} \cdot x^{0.5} + \beta_{\sigma2} \cdot x^3$
rib left 3	$\beta_{\mu1} \cdot x^{0.5}$	$\beta_{\sigma1} \cdot x^{-2}$
rib left 4	$\beta_{\mu1} \cdot x^{-2}$	$\beta_{\sigma1} \cdot x^{-1}$
rib left 5	$\beta_{\mu1} \cdot x^{-2}$	$\beta_{\sigma1} \cdot x^{-0.5}$
rib left 6	$\beta_{\mu1} \cdot x^{-2}$	$\beta_{\sigma1} \cdot log(x)$
rib left 7	$\beta_{\mu1} \cdot x^{-0.5}$	$\beta_{\sigma1} \cdot log(x)$
rib left 8	$\beta_{\mu1} \cdot x^{-0.5}$	$\beta_{\sigma1} \cdot log(x)$
rib left 9	$\beta_{\mu1} \cdot x^{-1}$	$\beta_{\sigma1} \cdot x^{-0.5}$
rib right 1	$\beta_{\mu1} \cdot x^{0.5} + \beta_{\mu2} \cdot x^1$	$\beta_{\sigma1} \cdot x^1 + \beta_{\sigma2} \cdot x^2$
rib right 10	$\beta_{\mu1} \cdot x^{0.5} + \beta_{\mu2} \cdot x^3$	$\beta_{\sigma1} \cdot x^2 + \beta_{\sigma2} \cdot x^3$
rib right 11	$\beta_{\mu1} \cdot x^1 + \beta_{\mu2} \cdot x^3$	$\beta_{\sigma1} \cdot x^2 + \beta_{\sigma2} \cdot x^3$
rib right 12	$\beta_{\mu1} \cdot x^{-2}$	
rib right 2	$\beta_{\mu1} \cdot x^{-2} + \beta_{\mu2} \cdot x^{0.5}$	
rib right 3	$\beta_{\mu1} \cdot x^1$	$\beta_{\sigma1} \cdot x^{-2}$
rib right 4	$\beta_{\mu1} \cdot x^{-2}$	$\beta_{\sigma1} \cdot x^{-1}$
rib right 5	$\beta_{\mu1} \cdot x^{-2}$	$\beta_{\sigma1} \cdot x^{-0.5}$
rib right 6	$\beta_{\mu1} \cdot x^{-2}$	$\beta_{\sigma1} \cdot log(x)$
rib right 7	$\beta_{\mu1} \cdot x^{-1}$	$\beta_{\sigma1} \cdot log(x)$
rib right 8	$\beta_{\mu1} \cdot x^{-1}$	$\beta_{\sigma1} \cdot x^{-0.5}$
rib right 9	$\beta_{\mu1} \cdot x^{-1}$	$\beta_{\sigma1} \cdot x^{-0.5}$
sacrum	$\beta_{\mu1} \cdot x^2 + \beta_{\mu2} \cdot x^3$	$\beta_{\sigma1} \cdot x^{0.5}$
scapula left	$\beta_{\mu1} \cdot x^{0.5} + \beta_{\mu2} \cdot x^2$	$\beta_{\sigma1} \cdot x^2 + \beta_{\sigma2} \cdot x^3$
scapula right	$\beta_{\mu1} \cdot x^1 + \beta_{\mu2} \cdot x^2$	$\beta_{\sigma1} \cdot x^3 + \beta_{\sigma2} \cdot x^3 \log(x)$
small bowel	$\beta_{\mu1} \cdot log(x) + \beta_{\mu2} \cdot x^3$	$\beta_{\sigma1} \cdot x^{-2}$
spleen	$\beta_{\mu1} \cdot x^{-2} + \beta_{\mu2} \cdot x^3$	$\beta_{\sigma1} \cdot x^2 + \beta_{\sigma2} \cdot x^3$
sternum	$\beta_{\mu1} \cdot x^{0.5} + \beta_{\mu2} \cdot x^3$	$\beta_{\sigma1} \cdot log(x)$
stomach	$\beta_{\mu1} \cdot x^1 + \beta_{\mu2} \cdot x^3$	$\beta_{\sigma1} \cdot x^2$
subclavian artery left	$\beta_{\mu1} \cdot x^2 + \beta_{\mu2} \cdot x^3$	$\beta_{\sigma1} \cdot x^{-2} + \beta_{\sigma2} \cdot x^3$
subclavian artery right	$\beta_{\mu1} \cdot x^{-2} + \beta_{\mu2} \cdot x^3 + \beta_{\mu3} \cdot x^3 \log(x)$	$\beta_{\sigma1} \cdot x^{-1}$
superior vena cava	$\beta_{\mu1} \cdot x^2 + \beta_{\mu2} \cdot x^3$	$\beta_{\sigma1} \cdot x^2 + \beta_{\sigma2} \cdot x^3$
thyroid gland	$\beta_{\mu1} \cdot x^{-0.5} + \beta_{\mu2} \cdot x^2$	$\beta_{\sigma1} \cdot x^3 + \beta_{\sigma2} \cdot x^3 \log(x)$
trachea	$\beta_{\mu1} \cdot log(x) + \beta_{\mu2} \cdot x^3$	$\beta_{\sigma1} \cdot x^{-2} + \beta_{\sigma2} \cdot x^2$
urinary bladder	$\beta_{\mu1} \cdot x^2 + \beta_{\mu2} \cdot x^3 + \beta_{\mu3} \cdot x^3 \log(x)$	$\beta_{\sigma1} \cdot x^3$
vertebrae C1	$\beta_{\mu1} \cdot x^2 + \beta_{\mu2} \cdot x^3$	$\beta_{\sigma1} \cdot x^3$
vertebrae C2	$\beta_{\mu1} \cdot x^2 + \beta_{\mu2} \cdot x^2 \log(x)$	
vertebrae C3	$\beta_{\mu1} \cdot x^2 + \beta_{\mu2} \cdot x^3$	
vertebrae C4	$\beta_{\mu1} \cdot x^2 + \beta_{\mu2} \cdot x^3$	
vertebrae C5	$\beta_{\mu1} \cdot x^2 + \beta_{\mu2} \cdot x^3$	$\beta_{\sigma1} \cdot x^2$
vertebrae C6	$\beta_{\mu1} \cdot x^2 + \beta_{\mu2} \cdot x^2 \log(x)$	$\beta_{\sigma1} \cdot x^{-1}$
vertebrae C7	$\beta_{\mu1} \cdot x^2 + \beta_{\mu2} \cdot x^3$	$\beta_{\sigma1} \cdot x^3 + \beta_{\sigma2} \cdot x^3 \log(x)$
vertebrae L1	$\beta_{\mu1} \cdot x^{0.5}$	$\beta_{\sigma1} \cdot x^{-1}$
vertebrae L2	$\beta_{\mu1} \cdot x^{0.5}$	$\beta_{\sigma1} \cdot x^{-2}$
vertebrae L3	$\beta_{\mu1} \cdot x^{0.5}$	$\beta_{\sigma1} \cdot x^{-2}$
vertebrae L4	$\beta_{\mu1} \cdot x^2 + \beta_{\mu2} \cdot x^2 \log(x)$	$\beta_{\sigma1} \cdot x^{-2}$
vertebrae L5	$\beta_{\mu1} \cdot x^2 + \beta_{\mu2} \cdot x^3$	$\beta_{\sigma1} \cdot x^{-2}$
vertebrae S1	$\beta_{\mu1} \cdot x^3 + \beta_{\mu2} \cdot x^3 \log(x)$	$\beta_{\sigma1} \cdot x^{-0.5}$
vertebrae T1	$\beta_{\mu1} \cdot log(x)$	$\beta_{\sigma1} \cdot x^{-2}$
vertebrae T10	$\beta_{\mu1} \cdot x^3 + \beta_{\mu2} \cdot x^3 \log(x)$	$\beta_{\sigma1} \cdot x^{-2}$
vertebrae T11	$\beta_{\mu1} \cdot x^2 + \beta_{\mu2} \cdot x^3$	$\beta_{\sigma1} \cdot x^2 + \beta_{\sigma2} \cdot x^2 \log(x)$
vertebrae T12	$\beta_{\mu1} \cdot x^{0.5}$	$\beta_{\sigma1} \cdot x^{-0.5}$
vertebrae T2	$\beta_{\mu1} \cdot log(x)$	$\beta_{\sigma1} \cdot x^{-2}$
vertebrae T3	$\beta_{\mu1} \cdot x^1 + \beta_{\mu2} \cdot x^3$	$\beta_{\sigma1} \cdot x^{-2}$

Structure	$\text{FP}_{\mu}(\text{age})$	$\text{FP}_{\sigma}(\text{age})$
vertebrae T4	$\beta_{\mu 1} \cdot x^2 + \beta_{\mu 2} \cdot x^3$	$\beta_{\sigma 1} \cdot x^{-1}$
vertebrae T5	$\beta_{\mu 1} \cdot x^2 + \beta_{\mu 2} \cdot x^3$	$\beta_{\sigma 1} \cdot x^{-2}$
vertebrae T6	$\beta_{\mu 1} \cdot x^2 + \beta_{\mu 2} \cdot x^3$	$\beta_{\sigma 1} \cdot x^{-0.5}$
vertebrae T7	$\beta_{\mu 1} \cdot x^3 + \beta_{\mu 2} \cdot x^3 \log(x)$	$\beta_{\sigma 1} \cdot x^{-1}$
vertebrae T8	$\beta_{\mu 1} \cdot x^3 + \beta_{\mu 2} \cdot x^3 \log(x)$	$\beta_{\sigma 1} \cdot x^{-2}$
vertebrae T9	$\beta_{\mu 1} \cdot x^3 + \beta_{\mu 2} \cdot x^3 \log(x)$	$\beta_{\sigma 1} \cdot x^{-2}$

Supplementary Table 6: Fractional polynomial (FP) models for non-linear age effects in the μ (location) and σ (scale) components of the GG distribution with age x. For each structure, FP functions, selected from the set $\{-2, -1, -0.5, 0, 0.5, 1, 2, 3\}$ and fitted using one to three terms, were chosen based on the Bayesian information criterion (BIC). These FP functions enable flexible modeling of the age effect while balancing model fit and complexity.

Fractional polynomials for age in attenuation GAMLSS

Structure	Cont	FP_μ(age)	FP_σ(age)
adrenal gland l	×	$\beta_{\mu1} \cdot x^{0.5} + \beta_{\mu2} \cdot x^{0.5}\ \log(x)$	$\beta_{\sigma1} \cdot x^{-0.5} + \beta_{\sigma2} \cdot log(x)$
adrenal gland l	✓	$\beta_{\mu1} \cdot x^{-1} + \beta_{\mu2} \cdot x^{3}$	$\beta_{\sigma1} \cdot x^{3} + \beta_{\sigma2} \cdot x^{3}\ \log(x)$
adrenal gland r	×	$\beta_{\mu1} \cdot x^{-2} + \beta_{\mu2} \cdot x^{-1} + \beta_{\mu3} \cdot x^{-0.5}$	$\beta_{\sigma1} \cdot x^{2} + \beta_{\sigma2} \cdot x^{2}\ \log(x) + \beta_{\sigma3} \cdot x^{3}$
adrenal gland r	✓	$\beta_{\mu1} \cdot x^{-1} + \beta_{\mu2} \cdot x^{3}$	$\beta_{\sigma1} \cdot x^{3} + \beta_{\sigma2} \cdot x^{3}\ \log(x)$
aorta	×	$\beta_{\mu1} \cdot x^{2} + \beta_{\mu2} \cdot x^{3}$	$\beta_{\sigma1} \cdot x^{3}$
aorta	✓	$\beta_{\mu1} \cdot x^{1} + \beta_{\mu2} \cdot x^{1}\ \log(x)$	$\beta_{\sigma1} \cdot x^{3} + \beta_{\sigma2} \cdot x^{3}\ \log(x)$
atrial appendage l	×	$\beta_{\mu1} \cdot x^{0.5} + \beta_{\mu2} \cdot x^{3}$	$\beta_{\sigma1} \cdot x^{3} + \beta_{\sigma2} \cdot x^{3}\ \log(x)$
atrial appendage l	✓	$\beta_{\mu1} \cdot x^{2} + \beta_{\mu2} \cdot x^{2}\ \log(x)$	$\beta_{\sigma1} \cdot x^{2} + \beta_{\sigma2} \cdot x^{3}$
brachiocephalic trunk	×	$\beta_{\mu1} \cdot x^{1} + \beta_{\mu2} \cdot x^{2}$	$\beta_{\sigma1} \cdot x^{0.5} + \beta_{\sigma2} \cdot x^{0.5}\ \log(x)$
brachiocephalic trunk	✓	$\beta_{\mu1} \cdot x^{3} + \beta_{\mu2} \cdot x^{3}\ \log(x) + \beta_{\mu3} \cdot x^{3}\ \log(x)^2$	$\beta_{\sigma1} \cdot x^{3} + \beta_{\sigma2} \cdot x^{3}\ \log(x)$
brachiocephalic vein l	×	$\beta_{\mu1} \cdot x^{1} + \beta_{\mu2} \cdot x^{3}$	
brachiocephalic vein l	✓	$\beta_{\mu1} \cdot x^{2}$	
brachiocephalic vein r	×	$\beta_{\mu1} \cdot x^{-0.5}$	
brachiocephalic vein r	✓	$\beta_{\mu1} \cdot x^{3} + \beta_{\mu2} \cdot x^{3}\ \log(x)$	$\beta_{\sigma1} \cdot x^{2} + \beta_{\sigma2} \cdot x^{3}$
brain	×	$\beta_{\mu1} \cdot x^{2} + \beta_{\mu2} \cdot x^{3}$	$\beta_{\sigma1} \cdot x^{2} + \beta_{\sigma2} \cdot x^{3}$
brain	✓	$\beta_{\mu1} \cdot x^{1} + \beta_{\mu2} \cdot x^{1}\ \log(x)$	
clavicula l	×	$\beta_{\mu1} \cdot log(x) + \beta_{\mu2} \cdot log(x)^2$	$\beta_{\sigma1} \cdot x^{0.5}$
clavicula l	✓	$\beta_{\mu1} \cdot x^{-2} + \beta_{\mu2} \cdot x^{-2}\ \log(x) + \beta_{\mu3} \cdot x^{-2}\ \log(x)^2$	$\beta_{\sigma1} \cdot x^{3}$
clavicula r	×	$\beta_{\mu1} \cdot x^{-0.5} + \beta_{\mu2} \cdot log(x)$	$\beta_{\sigma1} \cdot x^{-0.5}$
clavicula r	✓	$\beta_{\mu1} \cdot x^{-2} + \beta_{\mu2} \cdot x^{-2}\ \log(x) + \beta_{\mu3} \cdot x^{-2}\ \log(x)^2$	$\beta_{\sigma1} \cdot x^{3}$
colon	×	$\beta_{\mu1} \cdot x^{-2}$	$\beta_{\sigma1} \cdot x^{2}$
colon	✓	$\beta_{\mu1} \cdot x^{-2} + \beta_{\mu2} \cdot x^{3}$	
common carotid artery l	×	$\beta_{\mu1} \cdot x^{2} + \beta_{\mu2} \cdot x^{3}$	
common carotid artery l	✓	$\beta_{\mu1} \cdot x^{-2} + \beta_{\mu2} \cdot x^{3}$	$\beta_{\sigma1} \cdot x^{3} + \beta_{\sigma2} \cdot x^{3}\ \log(x)$
common carotid artery r	×	$\beta_{\mu1} \cdot x^{2} + \beta_{\mu2} \cdot x^{3}$	$\beta_{\sigma1} \cdot x^{2}$
common carotid artery r	✓	$\beta_{\mu1} \cdot x^{-2} + \beta_{\mu2} \cdot x^{3}$	$\beta_{\sigma1} \cdot x^{3} + \beta_{\sigma2} \cdot x^{3}\ \log(x)$
costal cartilages	×	$\beta_{\mu1} \cdot x^{1} + \beta_{\mu2} \cdot x^{2} + \beta_{\mu3} \cdot x^{3}$	$\beta_{\sigma1} \cdot x^{3}$
costal cartilages	✓	$\beta_{\mu1} \cdot x^{1} + \beta_{\mu2} \cdot x^{2} + \beta_{\mu3} \cdot x^{2}\ \log(x)$	$\beta_{\sigma1} \cdot x^{3}$
duodenum	×	$\beta_{\mu1} \cdot x^{-1}$	$\beta_{\sigma1} \cdot x^{3}$
duodenum	✓	$\beta_{\mu1} \cdot x^{2} + \beta_{\mu2} \cdot x^{3}$	$\beta_{\sigma1} \cdot x^{-2} + \beta_{\sigma2} \cdot x^{3}$
esophagus	×	$\beta_{\mu1} \cdot log(x)$	$\beta_{\sigma1} \cdot x^{3}$
esophagus	✓	$\beta_{\mu1} \cdot x^{3}$	$\beta_{\sigma1} \cdot x^{3}$
gallbladder	×	$\beta_{\mu1} \cdot x^{-0.5}$	$\beta_{\sigma1} \cdot x^{3}$
gallbladder	✓	$\beta_{\mu1} \cdot x^{2} + \beta_{\mu2} \cdot x^{2}\ \log(x)$	$\beta_{\sigma1} \cdot x^{3}$
gluteus maximus l	×	$\beta_{\mu1} \cdot x^{-0.5}$	$\beta_{\sigma1} \cdot x^{1}$
gluteus maximus l	✓	$\beta_{\mu1} \cdot log(x)$	$\beta_{\sigma1} \cdot x^{-1}$
gluteus maximus r	×	$\beta_{\mu1} \cdot x^{-0.5}$	$\beta_{\sigma1} \cdot x^{2}$
gluteus maximus r	✓	$\beta_{\mu1} \cdot log(x)$	$\beta_{\sigma1} \cdot x^{-0.5}$
gluteus medius l	×	$\beta_{\mu1} \cdot x^{-0.5} + \beta_{\mu2} \cdot x^{3}$	$\beta_{\sigma1} \cdot x^{3} + \beta_{\sigma2} \cdot x^{3}\ \log(x)$
gluteus medius l	✓	$\beta_{\mu1} \cdot log(x) + \beta_{\mu2} \cdot x^{3}$	$\beta_{\sigma1} \cdot x^{1}$
gluteus medius r	×	$\beta_{\mu1} \cdot x^{-1} + \beta_{\mu2} \cdot x^{3}$	$\beta_{\sigma1} \cdot x^{3} + \beta_{\sigma2} \cdot x^{3}\ \log(x)$
gluteus medius r	✓	$\beta_{\mu1} \cdot x^{-0.5} + \beta_{\mu2} \cdot x^{3}$	$\beta_{\sigma1} \cdot x^{1}$
gluteus minimus l	×	$\beta_{\mu1} \cdot x^{1} + \beta_{\mu2} \cdot x^{2} + \beta_{\mu3} \cdot x^{2}\ \log(x)$	$\beta_{\sigma1} \cdot x^{1} + \beta_{\sigma2} \cdot x^{2} + \beta_{\sigma3} \cdot x^{2}\ \log(x)$
gluteus minimus l	✓	$\beta_{\mu1} \cdot x^{1} + \beta_{\mu2} \cdot x^{3} + \beta_{\mu3} \cdot x^{3}\ \log(x)$	$\beta_{\sigma1} \cdot x^{3} + \beta_{\sigma2} \cdot x^{3}\ \log(x) + \beta_{\sigma3} \cdot x^{3}\ \log(x)^2$
gluteus minimus r	×	$\beta_{\mu1} \cdot x^{1} + \beta_{\mu2} \cdot x^{2} + \beta_{\mu3} \cdot x^{2}\ \log(x)$	$\beta_{\sigma1} \cdot x^{-0.5} + \beta_{\sigma2} \cdot x^{3} + \beta_{\sigma3} \cdot x^{3}\ \log(x)$
gluteus minimus r	✓	$\beta_{\mu1} \cdot x^{-2} + \beta_{\mu2} \cdot x^{3}$	$\beta_{\sigma1} \cdot x^{3} + \beta_{\sigma2} \cdot x^{3}\ \log(x)$
heart	×	$\beta_{\mu1} \cdot x^{2} + \beta_{\mu2} \cdot x^{3}$	$\beta_{\sigma1} \cdot x^{-2} + \beta_{\sigma2} \cdot x^{-1}$
heart	✓	$\beta_{\mu1} \cdot x^{2} + \beta_{\mu2} \cdot x^{2}\ \log(x)$	$\beta_{\sigma1} \cdot x^{3} + \beta_{\sigma2} \cdot x^{3}\ \log(x)$
hip l	×	$\beta_{\mu1} \cdot x^{1}$	
hip l	✓	$\beta_{\mu1} \cdot x^{-2} + \beta_{\mu2} \cdot x^{-2}\ \log(x) + \beta_{\mu3} \cdot x^{-1}$	$\beta_{\sigma1} \cdot x^{2}$
hip r	×	$\beta_{\mu1} \cdot x^{1}$	
hip r	✓	$\beta_{\mu1} \cdot x^{-2} + \beta_{\mu2} \cdot x^{-2}\ \log(x) + \beta_{\mu3} \cdot x^{-1}$	$\beta_{\sigma1} \cdot x^{2}$
iliac artery l	×	$\beta_{\mu1} \cdot x^{-2} + \beta_{\mu2} \cdot x^{3} + \beta_{\mu3} \cdot x^{3}\ \log(x)$	$\beta_{\sigma1} \cdot x^{2} + \beta_{\sigma2} \cdot x^{3} + \beta_{\sigma3} \cdot x^{3}\ \log(x)$

Structure	Cont	FP$_\mu$(age)	FP$_\sigma$(age)
iliac artery l	✓	$\beta_{\mu1} \cdot x^1 + \beta_{\mu2} \cdot x^2$	$\beta_{\sigma1} \cdot x^3$
iliac artery r	×	$\beta_{\mu1} \cdot x^2 + \beta_{\mu2} \cdot x^2\ \log(x) + \beta_{\mu3} \cdot x^2\ \log(x)^2$	$\beta_{\sigma1} \cdot x^2 + \beta_{\sigma2} \cdot x^2\ \log(x) + \beta_{\sigma3} \cdot x^3$
iliac artery r	✓	$\beta_{\mu1} \cdot x^1 + \beta_{\mu2} \cdot x^2$	$\beta_{\sigma1} \cdot x^3$
iliac vena l	×	$\beta_{\mu1} \cdot x^3 + \beta_{\mu2} \cdot x^3\ \log(x)$	$\beta_{\sigma1} \cdot x^3 + \beta_{\sigma2} \cdot x^3\ \log(x)$
iliac vena l	✓	$\beta_{\mu1} \cdot x^1$	
iliac vena r	×	$\beta_{\mu1} \cdot x^3 + \beta_{\mu2} \cdot x^3\ \log(x)$	$\beta_{\sigma1} \cdot x^3 + \beta_{\sigma2} \cdot x^3\ \log(x)$
iliac vena r	✓	$\beta_{\mu1} \cdot x^1$	$\beta_{\sigma1} \cdot x^3$
iliopsoas l	×	$\beta_{\mu1} \cdot x^3 + \beta_{\mu2} \cdot x^3\ \log(x)$	$\beta_{\sigma1} \cdot x^3 + \beta_{\sigma2} \cdot x^3\ \log(x)$
iliopsoas l	✓	$\beta_{\mu1} \cdot x^2 + \beta_{\mu2} \cdot x^3$	$\beta_{\sigma1} \cdot x^1$
iliopsoas r	×	$\beta_{\mu1} \cdot x^3 + \beta_{\mu2} \cdot x^3\ \log(x)$	$\beta_{\sigma1} \cdot x^3 + \beta_{\sigma2} \cdot x^3\ \log(x)$
iliopsoas r	✓	$\beta_{\mu1} \cdot x^2 + \beta_{\mu2} \cdot x^2\ \log(x)$	$\beta_{\sigma1} \cdot x^1$
inferior vena cava	×	$\beta_{\mu1} \cdot x^{0.5}$	$\beta_{\sigma1} \cdot x^{-1}$
inferior vena cava	✓	$\beta_{\mu1} \cdot x^{0.5}$	$\beta_{\sigma1} \cdot x^3$
kidney l	×	$\beta_{\mu1} \cdot x^{-2} + \beta_{\mu2} \cdot x^{-2}\ \log(x)$	$\beta_{\sigma1} \cdot x^2 + \beta_{\sigma2} \cdot x^3$
kidney l	✓	$\beta_{\mu1} \cdot log(x)$	$\beta_{\sigma1} \cdot x^3$
kidney r	×	$\beta_{\mu1} \cdot x^{-2} + \beta_{\mu2} \cdot x^{-2}\ \log(x)$	$\beta_{\sigma1} \cdot x^3 + \beta_{\sigma2} \cdot x^3\ \log(x)$
kidney r	✓	$\beta_{\mu1} \cdot log(x)$	$\beta_{\sigma1} \cdot x^3$
liver	×	$\beta_{\mu1} \cdot x^{-2} + \beta_{\mu2} \cdot x^{-2}\ \log(x)$	$\beta_{\sigma1} \cdot x^2 + \beta_{\sigma2} \cdot x^3$
liver	✓	$\beta_{\mu1} \cdot x^{-1}$	$\beta_{\sigma1} \cdot x^3$
lung lower lobe l	×	$\beta_{\mu1} \cdot x^{-2}$	$\beta_{\sigma1} \cdot x^3$
lung lower lobe l	✓	$\beta_{\mu1} \cdot x^3 + \beta_{\mu2} \cdot x^3\ \log(x)$	$\beta_{\sigma1} \cdot x^3 + \beta_{\sigma2} \cdot x^3\ \log(x)$
lung lower lobe r	×	$\beta_{\mu1} \cdot x^{-2}$	$\beta_{\sigma1} \cdot x^3$
lung lower lobe r	✓	$\beta_{\mu1} \cdot x^3 + \beta_{\mu2} \cdot x^3\ \log(x)$	$\beta_{\sigma1} \cdot x^3 + \beta_{\sigma2} \cdot x^3\ \log(x)$
lung middle lobe r	×	$\beta_{\mu1} \cdot x^3$	
lung middle lobe r	✓	$\beta_{\mu1} \cdot x^3 + \beta_{\mu2} \cdot x^3\ \log(x)$	$\beta_{\sigma1} \cdot x^3 + \beta_{\sigma2} \cdot x^3\ \log(x)$
lung upper lobe l	×	$\beta_{\mu1} \cdot x^{-2}$	
lung upper lobe l	✓	$\beta_{\mu1} \cdot x^3 + \beta_{\mu2} \cdot x^3\ \log(x)$	$\beta_{\sigma1} \cdot x^3 + \beta_{\sigma2} \cdot x^3\ \log(x)$
lung upper lobe r	×	$\beta_{\mu1} \cdot x^{-2}$	
lung upper lobe r	✓	$\beta_{\mu1} \cdot x^3 + \beta_{\mu2} \cdot x^3\ \log(x)$	$\beta_{\sigma1} \cdot x^3 + \beta_{\sigma2} \cdot x^3\ \log(x)$
pancreas	×	$\beta_{\mu1} \cdot x^{-0.5} + \beta_{\mu2} \cdot log(x)$	$\beta_{\sigma1} \cdot x^2 + \beta_{\sigma2} \cdot x^3$
pancreas	✓	$\beta_{\mu1} \cdot x^2 + \beta_{\mu2} \cdot x^2\ \log(x)$	$\beta_{\sigma1} \cdot x^1$
portal vein and splenic vein	×	$\beta_{\mu1} \cdot x^3 + \beta_{\mu2} \cdot x^3\ \log(x)$	$\beta_{\sigma1} \cdot x^{-1} + \beta_{\sigma2} \cdot x^{-0.5}$
portal vein and splenic vein	✓	$\beta_{\mu1} \cdot x^{-1}$	$\beta_{\sigma1} \cdot x^3$
prostate	×	$\beta_{\mu1} \cdot x^{-2} + \beta_{\mu2} \cdot x^{-1}$	$\beta_{\sigma1} \cdot x^1$
prostate	✓	$\beta_{\mu1} \cdot x^{-2} + \beta_{\mu2} \cdot x^{-2}\ \log(x)$	$\beta_{\sigma1} \cdot x^2 + \beta_{\sigma2} \cdot x^3$
pulmonary vein	×	$\beta_{\mu1} \cdot x^3$	$\beta_{\sigma1} \cdot x^{-1}$
pulmonary vein	✓	$\beta_{\mu1} \cdot x^2 + \beta_{\mu2} \cdot x^2\ \log(x)$	$\beta_{\sigma1} \cdot x^3 + \beta_{\sigma2} \cdot x^3\ \log(x)$
rib l 1	×	$\beta_{\mu1} \cdot x^{-2} + \beta_{\mu2} \cdot x^{-2}\ \log(x) + \beta_{\mu3} \cdot x^{-1}$	$\beta_{\sigma1} \cdot x^{0.5} + \beta_{\sigma2} \cdot x^{0.5}\ \log(x) + \beta_{\sigma3} \cdot x^1$
rib l 1	✓	$\beta_{\mu1} \cdot log(x) + \beta_{\mu2} \cdot log(x)^2$	$\beta_{\sigma1} \cdot x^1 + \beta_{\sigma2} \cdot x^2$
rib l 10	×	$\beta_{\mu1} \cdot x^2 + \beta_{\mu2} \cdot x^3$	
rib l 10	✓	$\beta_{\mu1} \cdot x^{-2} + \beta_{\mu2} \cdot x^{-2}\ \log(x) + \beta_{\mu3} \cdot x^{-2}\ \log(x)^2$	$\beta_{\sigma1} \cdot x^{0.5} + \beta_{\sigma2} \cdot x^{0.5}\ \log(x) + \beta_{\sigma3} \cdot x^{0.5}\ \log(x)^2$
rib l 11	×	$\beta_{\mu1} \cdot x^3 + \beta_{\mu2} \cdot x^3\ \log(x)$	
rib l 11	✓	$\beta_{\mu1} \cdot x^{-2} + \beta_{\mu2} \cdot x^{-2}\ \log(x) + \beta_{\mu3} \cdot x^{-2}\ \log(x)^2$	$\beta_{\sigma1} \cdot x^{0.5} + \beta_{\sigma2} \cdot x^{0.5}\ \log(x) + \beta_{\sigma3} \cdot x^1$
rib l 12	×	$\beta_{\mu1} \cdot x^2 + \beta_{\mu2} \cdot x^3$	$\beta_{\sigma1} \cdot x^2$
rib l 12	✓	$\beta_{\mu1} \cdot x^{-2} + \beta_{\mu2} \cdot x^{-2}\ \log(x) + \beta_{\mu3} \cdot x^{-2}\ \log(x)^2$	$\beta_{\sigma1} \cdot x^1 + \beta_{\sigma2} \cdot x^1\ \log(x) + \beta_{\sigma3} \cdot x^1\ \log(x)^2$
rib l 2	×	$\beta_{\mu1} \cdot x^2 + \beta_{\mu2} \cdot x^2\ \log(x)$	
rib l 2	✓	$\beta_{\mu1} \cdot x^3 + \beta_{\mu2} \cdot x^3\ \log(x) + \beta_{\mu3} \cdot x^3\ \log(x)^2$	$\beta_{\sigma1} \cdot x^3 + \beta_{\sigma2} \cdot x^3\ \log(x)$
rib l 3	×	$\beta_{\mu1} \cdot x^2 + \beta_{\mu2} \cdot x^2\ \log(x)$	$\beta_{\sigma1} \cdot x^1$
rib l 3	✓	$\beta_{\mu1} \cdot x^3 + \beta_{\mu2} \cdot x^3\ \log(x) + \beta_{\mu3} \cdot x^3\ \log(x)^2$	$\beta_{\sigma1} \cdot x^1 + \beta_{\sigma2} \cdot x^1\ \log(x) + \beta_{\sigma3} \cdot x^1\ \log(x)^2$
rib l 4	×	$\beta_{\mu1} \cdot x^2 + \beta_{\mu2} \cdot x^2\ \log(x)$	
rib l 4	✓	$\beta_{\mu1} \cdot x^3 + \beta_{\mu2} \cdot x^3\ \log(x) + \beta_{\mu3} \cdot x^3\ \log(x)^2$	$\beta_{\sigma1} \cdot x^1 + \beta_{\sigma2} \cdot x^1\ \log(x) + \beta_{\sigma3} \cdot x^1\ \log(x)^2$
rib l 5	×	$\beta_{\mu1} \cdot x^2 + \beta_{\mu2} \cdot x^2\ \log(x)$	
rib l 5	✓	$\beta_{\mu1} \cdot x^3 + \beta_{\mu2} \cdot x^3\ \log(x) + \beta_{\mu3} \cdot x^3\ \log(x)^2$	$\beta_{\sigma1} \cdot x^3 + \beta_{\sigma2} \cdot x^3\ \log(x)$
rib l 6	×	$\beta_{\mu1} \cdot x^2 + \beta_{\mu2} \cdot x^2\ \log(x)$	
rib l 6	✓	$\beta_{\mu1} \cdot x^3 + \beta_{\mu2} \cdot x^3\ \log(x) + \beta_{\mu3} \cdot x^3\ \log(x)^2$	$\beta_{\sigma1} \cdot x^3 + \beta_{\sigma2} \cdot x^3\ \log(x)$

Structure	Cont	$\text{FP}_\mu(\text{age})$	$\text{FP}_\sigma(\text{age})$
rib l 7	×	$\beta_{\mu 1} \cdot x^2 + \beta_{\mu 2} \cdot x^2 \log(x)$	
rib l 7	✓	$\beta_{\mu 1} \cdot x^3 + \beta_{\mu 2} \cdot x^3 \log(x) + \beta_{\mu 3} \cdot x^3 \log(x)^2$	$\beta_{\sigma 1} \cdot x^3 + \beta_{\sigma 2} \cdot x^3 \log(x)$
rib l 8	×	$\beta_{\mu 1} \cdot x^2 + \beta_{\mu 2} \cdot x^2 \log(x)$	
rib l 8	✓	$\beta_{\mu 1} \cdot x^3 + \beta_{\mu 2} \cdot x^3 \log(x) + \beta_{\mu 3} \cdot x^3 \log(x)^2$	$\beta_{\sigma 1} \cdot x^3 + \beta_{\sigma 2} \cdot x^3 \log(x)$
rib l 9	×	$\beta_{\mu 1} \cdot x^1$	
rib l 9	✓	$\beta_{\mu 1} \cdot x^{0.5}$	$\beta_{\sigma 1} \cdot x^{0.5}$
rib r 1	×	$\beta_{\mu 1} \cdot x^2 + \beta_{\mu 2} \cdot x^2 \log(x) + \beta_{\mu 3} \cdot x^2 \log(x)^2$	$\beta_{\sigma 1} \cdot x^{0.5} + \beta_{\sigma 2} \cdot x^{0.5} \log(x) + \beta_{\sigma 3} \cdot x^{0.5} \log(x)^2$
rib r 1	✓	$\beta_{\mu 1} \cdot x^{-0.5} + \beta_{\mu 2} \cdot log(x)$	$\beta_{\sigma 1} \cdot x^{-0.5}$
rib r 10	×	$\beta_{\mu 1} \cdot x^1$	
rib r 10	✓	$\beta_{\mu 1} \cdot x^{-2} + \beta_{\mu 2} \cdot x^{-2} \log(x) + \beta_{\mu 3} \cdot x^{-2} \log(x)^2$	$\beta_{\sigma 1} \cdot x^{0.5} + \beta_{\sigma 2} \cdot x^1 + \beta_{\sigma 3} \cdot x^1 \log(x)$
rib r 11	×	$\beta_{\mu 1} \cdot x^2 + \beta_{\mu 2} \cdot x^3$	$\beta_{\sigma 1} \cdot x^3$
rib r 11	✓	$\beta_{\mu 1} \cdot x^{-2} + \beta_{\mu 2} \cdot x^{-2} \log(x) + \beta_{\mu 3} \cdot x^{-2} \log(x)^2$	$\beta_{\sigma 1} \cdot x^1 + \beta_{\sigma 2} \cdot x^1 \log(x) + \beta_{\sigma 3} \cdot x^1 \log(x)^2$
rib r 12	×	$\beta_{\mu 1} \cdot x^2 + \beta_{\mu 2} \cdot x^3$	$\beta_{\sigma 1} \cdot x^2$
rib r 12	✓	$\beta_{\mu 1} \cdot x^{-2} + \beta_{\mu 2} \cdot x^{-2} \log(x) + \beta_{\mu 3} \cdot x^{-2} \log(x)^2$	$\beta_{\sigma 1} \cdot x^1 + \beta_{\sigma 2} \cdot x^1 \log(x) + \beta_{\sigma 3} \cdot x^1 \log(x)^2$
rib r 2	×	$\beta_{\mu 1} \cdot x^1 + \beta_{\mu 2} \cdot x^3$	
rib r 2	✓	$\beta_{\mu 1} \cdot x^3 + \beta_{\mu 2} \cdot x^3 \log(x) + \beta_{\mu 3} \cdot x^3 \log(x)^2$	$\beta_{\sigma 1} \cdot x^3 + \beta_{\sigma 2} \cdot x^3 \log(x)$
rib r 3	×	$\beta_{\mu 1} \cdot x^2 + \beta_{\mu 2} \cdot x^2 \log(x)$	$\beta_{\sigma 1} \cdot x^1$
rib r 3	✓	$\beta_{\mu 1} \cdot x^3 + \beta_{\mu 2} \cdot x^3 \log(x) + \beta_{\mu 3} \cdot x^3 \log(x)^2$	$\beta_{\sigma 1} \cdot x^{0.5} + \beta_{\sigma 2} \cdot x^1 + \beta_{\sigma 3} \cdot x^1 \log(x)$
rib r 4	×	$\beta_{\mu 1} \cdot x^2 + \beta_{\mu 2} \cdot x^2 \log(x)$	$\beta_{\sigma 1} \cdot x^2$
rib r 4	✓	$\beta_{\mu 1} \cdot x^3 + \beta_{\mu 2} \cdot x^3 \log(x) + \beta_{\mu 3} \cdot x^3 \log(x)^2$	$\beta_{\sigma 1} \cdot x^3 + \beta_{\sigma 2} \cdot x^3 \log(x)$
rib r 5	×	$\beta_{\mu 1} \cdot x^2 + \beta_{\mu 2} \cdot x^2 \log(x)$	
rib r 5	✓	$\beta_{\mu 1} \cdot x^3 + \beta_{\mu 2} \cdot x^3 \log(x) + \beta_{\mu 3} \cdot x^3 \log(x)^2$	$\beta_{\sigma 1} \cdot x^3 + \beta_{\sigma 2} \cdot x^3 \log(x)$
rib r 6	×	$\beta_{\mu 1} \cdot x^2 + \beta_{\mu 2} \cdot x^2 \log(x)$	
rib r 6	✓	$\beta_{\mu 1} \cdot x^3 + \beta_{\mu 2} \cdot x^3 \log(x) + \beta_{\mu 3} \cdot x^3 \log(x)^2$	$\beta_{\sigma 1} \cdot x^3 + \beta_{\sigma 2} \cdot x^3 \log(x)$
rib r 7	×	$\beta_{\mu 1} \cdot x^2 + \beta_{\mu 2} \cdot x^2 \log(x)$	
rib r 7	✓	$\beta_{\mu 1} \cdot x^3 + \beta_{\mu 2} \cdot x^3 \log(x) + \beta_{\mu 3} \cdot x^3 \log(x)^2$	$\beta_{\sigma 1} \cdot x^3 + \beta_{\sigma 2} \cdot x^3 \log(x)$
rib r 8	×	$\beta_{\mu 1} \cdot x^2 + \beta_{\mu 2} \cdot x^2 \log(x)$	
rib r 8	✓	$\beta_{\mu 1} \cdot x^3 + \beta_{\mu 2} \cdot x^3 \log(x) + \beta_{\mu 3} \cdot x^3 \log(x)^2$	$\beta_{\sigma 1} \cdot x^3 + \beta_{\sigma 2} \cdot x^3 \log(x)$
rib r 9	×	$\beta_{\mu 1} \cdot x^1$	
rib r 9	✓	$\beta_{\mu 1} \cdot x^{-2} + \beta_{\mu 2} \cdot x^{-2} \log(x) + \beta_{\mu 3} \cdot x^{-2} \log(x)^2$	$\beta_{\sigma 1} \cdot x^{0.5}$
sacrum	×	$\beta_{\mu 1} \cdot x^1$	
sacrum	✓	$\beta_{\mu 1} \cdot x^{-2} + \beta_{\mu 2} \cdot x^{-2} \log(x) + \beta_{\mu 3} \cdot x^{-2} \log(x)^2$	$\beta_{\sigma 1} \cdot x^3$
scapula l	×	$\beta_{\mu 1} \cdot x^{-0.5} + \beta_{\mu 2} \cdot log(x)$	
scapula l	✓	$\beta_{\mu 1} \cdot log(x) + \beta_{\mu 2} \cdot log(x)^2$	
scapula r	×	$\beta_{\mu 1} \cdot x^{-0.5} + \beta_{\mu 2} \cdot x^{-0.5} \log(x)$	
scapula r	✓	$\beta_{\mu 1} \cdot x^{-0.5} + \beta_{\mu 2} \cdot log(x)$	$\beta_{\sigma 1} \cdot x^{0.5}$
small bowel	×	$\beta_{\mu 1} \cdot x^2$	$\beta_{\sigma 1} \cdot x^2$
small bowel	✓	$\beta_{\mu 1} \cdot x^2$	$\beta_{\sigma 1} \cdot x^3$
spleen	×	$\beta_{\mu 1} \cdot x^2 + \beta_{\mu 2} \cdot x^3$	$\beta_{\sigma 1} \cdot x^3 + \beta_{\sigma 2} \cdot x^3 \log(x)$
spleen	✓	$\beta_{\mu 1} \cdot x^{-2}$	$\beta_{\sigma 1} \cdot x^3$
sternum	×	$\beta_{\mu 1} \cdot x^{-2} + \beta_{\mu 2} \cdot x^{-2} \log(x) + \beta_{\mu 3} \cdot x^{-1}$	$\beta_{\sigma 1} \cdot x^3 + \beta_{\sigma 2} \cdot x^3 \log(x)$
sternum	✓	$\beta_{\mu 1} \cdot x^{-0.5} + \beta_{\mu 2} \cdot x^{-0.5} \log(x)$	
stomach	×	$\beta_{\mu 1} \cdot x^{-0.5} + \beta_{\mu 2} \cdot x^1$	$\beta_{\sigma 1} \cdot log(x) + \beta_{\sigma 2} \cdot log(x)^2$
stomach	✓	$\beta_{\mu 1} \cdot x^{0.5}$	$\beta_{\sigma 1} \cdot x^3$
subclavian artery l	×	$\beta_{\mu 1} \cdot x^1 + \beta_{\mu 2} \cdot x^2$	$\beta_{\sigma 1} \cdot x^{0.5} + \beta_{\sigma 2} \cdot x^1$
subclavian artery l	✓	$\beta_{\mu 1} \cdot x^{-0.5} + \beta_{\mu 2} \cdot x^{-0.5} \log(x)$	$\beta_{\sigma 1} \cdot x^2 + \beta_{\sigma 2} \cdot x^3$
subclavian artery r	×	$\beta_{\mu 1} \cdot x^2 + \beta_{\mu 2} \cdot x^3$	$\beta_{\sigma 1} \cdot x^2 + \beta_{\sigma 2} \cdot x^2 \log(x)$
subclavian artery r	✓	$\beta_{\mu 1} \cdot x^{-1} + \beta_{\mu 2} \cdot x^{-1} \log(x)$	$\beta_{\sigma 1} \cdot x^3 + \beta_{\sigma 2} \cdot x^3 \log(x)$
superior vena cava	×	$\beta_{\mu 1} \cdot x^{0.5}$	
superior vena cava	✓	$\beta_{\mu 1} \cdot x^3 + \beta_{\mu 2} \cdot x^3 \log(x)$	$\beta_{\sigma 1} \cdot x^3 + \beta_{\sigma 2} \cdot x^3 \log(x)$
thyroid gland	×	$\beta_{\mu 1} \cdot x^2 + \beta_{\mu 2} \cdot x^3$	
thyroid gland	✓	$\beta_{\mu 1} \cdot x^{-0.5} + \beta_{\mu 2} \cdot x^3$	$\beta_{\sigma 1} \cdot x^2$
trachea	×	$\beta_{\mu 1} \cdot x^{-0.5} + \beta_{\mu 2} \cdot x^3 + \beta_{\mu 3} \cdot x^3 \log(x)$	$\beta_{\sigma 1} \cdot x^1 + \beta_{\sigma 2} \cdot x^1 \log(x) + \beta_{\sigma 3} \cdot x^2$
trachea	✓	$\beta_{\mu 1} \cdot x^1 + \beta_{\mu 2} \cdot x^3$	$\beta_{\sigma 1} \cdot x^3 + \beta_{\sigma 2} \cdot x^3 \log(x)$
urinary bladder	×	$\beta_{\mu 1} \cdot log(x)$	$\beta_{\sigma 1} \cdot x^2$
urinary bladder	✓	$\beta_{\mu 1} \cdot x^2 + \beta_{\mu 2} \cdot x^3 + \beta_{\mu 3} \cdot x^3 \log(x)$	$\beta_{\sigma 1} \cdot x^{0.5} + \beta_{\sigma 2} \cdot x^1 + \beta_{\sigma 3} \cdot x^1 \log(x)$
vertebrae C1	×	$\beta_{\mu 1} \cdot log(x) + \beta_{\mu 2} \cdot log(x)^2$	$\beta_{\sigma 1} \cdot log(x)$

Structure	Cont	FP_μ(age)	FP_σ(age)
vertebrae C1	✓	$\beta_{\mu 1} \cdot x^{-1} + \beta_{\mu 2} \cdot x^{-0.5}$	
vertebrae C2	×	$\beta_{\mu 1} \cdot x^{3} + \beta_{\mu 2} \cdot x^{3}\ \log(x)$	$\beta_{\sigma 1} \cdot log(x)$
vertebrae C2	✓	$\beta_{\mu 1} \cdot x^{1}$	
vertebrae C3	×	$\beta_{\mu 1} \cdot x^{1}$	$\beta_{\sigma 1} \cdot x^{-0.5}$
vertebrae C3	✓	$\beta_{\mu 1} \cdot x^{1}$	$\beta_{\sigma 1} \cdot x^{-2}$
vertebrae C4	×	$\beta_{\mu 1} \cdot x^{1}$	$\beta_{\sigma 1} \cdot x^{-0.5}$
vertebrae C4	✓	$\beta_{\mu 1} \cdot x^{1}$	$\beta_{\sigma 1} \cdot x^{-2}$
vertebrae C5	×	$\beta_{\mu 1} \cdot x^{2} + \beta_{\mu 2} \cdot x^{3}$	$\beta_{\sigma 1} \cdot x^{-1}$
vertebrae C5	✓	$\beta_{\mu 1} \cdot x^{1}$	
vertebrae C6	×	$\beta_{\mu 1} \cdot x^{2} + \beta_{\mu 2} \cdot x^{2}\ \log(x)$	$\beta_{\sigma 1} \cdot x^{-1}$
vertebrae C6	✓	$\beta_{\mu 1} \cdot x^{1}$	
vertebrae C7	×	$\beta_{\mu 1} \cdot x^{1}$	$\beta_{\sigma 1} \cdot log(x)$
vertebrae C7	✓	$\beta_{\mu 1} \cdot x^{1}$	
vertebrae L1	×	$\beta_{\mu 1} \cdot x^{1} + \beta_{\mu 2} \cdot x^{2} + \beta_{\mu 3} \cdot x^{2}\ \log(x)$	$\beta_{\sigma 1} \cdot x^{3}$
vertebrae L1	✓	$\beta_{\mu 1} \cdot x^{1} + \beta_{\mu 2} \cdot x^{2} + \beta_{\mu 3} \cdot x^{2}\ \log(x)$	$\beta_{\sigma 1} \cdot x^{3}$
vertebrae L2	×	$\beta_{\mu 1} \cdot x^{3} + \beta_{\mu 2} \cdot x^{3}\ \log(x)$	$\beta_{\sigma 1} \cdot x^{2}$
vertebrae L2	✓	$\beta_{\mu 1} \cdot x^{1} + \beta_{\mu 2} \cdot x^{2} + \beta_{\mu 3} \cdot x^{2}\ \log(x)$	$\beta_{\sigma 1} \cdot x^{3}$
vertebrae L3	×	$\beta_{\mu 1} \cdot x^{3} + \beta_{\mu 2} \cdot x^{3}\ \log(x)$	$\beta_{\sigma 1} \cdot x^{2}$
vertebrae L3	✓	$\beta_{\mu 1} \cdot x^{1} + \beta_{\mu 2} \cdot x^{1}\ \log(x) + \beta_{\mu 3} \cdot x^{2}$	$\beta_{\sigma 1} \cdot x^{3}$
vertebrae L4	×	$\beta_{\mu 1} \cdot x^{-1} + \beta_{\mu 2} \cdot x^{-1}\ \log(x)$	$\beta_{\sigma 1} \cdot x^{2}$
vertebrae L4	✓	$\beta_{\mu 1} \cdot x^{1} + \beta_{\mu 2} \cdot x^{1}\ \log(x) + \beta_{\mu 3} \cdot x^{2}$	$\beta_{\sigma 1} \cdot x^{3}$
vertebrae L5	×	$\beta_{\mu 1} \cdot x^{-1} + \beta_{\mu 2} \cdot x^{-1}\ \log(x)$	$\beta_{\sigma 1} \cdot x^{1}$
vertebrae L5	✓	$\beta_{\mu 1} \cdot x^{1} + \beta_{\mu 2} \cdot x^{1}\ \log(x) + \beta_{\mu 3} \cdot x^{1}\ \log(x)^2$	$\beta_{\sigma 1} \cdot x^{2}$
vertebrae S1	×	$\beta_{\mu 1} \cdot x^{-0.5} + \beta_{\mu 2} \cdot x^{0.5}$	$\beta_{\sigma 1} \cdot x^{1}$
vertebrae S1	✓	$\beta_{\mu 1} \cdot x^{-2} + \beta_{\mu 2} \cdot x^{-2}\ \log(x) + \beta_{\mu 3} \cdot x^{-2}\ \log(x)^2$	$\beta_{\sigma 1} \cdot x^{1}$
vertebrae T1	×	$\beta_{\mu 1} \cdot x^{-0.5} + \beta_{\mu 2} \cdot log(x)$	$\beta_{\sigma 1} \cdot x^{1}$
vertebrae T1	✓	$\beta_{\mu 1} \cdot x^{1}$	
vertebrae T10	×	$\beta_{\mu 1} \cdot x^{-2} + \beta_{\mu 2} \cdot x^{-2}\ \log(x) + \beta_{\mu 3} \cdot x^{-1}$	$\beta_{\sigma 1} \cdot x^{1}$
vertebrae T10	✓	$\beta_{\mu 1} \cdot x^{-2} + \beta_{\mu 2} \cdot x^{-2}\ \log(x) + \beta_{\mu 3} \cdot x^{-2}\ \log(x)^2$	$\beta_{\sigma 1} \cdot x^{0.5}$
vertebrae T11	×	$\beta_{\mu 1} \cdot x^{1} + \beta_{\mu 2} \cdot x^{2} + \beta_{\mu 3} \cdot x^{2}\ \log(x)$	$\beta_{\sigma 1} \cdot x^{2}$
vertebrae T11	✓	$\beta_{\mu 1} \cdot x^{-2} + \beta_{\mu 2} \cdot x^{-2}\ \log(x) + \beta_{\mu 3} \cdot x^{-2}\ \log(x)^2$	$\beta_{\sigma 1} \cdot x^{2}$
vertebrae T12	×	$\beta_{\mu 1} \cdot x^{1} + \beta_{\mu 2} \cdot x^{2} + \beta_{\mu 3} \cdot x^{2}\ \log(x)$	$\beta_{\sigma 1} \cdot x^{2}$
vertebrae T12	✓	$\beta_{\mu 1} \cdot x^{1} + \beta_{\mu 2} \cdot x^{1}\ \log(x) + \beta_{\mu 3} \cdot x^{2}$	$\beta_{\sigma 1} \cdot x^{2}$
vertebrae T2	×	$\beta_{\mu 1} \cdot x^{-2} + \beta_{\mu 2} \cdot x^{-2}\ \log(x) + \beta_{\mu 3} \cdot x^{-1}$	$\beta_{\sigma 1} \cdot x^{1}$
vertebrae T2	✓	$\beta_{\mu 1} \cdot x^{1}$	
vertebrae T3	×	$\beta_{\mu 1} \cdot x^{-2} + \beta_{\mu 2} \cdot x^{-2}\ \log(x) + \beta_{\mu 3} \cdot x^{-1}$	$\beta_{\sigma 1} \cdot x^{0.5}$
vertebrae T3	✓	$\beta_{\mu 1} \cdot x^{-2} + \beta_{\mu 2} \cdot x^{-2}\ \log(x) + \beta_{\mu 3} \cdot x^{-1}$	$\beta_{\sigma 1} \cdot x^{-2}$
vertebrae T4	×	$\beta_{\mu 1} \cdot x^{-2} + \beta_{\mu 2} \cdot x^{-2}\ \log(x) + \beta_{\mu 3} \cdot x^{-1}$	$\beta_{\sigma 1} \cdot x^{0.5}$
vertebrae T4	✓	$\beta_{\mu 1} \cdot x^{-2} + \beta_{\mu 2} \cdot x^{-2}\ \log(x) + \beta_{\mu 3} \cdot x^{-1}$	$\beta_{\sigma 1} \cdot x^{-2}$
vertebrae T5	×	$\beta_{\mu 1} \cdot x^{-2} + \beta_{\mu 2} \cdot x^{-2}\ \log(x) + \beta_{\mu 3} \cdot x^{-0.5}$	$\beta_{\sigma 1} \cdot x^{0.5}$
vertebrae T5	✓	$\beta_{\mu 1} \cdot x^{-2} + \beta_{\mu 2} \cdot x^{-2}\ \log(x) + \beta_{\mu 3} \cdot x^{-1}$	$\beta_{\sigma 1} \cdot x^{-2}$
vertebrae T6	×	$\beta_{\mu 1} \cdot x^{-2} + \beta_{\mu 2} \cdot x^{-1} + \beta_{\mu 3} \cdot x^{-1}\ \log(x)$	$\beta_{\sigma 1} \cdot x^{0.5}$
vertebrae T6	✓	$\beta_{\mu 1} \cdot x^{-2} + \beta_{\mu 2} \cdot x^{-2}\ \log(x) + \beta_{\mu 3} \cdot x^{-2}\ \log(x)^2$	$\beta_{\sigma 1} \cdot x^{-1}$
vertebrae T7	×	$\beta_{\mu 1} \cdot x^{-2} + \beta_{\mu 2} \cdot x^{-1} + \beta_{\mu 3} \cdot x^{-1}\ \log(x)$	$\beta_{\sigma 1} \cdot x^{0.5}$
vertebrae T7	✓	$\beta_{\mu 1} \cdot x^{-2} + \beta_{\mu 2} \cdot x^{-2}\ \log(x) + \beta_{\mu 3} \cdot x^{-2}\ \log(x)^2$	$\beta_{\sigma 1} \cdot x^{-0.5}$
vertebrae T8	×	$\beta_{\mu 1} \cdot x^{-2} + \beta_{\mu 2} \cdot x^{-1} + \beta_{\mu 3} \cdot x^{-1}\ \log(x)$	$\beta_{\sigma 1} \cdot x^{1}$
vertebrae T8	✓	$\beta_{\mu 1} \cdot x^{-2} + \beta_{\mu 2} \cdot x^{-2}\ \log(x) + \beta_{\mu 3} \cdot x^{-2}\ \log(x)^2$	$\beta_{\sigma 1} \cdot x^{-0.5}$
vertebrae T9	×	$\beta_{\mu 1} \cdot x^{-2} + \beta_{\mu 2} \cdot x^{-1} + \beta_{\mu 3} \cdot x^{-1}\ \log(x)$	$\beta_{\sigma 1} \cdot x^{1}$
vertebrae T9	✓	$\beta_{\mu 1} \cdot x^{-2} + \beta_{\mu 2} \cdot x^{-2}\ \log(x) + \beta_{\mu 3} \cdot x^{-2}\ \log(x)^2$	$\beta_{\sigma 1} \cdot x^{-0.5}$

Supplementary Table 7: Fractional polynomial (FP) models for non-linear age effects in the μ (location) and σ (scale) components of the ST1 distribution with age x and contrast status (Cont). For each structure, FP functions, selected from the set $\{-2, -1, -0.5, 0, 0.5, 1, 2, 3\}$ and fitted using one to three terms, were chosen based on the Bayesian information criterion (BIC). These FP functions enable flexible modeling of the age effect while balancing model fit and complexity.

Regression coefficients for longitudinal volume models

Supplementary Table 8 provides the full set of longitudinal fixed-effect estimates of volume models for all anatomical structures, using follow-up time time_b (years) and baseline age age_b. Coefficients are reported for the main longitudinal term (time_b) and the two effect-modification terms, consistent with the notation in the main text.

Structure	time_b	$\text{time}_b : \text{age}_b$	$\text{time}_b : \text{sex}_\text{M}$
adrenal gland left	**1.0387**	**0.9944**	**0.9922**
adrenal gland right	**1.0294**	**0.9954**	**0.9963**
aorta	**1.0307**	**0.9965**	1.0000
atrial appendage left	**0.9768**	**1.0040**	**1.0027**
brachiocephalic trunk	**1.0105**	**0.9993**	0.9990
brachiocephalic vein left	1.0048	**0.9970**	0.9982
brachiocephalic vein right	**0.9915**	0.9994	**0.9949**
brain	0.9987	1.0001	1.0000
clavicula left	1.0031	0.9999	**1.0043**
clavicula right	**1.0051**	0.9995	**1.0030**
colon	0.9906	1.0007	1.0047
common carotid artery left	**1.0134**	0.9999	**0.9965**
common carotid artery right	**1.0280**	0.9985	0.9956
costal cartilages	**1.0119**	**0.9984**	1.0013
duodenum	**1.0345**	**0.9942**	0.9970
esophagus	**1.0151**	**0.9983**	0.9982
gallbladder	1.0121	**0.9966**	0.9970
gluteus maximus left	1.0100	**0.9962**	1.0025
gluteus maximus right	**1.0113**	**0.9962**	1.0004
gluteus medius left	**1.0128**	**0.9963**	**0.9984**
gluteus medius right	**1.0148**	**0.9960**	**0.9970**
gluteus minimus left	**1.0046**	**0.9982**	1.0005
gluteus minimus right	**1.0035**	**0.9985**	**0.9985**
heart	**0.9932**	1.0006	1.0013
hip left	**1.0037**	**0.9997**	0.9999
hip right	**1.0033**	**0.9997**	0.9998
iliac artery left	**1.0152**	**0.9991**	1.0005
iliac artery right	**1.0152**	**0.9985**	1.0007
iliac vena left	**1.0174**	**0.9955**	0.9989
iliac vena right	**1.0177**	**0.9956**	0.9990
iliopsoas left	1.0034	**0.9970**	**0.9951**
iliopsoas right	1.0019	**0.9974**	**0.9939**
inferior vena cava	1.0037	**0.9983**	0.9975
kidney left	**1.0098**	**0.9953**	1.0008
kidney right	**1.0137**	**0.9950**	0.9986
liver	**1.0080**	**0.9971**	**0.9956**
lung lower lobe left	**1.0235**	**0.9948**	1.0008
lung lower lobe right	**1.0201**	**0.9958**	1.0000
lung middle lobe right	**1.0078**	**0.9977**	**1.0042**
lung upper lobe left	**1.0082**	**0.9983**	1.0021
lung upper lobe right	**1.0118**	**0.9982**	0.9996
pancreas	**1.0225**	**0.9933**	0.9996
portal vein and splenic vein	**1.0316**	**0.9952**	1.0010
pulmonary vein	0.9993	1.0000	1.0003
rib left 1	**1.0198**	**0.9976**	0.9989
rib left 10	**1.0030**	1.0000	1.0002

Structure	$time_b$	$time_b : age_b$	$time_b : sex_M$
rib left 11	**1.0022**	**1.0000**	1.0000
rib left 12	0.9988	1.0000	**1.0020**
rib left 2	0.9999	1.0006	**1.0011**
rib left 3	**0.9975**	**1.0010**	**1.0011**
rib left 4	**0.9965**	**1.0011**	**1.0012**
rib left 5	0.9983	**1.0006**	**1.0025**
rib left 6	**1.0014**	1.0001	**1.0024**
rib left 7	**1.0017**	**1.0002**	**1.0012**
rib left 8	1.0003	1.0004	**1.0015**
rib left 9	**1.0019**	1.0002	1.0006
rib right 1	**1.0149**	**0.9984**	**0.9988**
rib right 10	**1.0060**	0.9997	1.0002
rib right 11	**1.0033**	1.0000	**0.9999**
rib right 12	**0.9959**	**1.0010**	0.9999
rib right 2	1.0004	1.0006	**1.0009**
rib right 3	**0.9978**	**1.0009**	**1.0020**
rib right 4	**0.9974**	**1.0009**	**1.0023**
rib right 5	**0.9972**	**1.0008**	**1.0032**
rib right 6	0.9990	**1.0005**	**1.0023**
rib right 7	**1.0025**	1.0000	1.0006
rib right 8	1.0025	1.0001	1.0006
rib right 9	1.0029	1.0001	**1.0012**
sacrum	**1.0024**	**0.9998**	**1.0006**
scapula left	1.0017	0.9996	**1.0032**
scapula right	1.0022	0.9997	**1.0019**
small bowel	**1.0182**	**0.9963**	**0.9974**
spleen	1.0022	**0.9992**	**0.9940**
sternum	**1.0100**	**0.9990**	1.0004
stomach	0.9964	**0.9974**	0.9964
subclavian artery left	**1.0225**	**0.9977**	**0.9979**
subclavian artery right	**1.0254**	**0.9975**	**0.9971**
superior vena cava	**0.9913**	**1.0008**	1.0002
thyroid gland	**1.0083**	**0.9960**	**0.9921**
trachea	**1.0121**	**0.9989**	0.9998
urinary bladder	0.9902	0.9998	1.0052
vertebrae C1	1.0035	0.9995	1.0016
vertebrae C2	**1.0029**	0.9998	1.0004
vertebrae C3	1.0021	1.0000	**1.0027**
vertebrae C4	**1.0032**	1.0001	1.0003
vertebrae C5	**1.0034**	1.0000	1.0014
vertebrae C6	1.0003	**1.0006**	**1.0024**
vertebrae C7	1.0022	0.9999	1.0029
vertebrae L1	1.0012	1.0001	1.0011
vertebrae L2	**1.0049**	**0.9993**	**1.0022**
vertebrae L3	1.0012	1.0002	1.0001
vertebrae L4	1.0017	1.0000	1.0012
vertebrae L5	**1.0029**	0.9998	**1.0012**
vertebrae S1	1.0043	0.9995	1.0005
vertebrae T1	**1.0028**	0.9999	1.0012
vertebrae T10	1.0024	0.9999	1.0010
vertebrae T11	**1.0034**	0.9997	1.0011
vertebrae T12	**1.0022**	1.0000	1.0004
vertebrae T2	1.0010	1.0001	**1.0013**
vertebrae T3	**1.0026**	0.9997	1.0011
vertebrae T4	**1.0033**	0.9996	1.0010
vertebrae T5	**1.0035**	**0.9995**	**1.0017**
vertebrae T6	1.0031	0.9998	1.0005

Structure	time_b	$\text{time}_b : \text{age}_b$	$\text{time}_b : \text{sex}_\text{M}$
vertebrae T7	1.0049	0.9996	0.9995
vertebrae T8	**1.0048**	0.9996	1.0008
vertebrae T9	1.0024	0.9999	1.0012

Supplementary Table 8: Longitudinal fixed-effect coefficient estimates for each anatomical structure for the location parameter (μ, log link). Columns report the main follow-up term (time_b, in years) and two effect-modification terms ($\text{time}_b : \text{age}_b$ and $\text{time}_b : \text{sex}_\text{M}$, male vs female). Estimates are shown as $\exp(\hat{\beta})$ and represent multiplicative effects on μ per 1-year increase in time_b; interaction terms indicate how the longitudinal slope is modified by baseline age and sex. Values > 1 indicate an increase and values < 1 a decrease in μ relative to the reference. Boldface marks Bonferroni-adjusted $p < 0.05$.

Regression coefficients for longitudinal attenuation models

Structure	Contrast	time_b	$\text{time}_b : \text{age}_b$	$\text{time}_b : \text{sex}_\text{M}$
adrenal gland left	×	**-1.0114**	0.0036	-0.2238
adrenal gland left	✓	-0.5079	**0.2601**	-0.2530
adrenal gland right	×	**-1.1326**	0.0327	-0.2178
adrenal gland right	✓	-0.1473	**0.1976**	-0.3402
aorta	×	-0.6994	0.0812	-0.0330
aorta	✓	-0.2304	**0.4402**	0.4245
atrial appendage left	×	**-1.2998**	**0.1474**	-0.0720
atrial appendage left	✓	0.3717	0.1983	0.0214
brachiocephalic trunk	×	-0.4750	0.0579	0.2545
brachiocephalic trunk	✓	0.4178	**0.2362**	-0.0570
brachiocephalic vein left	×	-0.3144	-0.0217	0.0147
brachiocephalic vein left	✓	**2.4438**	0.0334	0.4089
brachiocephalic vein right	×	-0.5555	0.0740	-0.0561
brachiocephalic vein right	✓	0.6774	0.3667	0.7140
brain	×	0.2813	**-0.0972**	0.1061
brain	✓	1.7356	-0.3503	-0.0774
clavicula left	×	**-6.7515**	-0.3659	2.5915
clavicula left	✓	-2.1648	-0.3090	0.4636
clavicula right	×	**-6.4275**	-0.2436	2.3591
clavicula right	✓	-2.4236	-0.1180	-0.6813
colon	×	4.7380	-0.7465	0.2016
colon	✓	0.2451	-0.7439	0.3675
common carotid artery left	×	-0.9001	0.0533	0.1823
common carotid artery left	✓	-0.9001	0.1651	1.1921
common carotid artery right	×	-0.9197	0.0262	0.1764
common carotid artery right	✓	0.8092	-0.0564	**1.2031**
costal cartilages	×	-1.2088	0.1780	**-0.5872**
costal cartilages	✓	**-0.5362**	**0.1512**	**-0.6566**
duodenum	×	0.7930	-0.3261	-0.3595
duodenum	✓	1.6153	-0.3431	-0.0529
esophagus	×	-0.0225	-0.1196	-0.0610
esophagus	✓	0.6958	-0.0824	-0.2772
gallbladder	×	-0.0450	-0.0452	0.0091
gallbladder	✓	-0.5970	0.0886	0.1678
gluteus maximus left	×	0.3616	-0.1416	-0.0954
gluteus maximus left	✓	-0.3912	-0.0413	-0.5478
gluteus maximus right	×	-0.2707	-0.0238	-0.1188
gluteus maximus right	✓	-1.5355	0.0972	-0.1383
gluteus medius left	×	-0.0960	-0.1180	0.2543
gluteus medius left	✓	-0.2226	**-0.0988**	0.1447
gluteus medius right	×	-0.0748	-0.0980	0.2094
gluteus medius right	✓	-0.2306	**-0.0833**	0.1045
gluteus minimus left	×	0.6324	**-0.3457**	0.3768
gluteus minimus left	✓	**0.6549**	**-0.3828**	**0.3201**
gluteus minimus right	×	**0.9243**	**-0.3857**	0.2073
gluteus minimus right	✓	**0.6393**	**-0.3701**	**0.2007**
heart	×	0.0131	-0.0077	-0.0895
heart	✓	0.6890	0.1562	0.3690
hip left	×	-2.4461	-0.3139	1.5883
hip left	✓	0.1316	**-0.3064**	**-0.8163**
hip right	×	-2.4191	-0.2980	1.6043
hip right	✓	0.7093	**-0.3513**	**-0.8224**
iliac artery left	×	**-1.3579**	**0.2054**	0.1475

Structure	Contrast	$time_b$	$time_b : age_b$	$time_b : sex_M$
iliac artery left	✓	-1.5224	**0.6727**	**0.8606**
iliac artery right	×	-0.9773	0.1480	-0.0020
iliac artery right	✓	-1.0548	**0.5994**	**1.1177**
iliac vena left	×	-0.0959	-0.0498	-0.0842
iliac vena left	✓	**1.4100**	-0.1036	**-0.4601**
iliac vena right	×	0.0580	-0.0743	-0.2378
iliac vena right	✓	**1.4621**	-0.1248	**-0.4695**
iliopsoas left	×	-0.2350	-0.0538	-0.2394
iliopsoas left	✓	-0.0908	**-0.0520**	**-0.1535**
iliopsoas right	×	-0.2159	-0.0455	-0.2920
iliopsoas right	✓	-0.0505	**-0.0582**	-0.0962
inferior vena cava	×	-0.2667	-0.0520	-0.4522
inferior vena cava	✓	1.1406	0.0713	0.1313
kidney left	×	-0.6059	-0.0543	-0.1286
kidney left	✓	**2.3792**	-0.1591	0.7244
kidney right	×	-0.7540	-0.0233	-0.0690
kidney right	✓	**2.1986**	-0.0965	0.6538
liver	×	**-2.1696**	**0.2372**	0.0548
liver	✓	1.2836	-0.0287	0.1422
lung lower lobe left	×	-1.9165	0.0361	0.1327
lung lower lobe left	✓	**-4.6662**	**0.7815**	-0.2740
lung lower lobe right	×	-0.8061	-0.1223	0.0455
lung lower lobe right	✓	-2.7042	0.5205	-0.5404
lung middle lobe right	×	-0.9293	-0.1156	-0.1283
lung middle lobe right	✓	**-2.4312**	**0.4016**	-0.6717
lung upper lobe left	×	-1.7073	0.0897	-0.4285
lung upper lobe left	✓	**-2.4492**	0.3267	-0.2192
lung upper lobe right	×	-1.9524	0.1189	-0.2383
lung upper lobe right	✓	-2.1170	0.2585	-0.1668
pancreas	×	**-1.2523**	0.0098	-0.0232
pancreas	✓	-0.1479	0.1304	-0.0227
portal vein and splenic vein	×	-0.3656	-0.0593	-0.0131
portal vein and splenic vein	✓	**1.7174**	0.1657	-0.1851
pulmonary vein	×	0.2533	-0.0158	-0.0603
pulmonary vein	✓	**1.5728**	0.0288	-0.0169
rib left 1	×	2.6923	**-1.3078**	1.4775
rib left 1	✓	0.0814	**-0.7710**	-0.2709
rib left 10	×	**-7.2367**	-0.1490	**4.7951**
rib left 10	✓	**-3.5117**	-0.0827	**1.1594**
rib left 11	×	-4.5230	-0.7881	**4.0436**
rib left 11	✓	**-4.0952**	-0.0387	0.8353
rib left 12	×	1.2863	**-1.3199**	**2.9786**
rib left 12	✓	**-4.2995**	-0.0306	0.8482
rib left 2	×	-2.9096	-0.2846	**2.7536**
rib left 2	✓	**-4.7290**	0.2213	0.1270
rib left 3	×	**-6.1368**	0.2091	**2.4526**
rib left 3	✓	**-5.4268**	**0.4533**	0.0674
rib left 4	×	**-6.2416**	0.1133	**2.7158**
rib left 4	✓	**-4.4590**	0.3221	0.1675
rib left 5	×	**-6.5397**	0.1035	**2.9488**
rib left 5	✓	**-3.9685**	0.2222	0.3116
rib left 6	×	**-5.8198**	-0.0441	**2.8623**
rib left 6	✓	**-3.2501**	0.0480	0.6677
rib left 7	×	**-5.9712**	-0.0700	**3.1224**
rib left 7	✓	**-3.1082**	-0.0175	**1.0552**
rib left 8	×	**-6.2971**	-0.1573	**3.8240**
rib left 8	✓	**-3.2171**	-0.0702	**1.1428**

Structure	Contrast	$time_b$	$time_b : age_b$	$time_b : sex_M$
rib left 9	×	**-5.8014**	-0.3199	**4.0958**
rib left 9	✓	**-3.5440**	-0.0640	**1.0733**
rib right 1	×	1.8938	**-1.1639**	1.7231
rib right 1	✓	-2.2433	-0.3155	-1.0109
rib right 10	×	**-7.1081**	-0.1472	**3.8227**
rib right 10	✓	**-3.7338**	0.0710	0.9513
rib right 11	×	-4.6742	-0.8774	**4.5673**
rib right 11	✓	**-3.6295**	-0.0375	0.8408
rib right 12	×	2.3164	**-1.4849**	2.7902
rib right 12	✓	**-2.6514**	-0.1282	0.3274
rib right 2	×	-3.4175	-0.2507	**2.6783**
rib right 2	✓	**-4.6154**	0.2215	0.2609
rib right 3	×	**-5.4836**	0.0154	**2.6217**
rib right 3	✓	**-4.7963**	**0.3975**	-0.1111
rib right 4	×	**-5.8113**	0.1038	**2.0644**
rib right 4	✓	**-3.8031**	0.3126	-0.2977
rib right 5	×	**-6.0404**	0.1144	**2.2690**
rib right 5	✓	**-2.6190**	0.0987	0.1568
rib right 6	×	**-5.3530**	-0.0320	**2.3285**
rib right 6	✓	**-2.4647**	0.0133	0.4927
rib right 7	×	**-7.0846**	0.0686	**3.2976**
rib right 7	✓	-2.3047	-0.0394	0.6283
rib right 8	×	**-6.0511**	-0.2299	**3.6496**
rib right 8	✓	-2.2543	-0.1452	0.8100
rib right 9	×	**-5.1712**	-0.4044	**3.8715**
rib right 9	✓	**-2.9885**	-0.0627	0.8300
sacrum	×	**-2.7503**	-0.2543	**2.0216**
sacrum	✓	**-2.0318**	0.0013	-0.0102
scapula left	×	-0.7569	-0.5856	1.7181
scapula left	✓	-0.9753	0.0058	-0.5191
scapula right	×	0.3641	**-0.7540**	1.4364
scapula right	✓	-1.3690	-0.0121	-1.1295
small bowel	×	-0.9748	-0.0782	0.4150
small bowel	✓	**-5.8794**	0.1212	**1.8705**
spleen	×	-0.0177	-0.0476	-0.1173
spleen	✓	**1.1313**	0.0382	0.1776
sternum	×	**-2.5563**	0.0685	0.8966
sternum	✓	-0.5344	-0.1182	-0.2474
stomach	×	2.6874	-0.4637	-0.7481
stomach	✓	-1.5623	0.2985	1.3610
subclavian artery left	×	-0.6895	**0.1240**	-0.0706
subclavian artery left	✓	0.2643	**0.1957**	-0.0127
subclavian artery right	×	**-1.0458**	**0.1317**	-0.0924
subclavian artery right	✓	-0.2026	**0.2559**	-0.2311
superior vena cava	×	-0.0642	-0.0104	-0.1628
superior vena cava	✓	1.1742	0.2782	0.5345
thyroid gland	×	**-1.6534**	0.1002	0.1863
thyroid gland	✓	1.4859	-0.0214	0.0420
trachea	×	**-3.3625**	0.3772	0.1580
trachea	✓	-0.8868	0.1057	-0.1195
urinary bladder	×	0.0826	-0.0894	0.0196
urinary bladder	✓	0.1509	-0.0454	0.0916
vertebrae C1	×	6.2020	**-1.4674**	-0.3777
vertebrae C1	✓	**-6.9108**	0.4804	**2.6975**
vertebrae C2	×	**10.1343**	**-2.2543**	-0.7542
vertebrae C2	✓	**-6.6775**	0.2670	**2.9296**
vertebrae C3	×	6.0746	**-1.5420**	-0.5309

Structure	Contrast	$time_b$	$time_b : age_b$	$time_b : sex_M$
vertebrae C3	✓	**-6.9901**	0.4171	**2.8246**
vertebrae C4	×	**7.6718**	**-1.7262**	-1.9144
vertebrae C4	✓	**-7.3745**	0.5549	2.2356
vertebrae C5	×	5.8559	-1.2418	-2.2045
vertebrae C5	✓	**-6.5322**	0.3701	2.0040
vertebrae C6	×	4.3729	-1.0685	-1.0949
vertebrae C6	✓	**-6.6921**	0.4166	1.5907
vertebrae C7	×	0.6966	-0.3170	-1.2827
vertebrae C7	✓	-4.8120	0.2437	1.6438
vertebrae L1	×	1.8618	**-0.9874**	1.9875
vertebrae L1	✓	-1.8126	-0.1137	**1.6218**
vertebrae L2	×	-0.2141	-0.7627	1.9914
vertebrae L2	✓	-1.2356	-0.2804	**1.7805**
vertebrae L3	×	-0.7631	-0.8162	1.9235
vertebrae L3	✓	-0.9732	-0.3075	**1.6543**
vertebrae L4	×	-0.6960	-0.6316	0.9027
vertebrae L4	✓	-0.8336	-0.3009	**1.6385**
vertebrae L5	×	-1.3732	**-0.8935**	**3.2698**
vertebrae L5	✓	-0.5269	-0.2831	**1.6482**
vertebrae S1	×	-1.9414	-0.6641	3.1365
vertebrae S1	✓	-0.2157	**-0.5122**	**2.0871**
vertebrae T1	×	1.3373	-0.7684	1.5500
vertebrae T1	✓	-2.2523	-0.0979	0.6209
vertebrae T10	×	0.6440	**-0.8638**	2.5091
vertebrae T10	✓	1.3752	**-0.6755**	**1.1254**
vertebrae T11	×	0.3853	**-0.8230**	2.2602
vertebrae T11	✓	1.0161	**-0.5655**	0.9885
vertebrae T12	×	1.2400	**-0.8912**	2.4618
vertebrae T12	✓	0.5325	**-0.4732**	**1.2517**
vertebrae T2	×	1.4262	-0.7328	2.0193
vertebrae T2	✓	-1.1974	-0.2069	0.8691
vertebrae T3	×	2.0343	**-0.9732**	2.3988
vertebrae T3	✓	0.2416	**-0.4967**	**1.3521**
vertebrae T4	×	1.8942	**-1.0844**	**2.5932**
vertebrae T4	✓	0.6363	**-0.5881**	**1.4733**
vertebrae T5	×	1.8204	**-1.0378**	2.0533
vertebrae T5	✓	2.0016	**-0.7614**	0.8190
vertebrae T6	×	2.8499	**-1.1916**	1.8958
vertebrae T6	✓	1.7566	**-0.6313**	0.1520
vertebrae T7	×	0.9936	**-0.9478**	**2.8073**
vertebrae T7	✓	1.6747	**-0.7416**	0.9993
vertebrae T8	×	0.4724	-0.7912	**2.8973**
vertebrae T8	✓	1.6917	**-0.7193**	1.0449
vertebrae T9	×	0.9630	-0.7616	1.5904
vertebrae T9	✓	1.3897	**-0.6983**	**1.3496**

Supplementary Table 9: GAMM4 longitudinal fixed-effect estimates for each anatomical structure, fit separately for non-contrast and contrast-enhanced scans. Columns report the main follow-up term ($time_b$, in years) and two effect-modification terms ($time_b : age_b$ and $time_b : sex_M$, male vs female) that capture how within-subject change over time varies with baseline age and sex. Because the GAMM4 models use an identity link, estimates are shown on the original outcome scale and represent additive changes (HU) per 1-year increase in $time_b$. Boldface marks Bonferroni-adjusted $p < 0.05$.

References

[1] Jeffrey M. Meier, Abass Alavi, Sireesha Iruvuri, Saad Alzeair, Rex Parker, Mohamed Houseni, Miguel Hernandez-Pampaloni, Andrew Mong, and Drew A. Torigian. Assessment of Age-Related Changes in Abdominal Organ Structure and Function With Computed Tomography and Positron Emission Tomography. *Seminars in Nuclear Medicine*, 37(3):154–172, May 2007.

[2] Luke Hahn, Scott B. Reeder, Alejandro Muñoz del Rio, and Perry J. Pickhardt. Longitudinal Changes in Liver Fat Content in Asymptomatic Adults: Hepatic Attenuation on Unenhanced CT as an Imaging Biomarker for Steatosis. *AJR. American journal of roentgenology*, 205(6):1167–1172, December 2015.

[3] Pedro Figueiredo, Elisa A. Marques, Vilmundur Gudnason, Thomas Lang, Sigurdur Sigurdsson, Palmi V. Jonsson, Thor Aspelund, Kristin Siggeirsdottir, Lenore Launer, Gudny Eiriksdottir, and Tamara B. Harris. Computed tomography-based skeletal muscle and adipose tissue attenuation: Variations by age, sex, and muscle. *Experimental gerontology*, 149:111306, July 2021.

[4] Peter M. Graffy, Jiamin Liu, Perry J. Pickhardt, Joseph E. Burns, Jianhua Yao, and Ronald M. Summers. Deep learning-based muscle segmentation and quantification at abdominal CT: Application to a longitudinal adult screening cohort for sarcopenia assessment. *The British Journal of Radiology*, 92(1100):20190327, August 2019.

[5] Robert D. Boutin, Justin M. Kaptuch, Cyrus P. Bateni, James S. Chalfant, and Lawrence Yao. Influence of IV Contrast Administration on CT Measures of Muscle and Bone Attenuation: Implications for Sarcopenia and Osteoporosis Evaluation. *AJR. American journal of roentgenology*, 207(5):1046–1054, November 2016.

[6] Samuel Jang, Peter M. Graffy, Timothy J. Ziemlewicz, Scott J. Lee, Ronald M. Summers, and Perry J. Pickhardt. Opportunistic Osteoporosis Screening at Routine Abdominal and Thoracic CT: Normative L1 Trabecular Attenuation Values in More than 20 000 Adults. *Radiology*, 291(2):360–367, May 2019.
